# Supplementary figures and images for: Repression of PRMT activities sensitize human homologous recombination-proficient ovarian and breast cancer cells to PARP inhibitor treatment
Source: eLife. 2026 Feb 3;13:RP99225. doi: 10.7554/eLife.99225 (PMC12867483; doi:10.7554/eLife.99225)

Figure 2

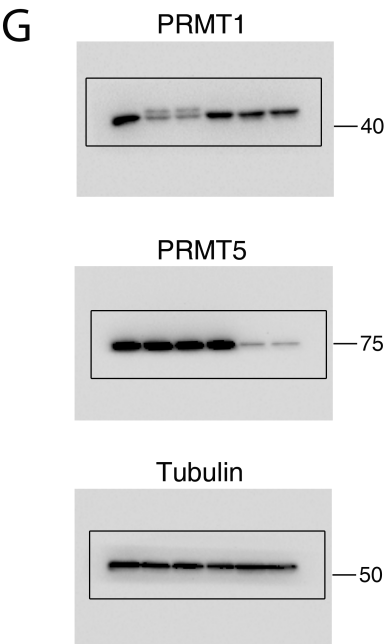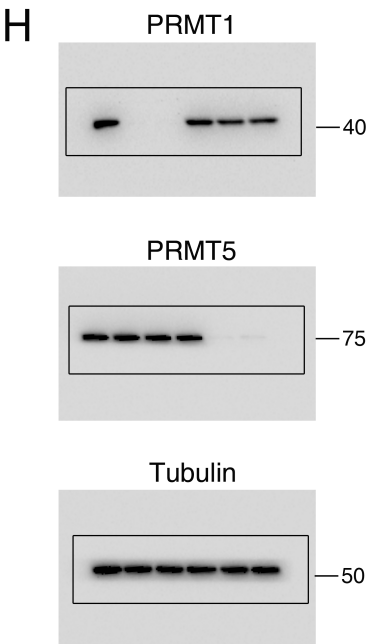

Supplement: Figure 2—source data 1. [file elife-99225-fig2-data1.pdf]

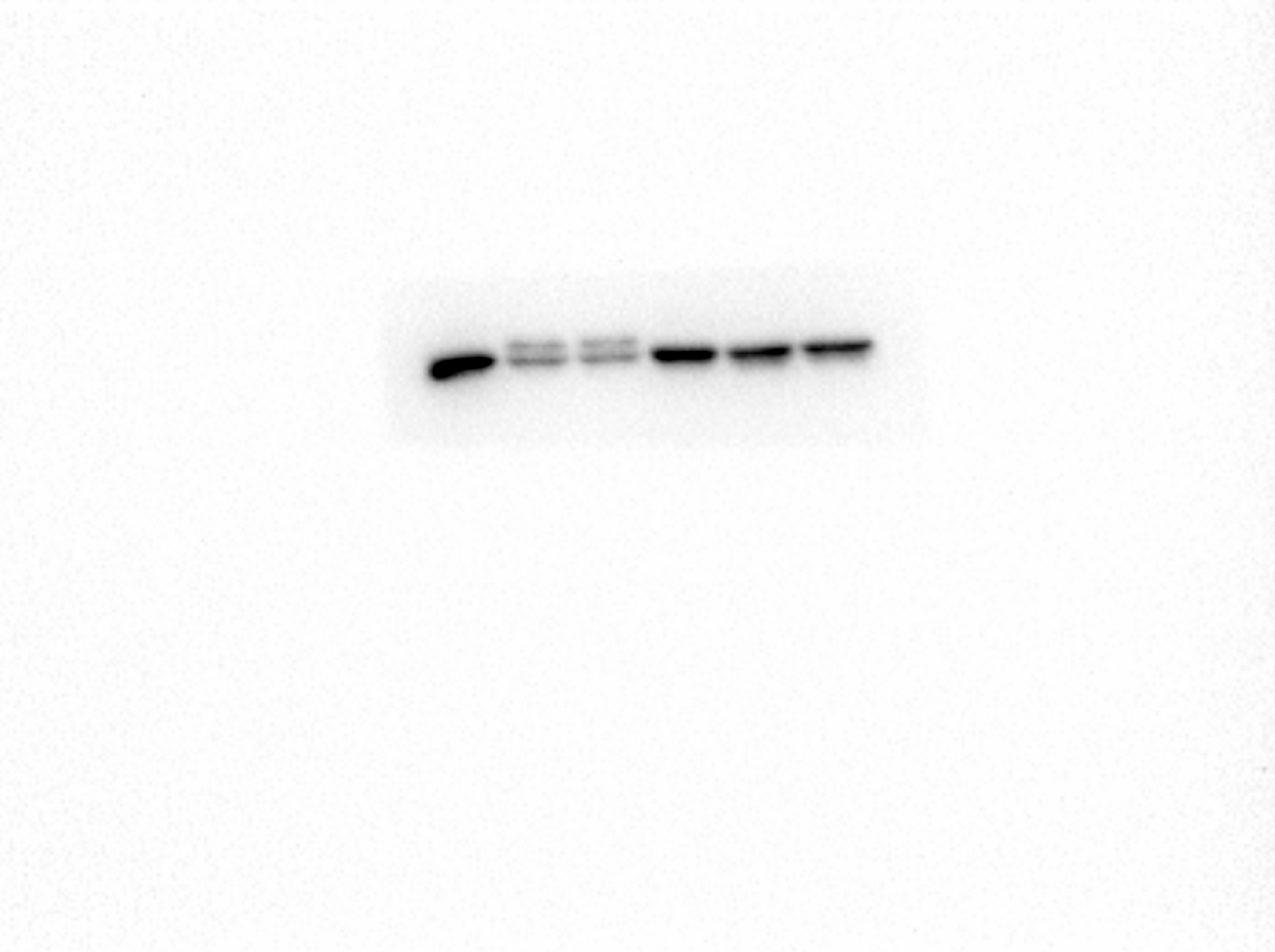

Supplement: Figure 2—source data 2. [file elife-99225-fig2-data2.zip › Figure 2-source data 2/G/prmt1-ov8-prmtsg.tif]

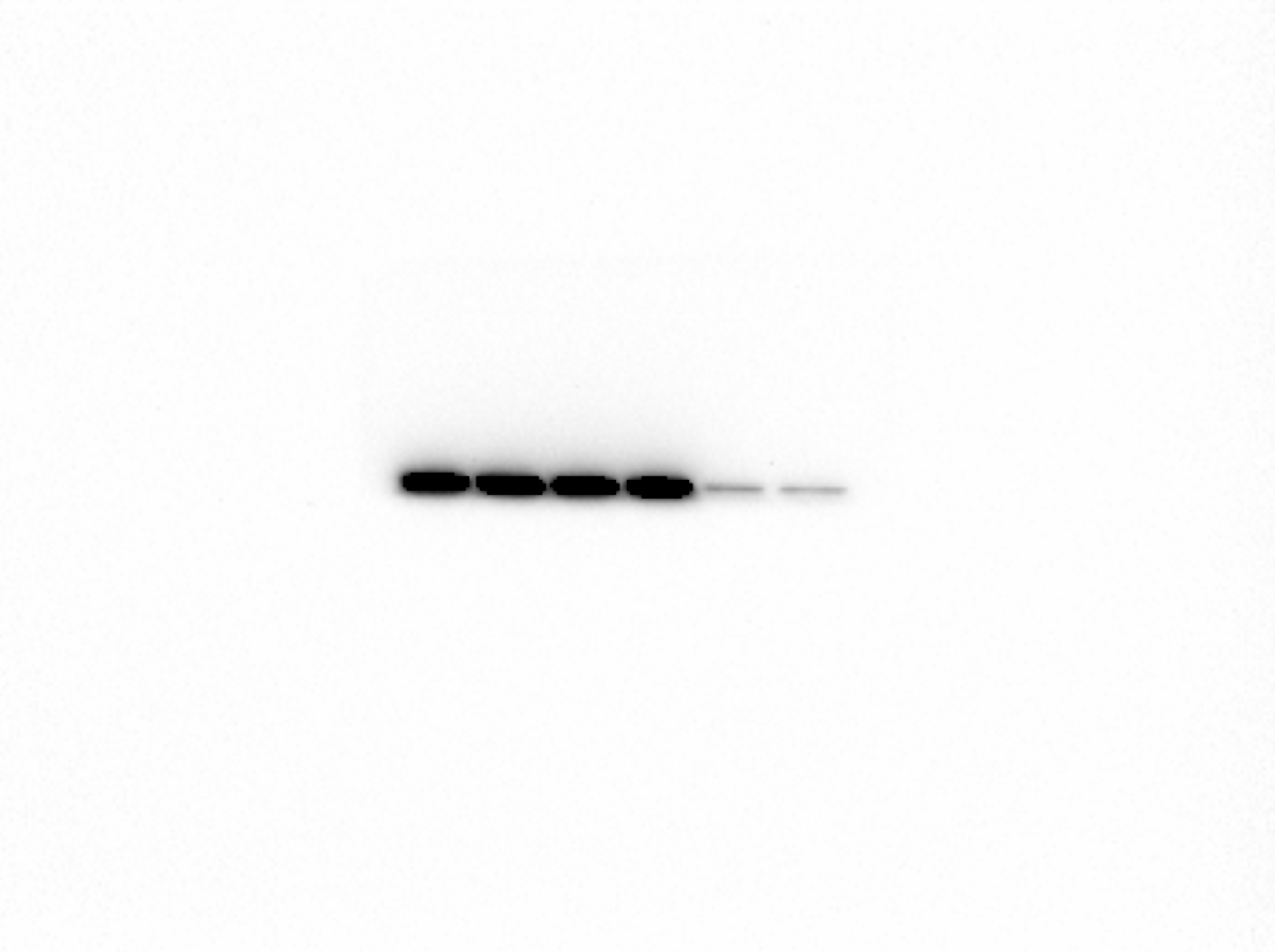

Supplement: Figure 2—source data 2. [file elife-99225-fig2-data2.zip › Figure 2-source data 2/G/prmt5-ov8-prmtsg.tif]

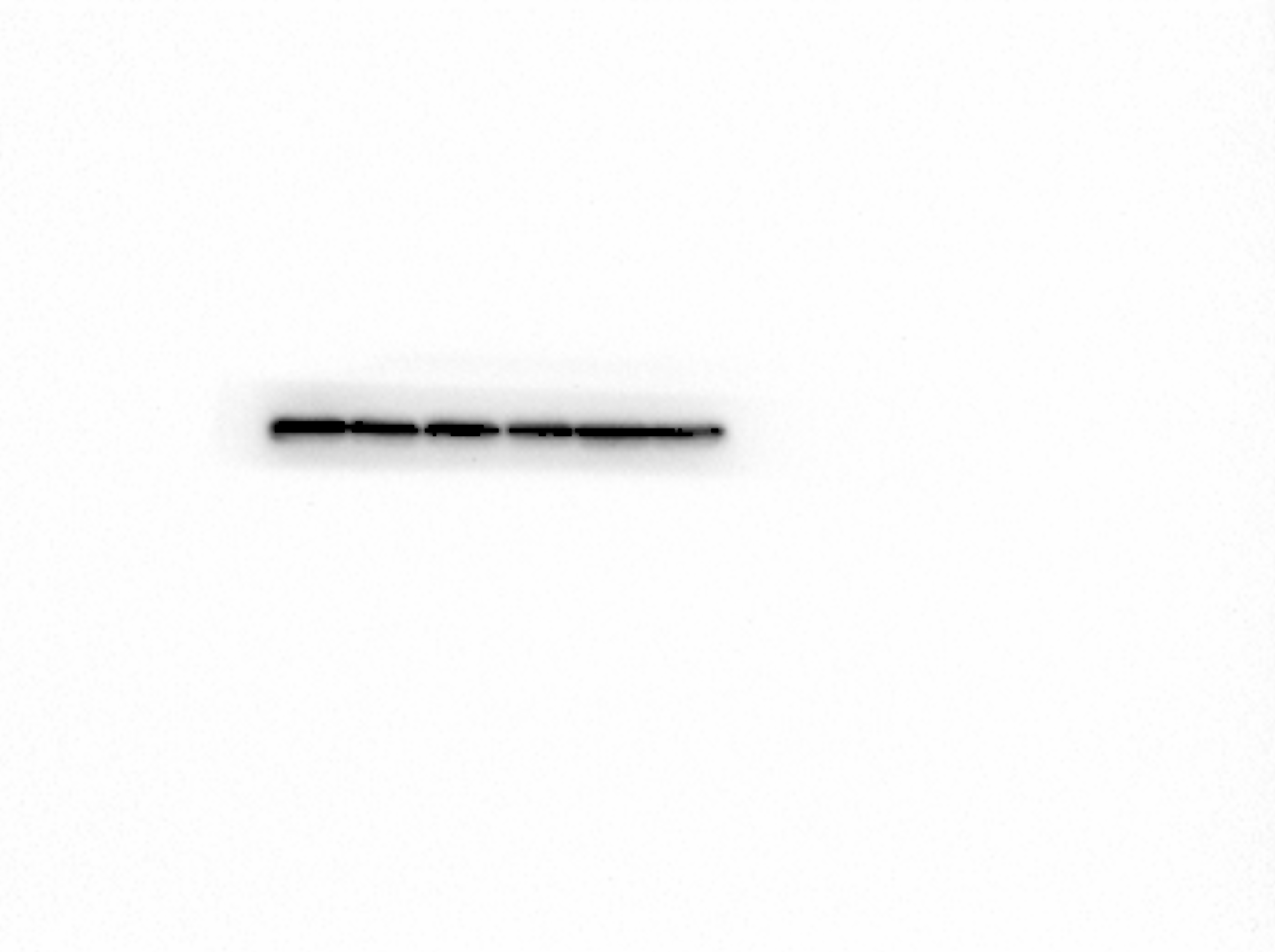

Supplement: Figure 2—source data 2. [file elife-99225-fig2-data2.zip › Figure 2-source data 2/G/tublin-ov8-prmtsg.tif]

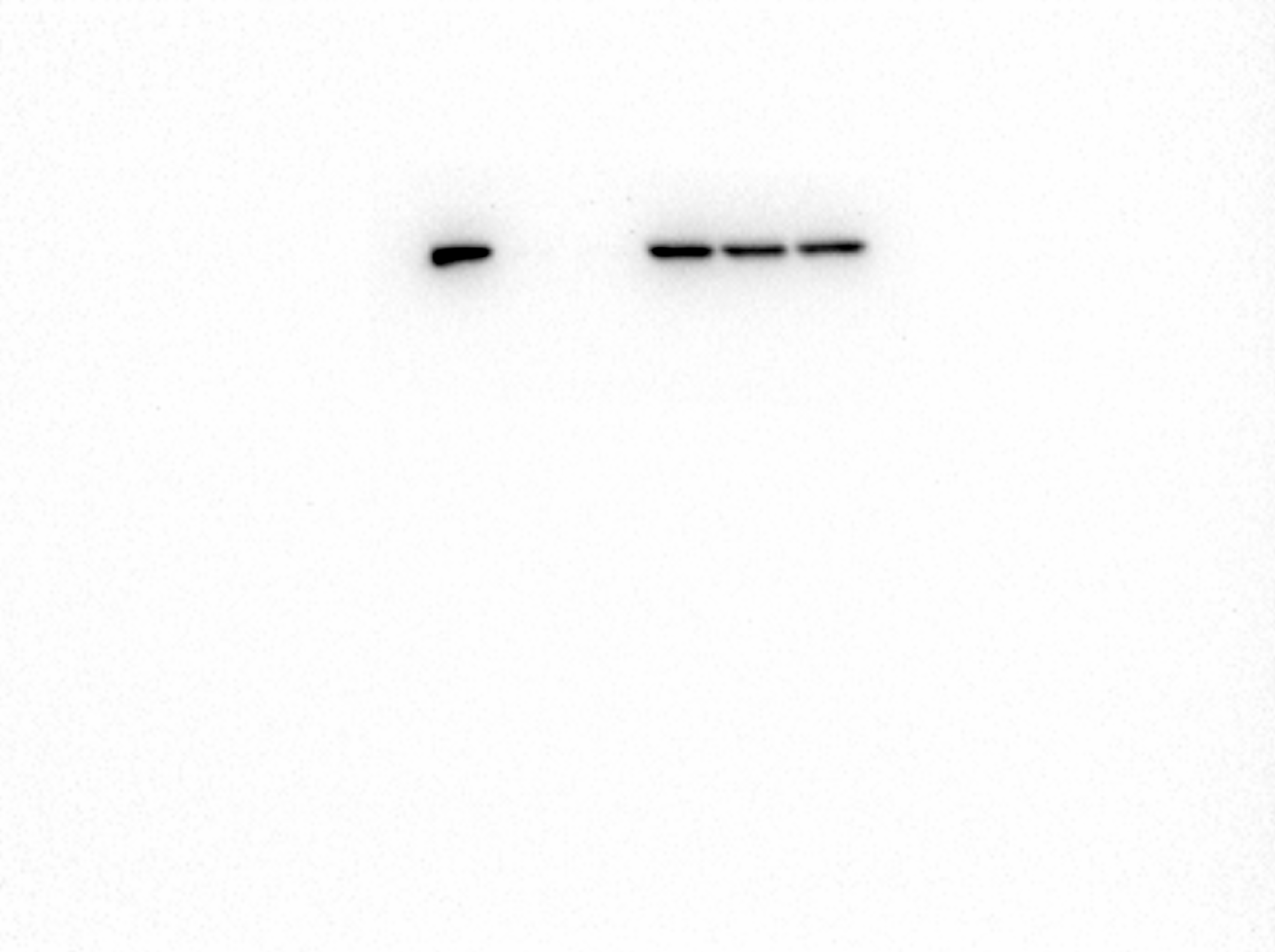

Supplement: Figure 2—source data 2. [file elife-99225-fig2-data2.zip › Figure 2-source data 2/H/prmt1-231-prmt1sg.tif]

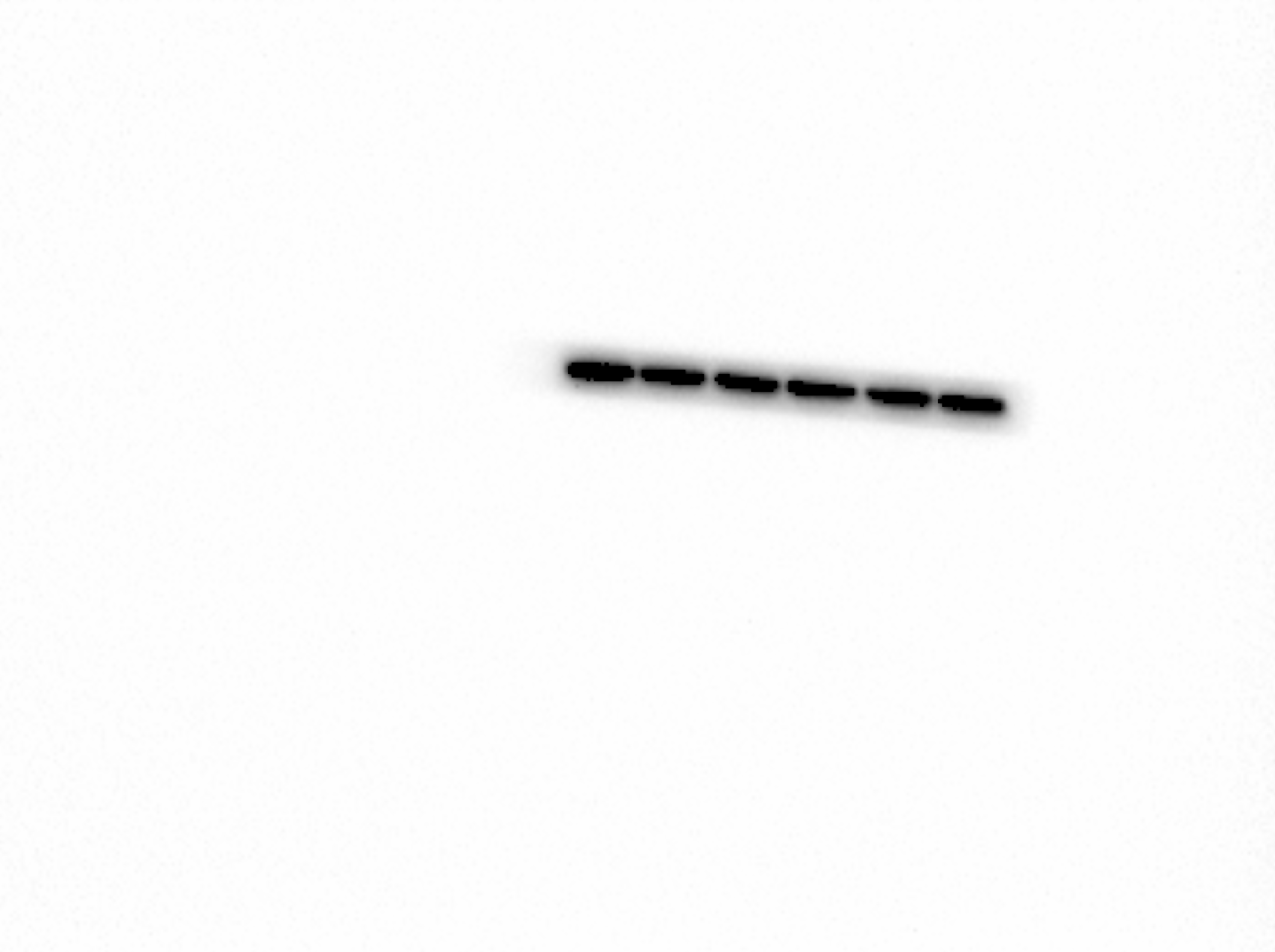

Supplement: Figure 2—source data 2. [file elife-99225-fig2-data2.zip › Figure 2-source data 2/H/tublin-231-prmtsg.tif]

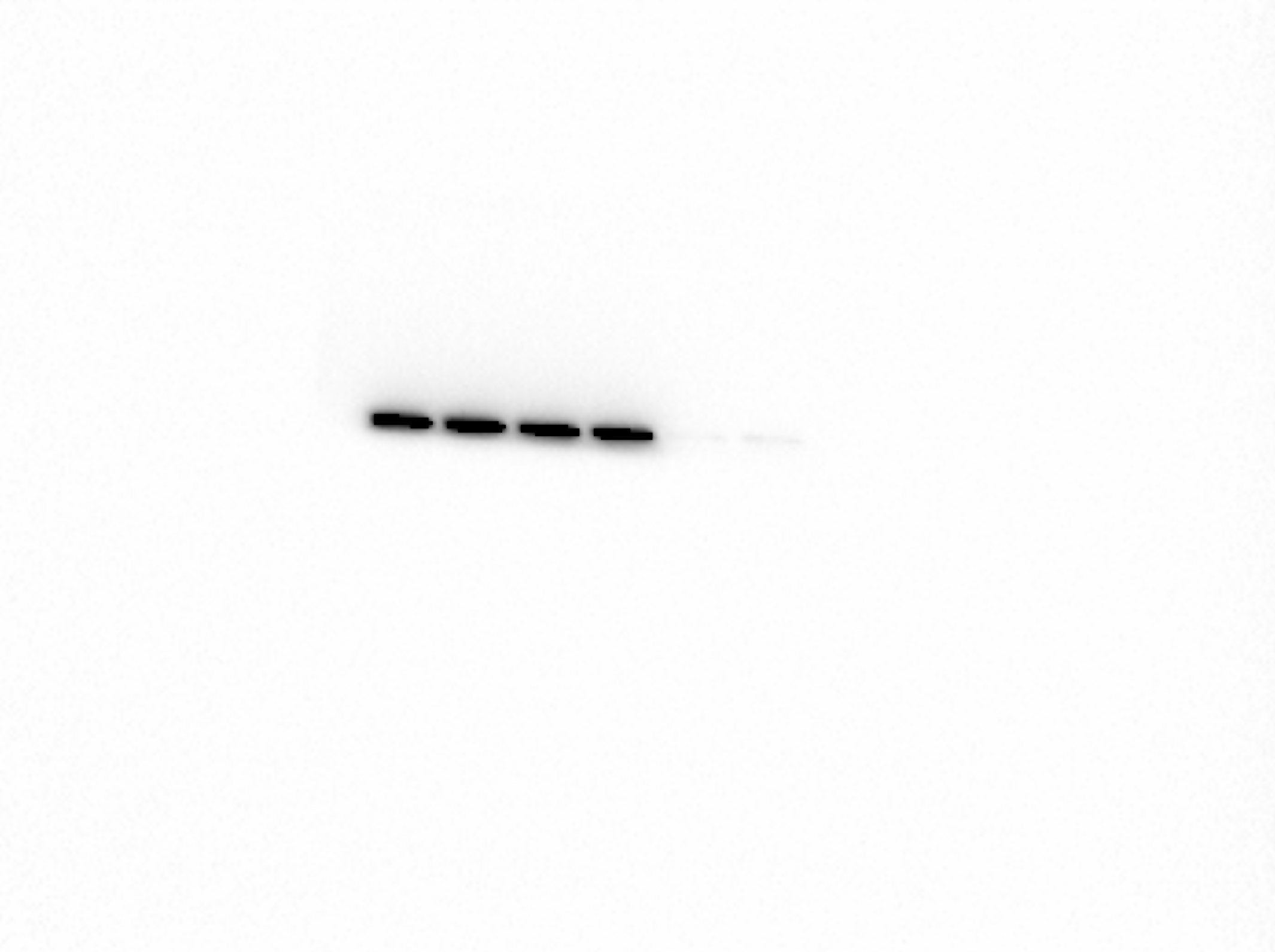

Supplement: Figure 2—source data 2. [file elife-99225-fig2-data2.zip › Figure 2-source data 2/H/prmt5-231-prmt5sg.tif]

Figure 3

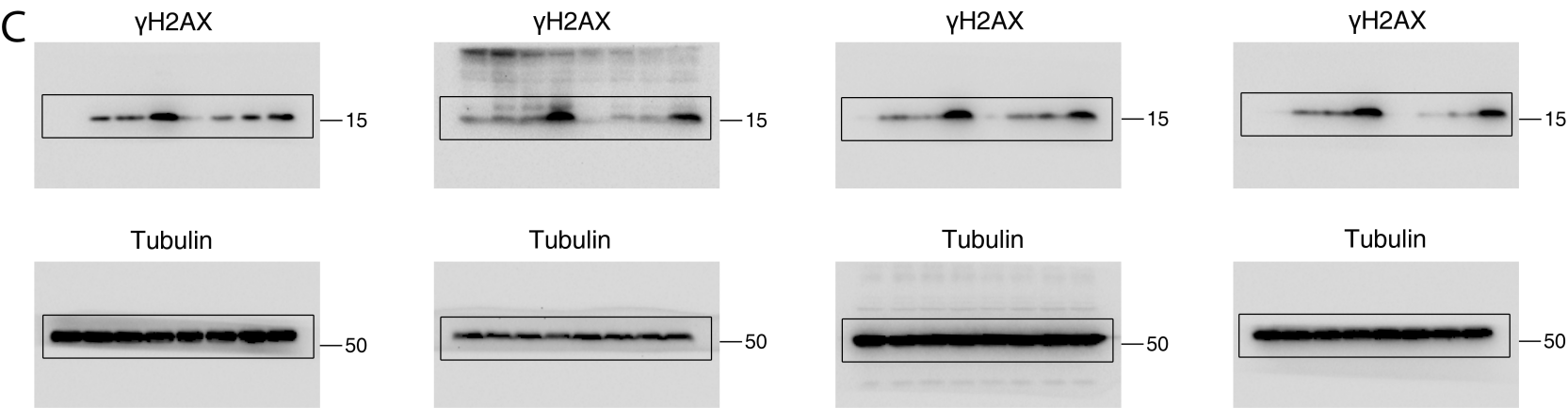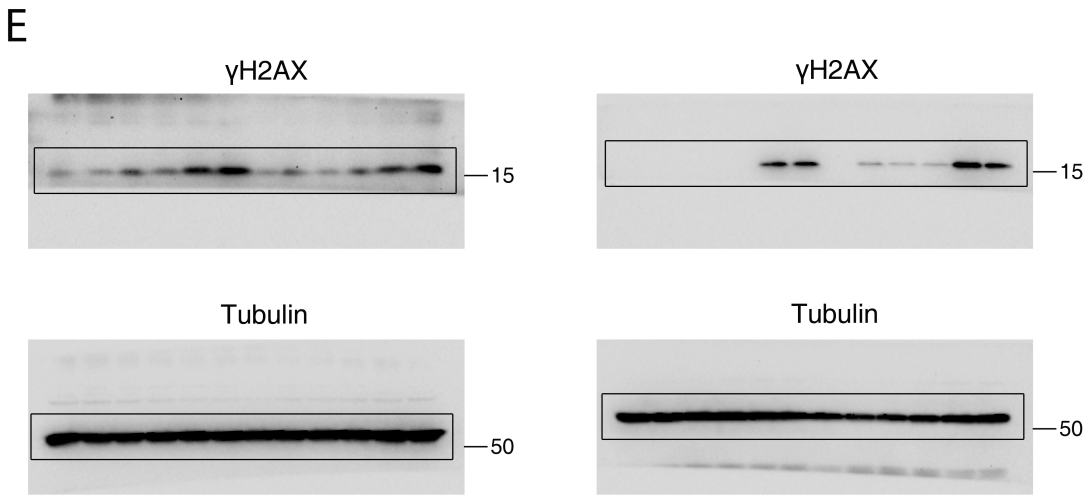

Supplement: Figure 3—source data 1. [file elife-99225-fig3-data1.pdf]

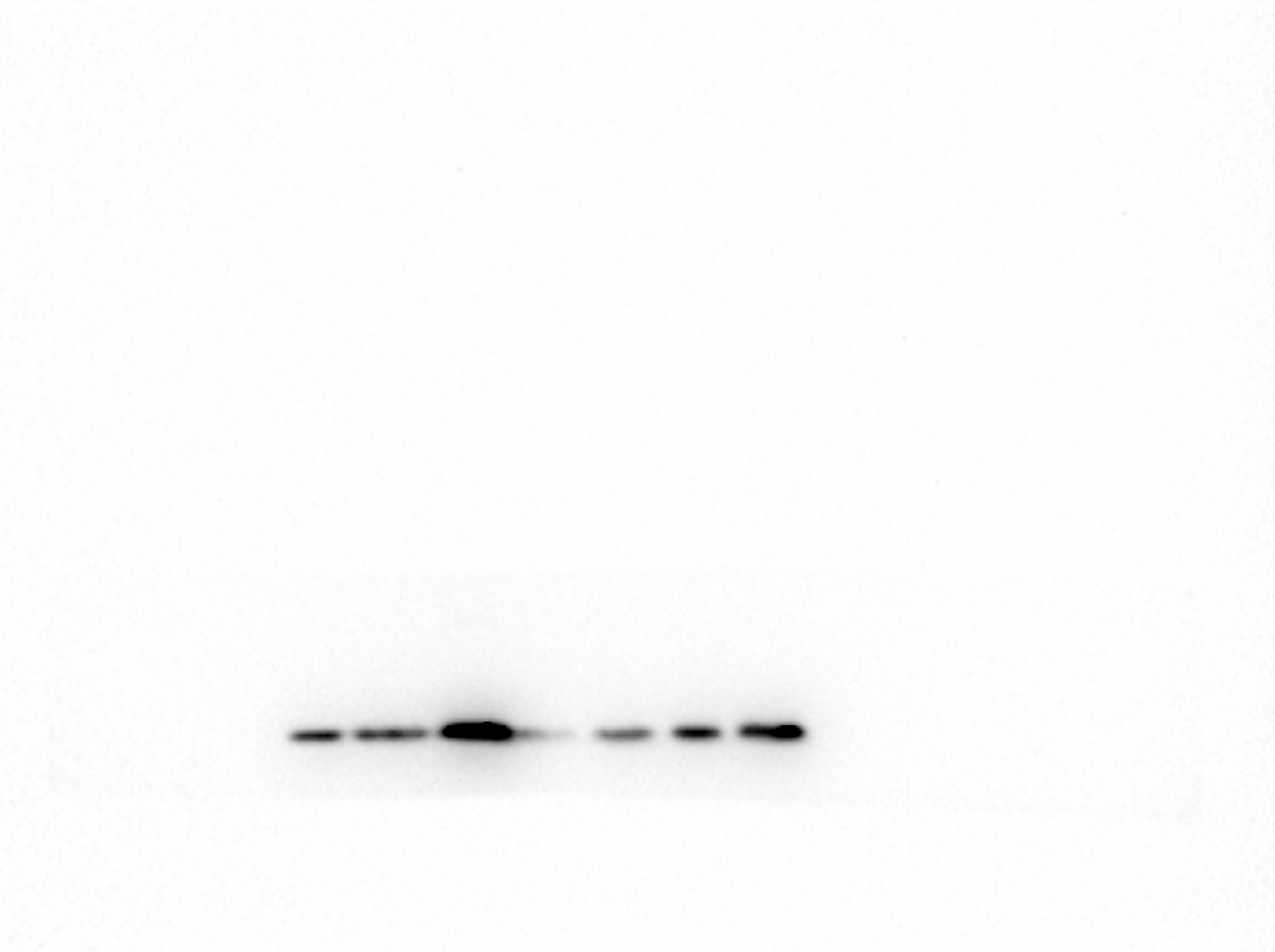

Supplement: Figure 3—source data 2. [file elife-99225-fig3-data2.zip › Figure 3-source data 2/C/OVCAR8/h2a-ov8-combine.tif]

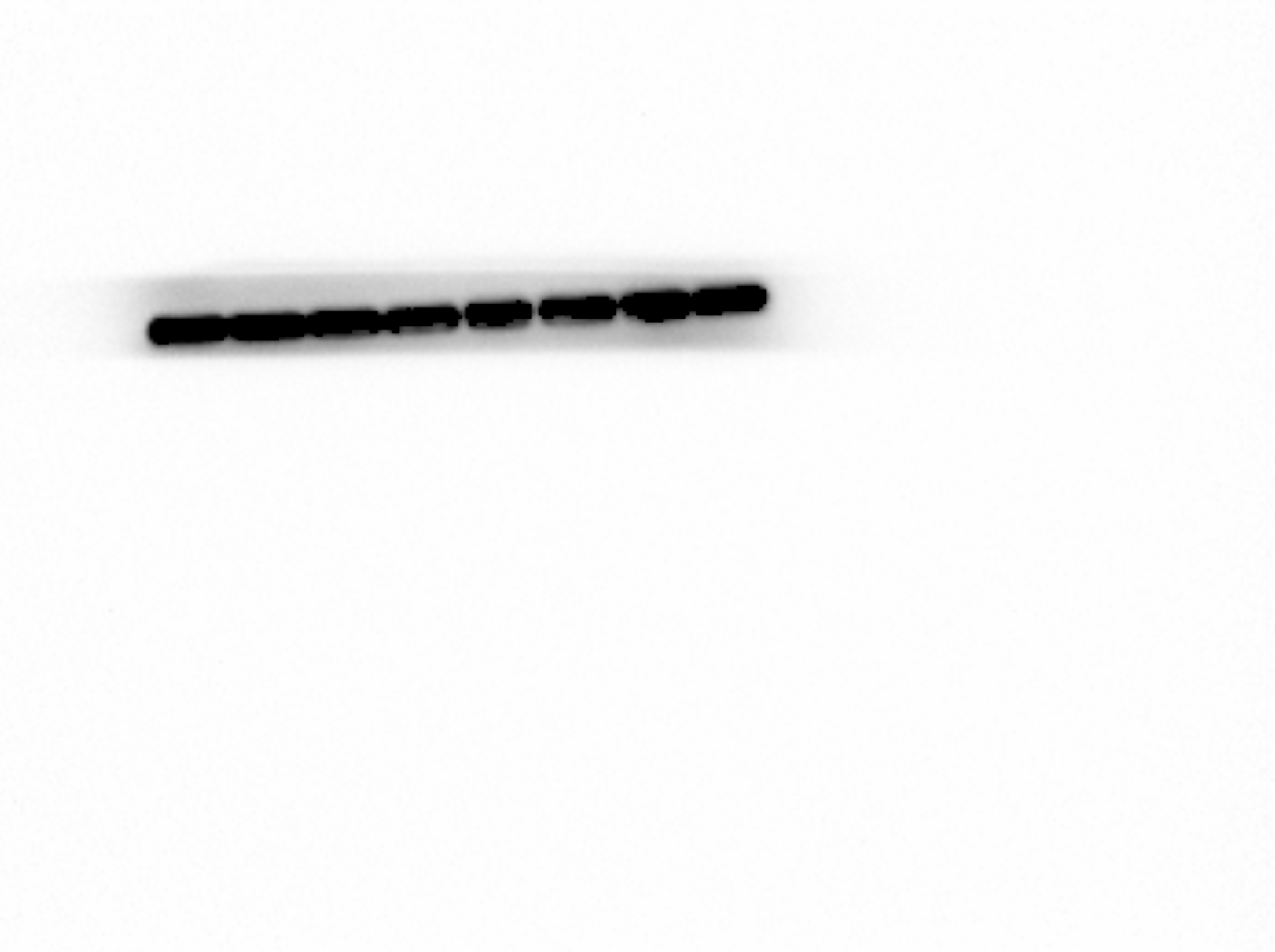

Supplement: Figure 3—source data 2. [file elife-99225-fig3-data2.zip › Figure 3-source data 2/C/OVCAR8/tubulin-ov8-combine.tif]

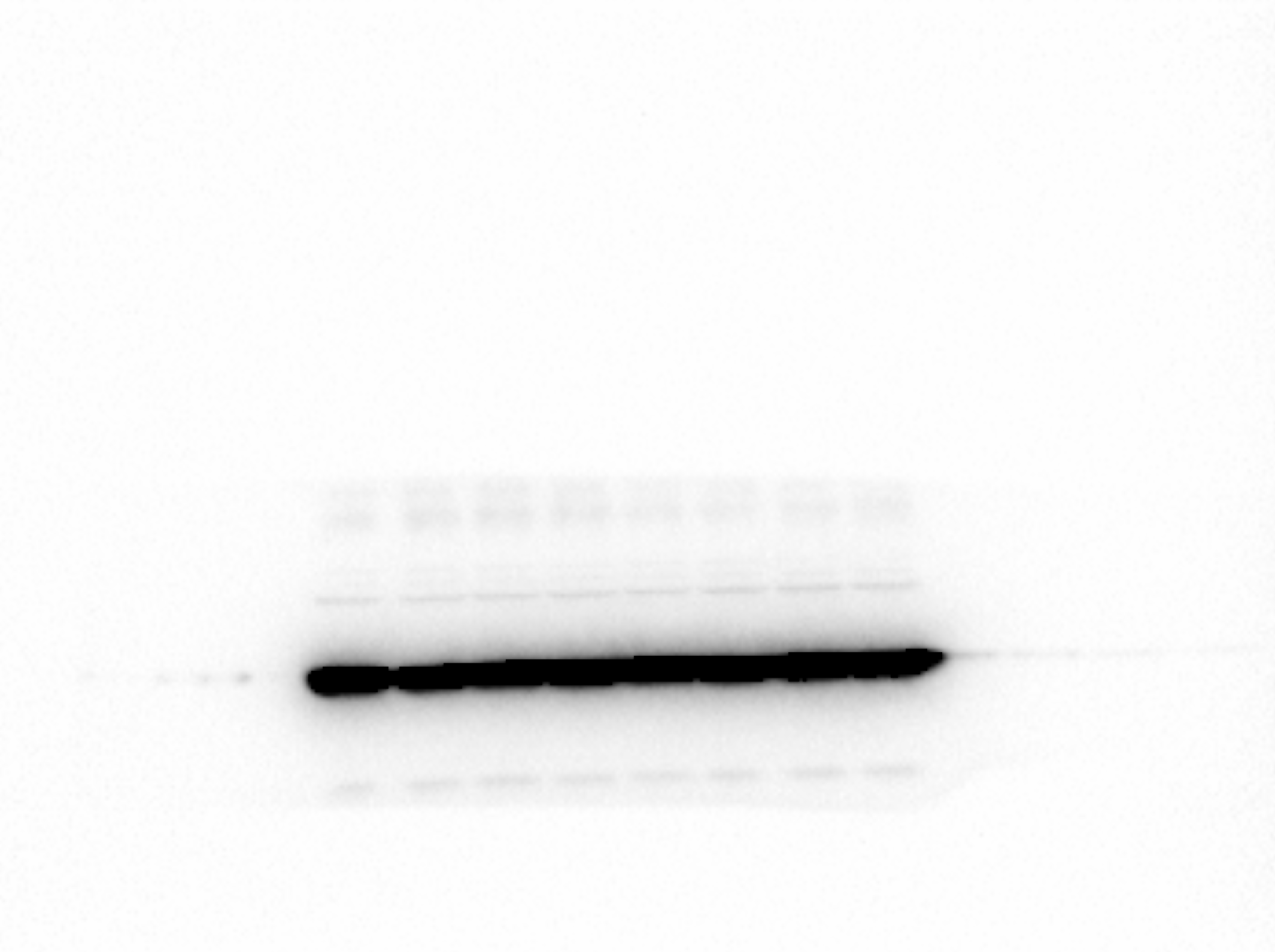

Supplement: Figure 3—source data 2. [file elife-99225-fig3-data2.zip › Figure 3-source data 2/C/MDA-MB-231/tubulin-231-combine.tif]

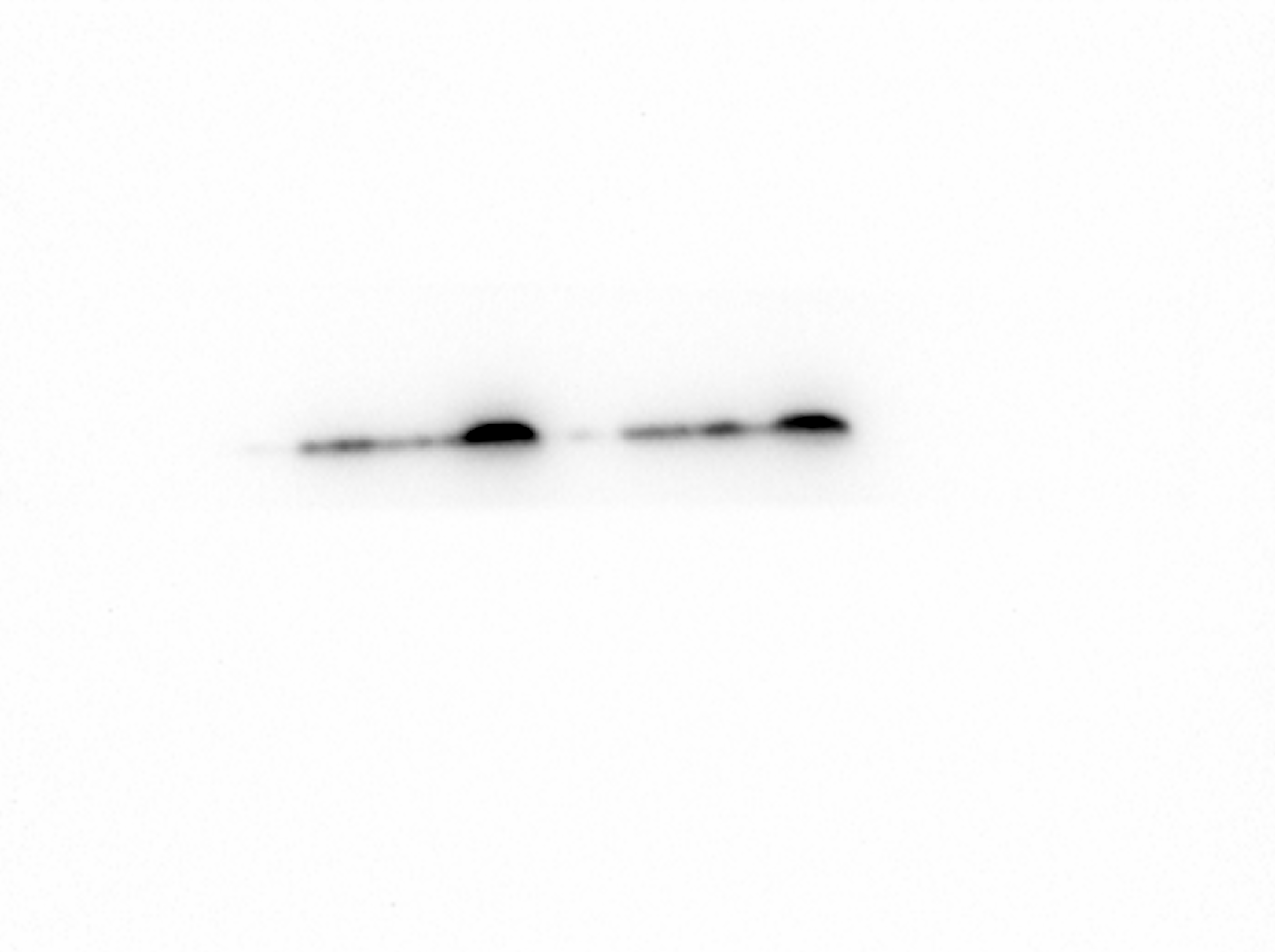

Supplement: Figure 3—source data 2. [file elife-99225-fig3-data2.zip › Figure 3-source data 2/C/MDA-MB-231/h2a-231-combine.tif]

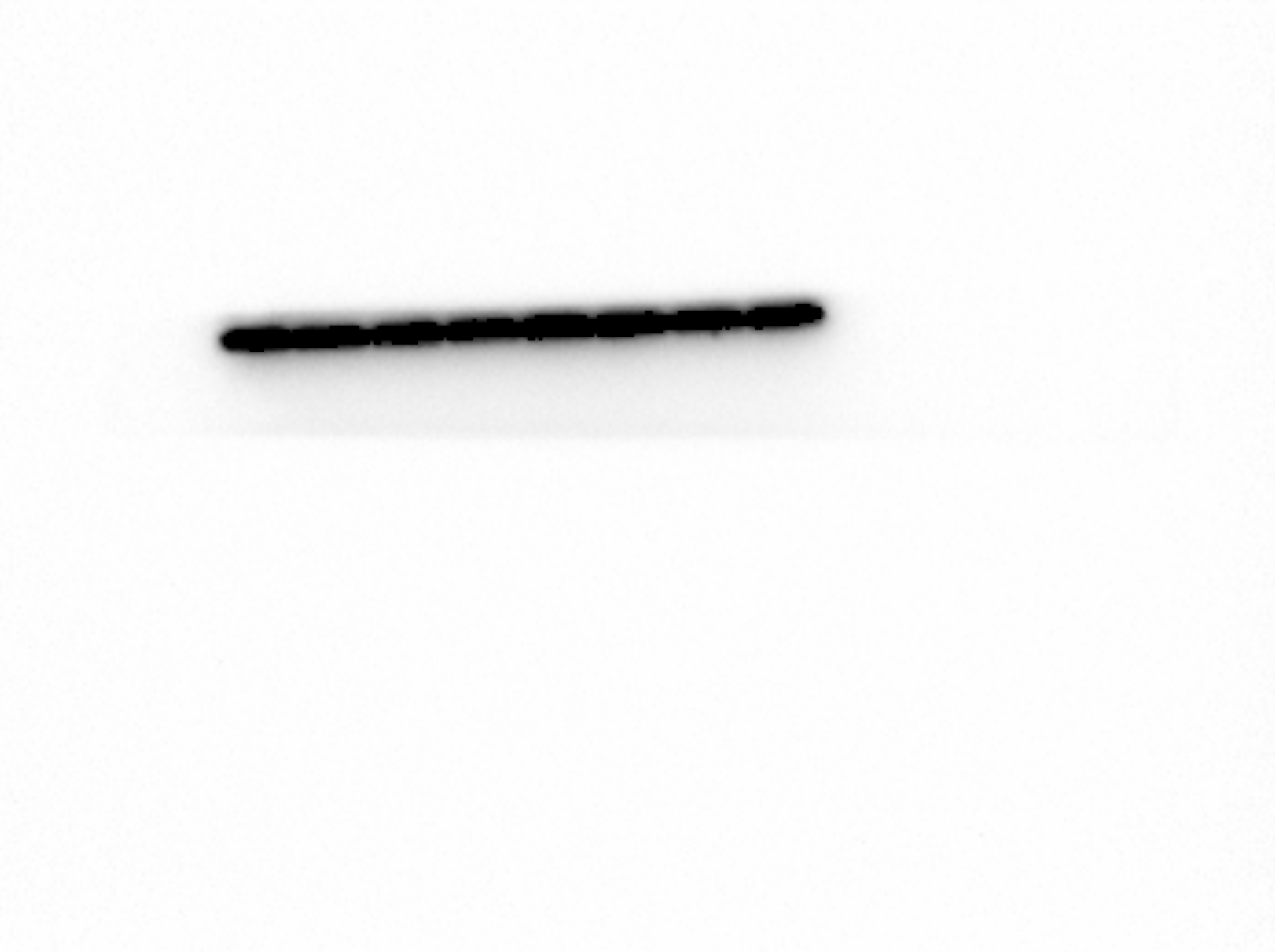

Supplement: Figure 3—source data 2. [file elife-99225-fig3-data2.zip › Figure 3-source data 2/C/MDA-MB-468/tubulin-468-combine.tif]

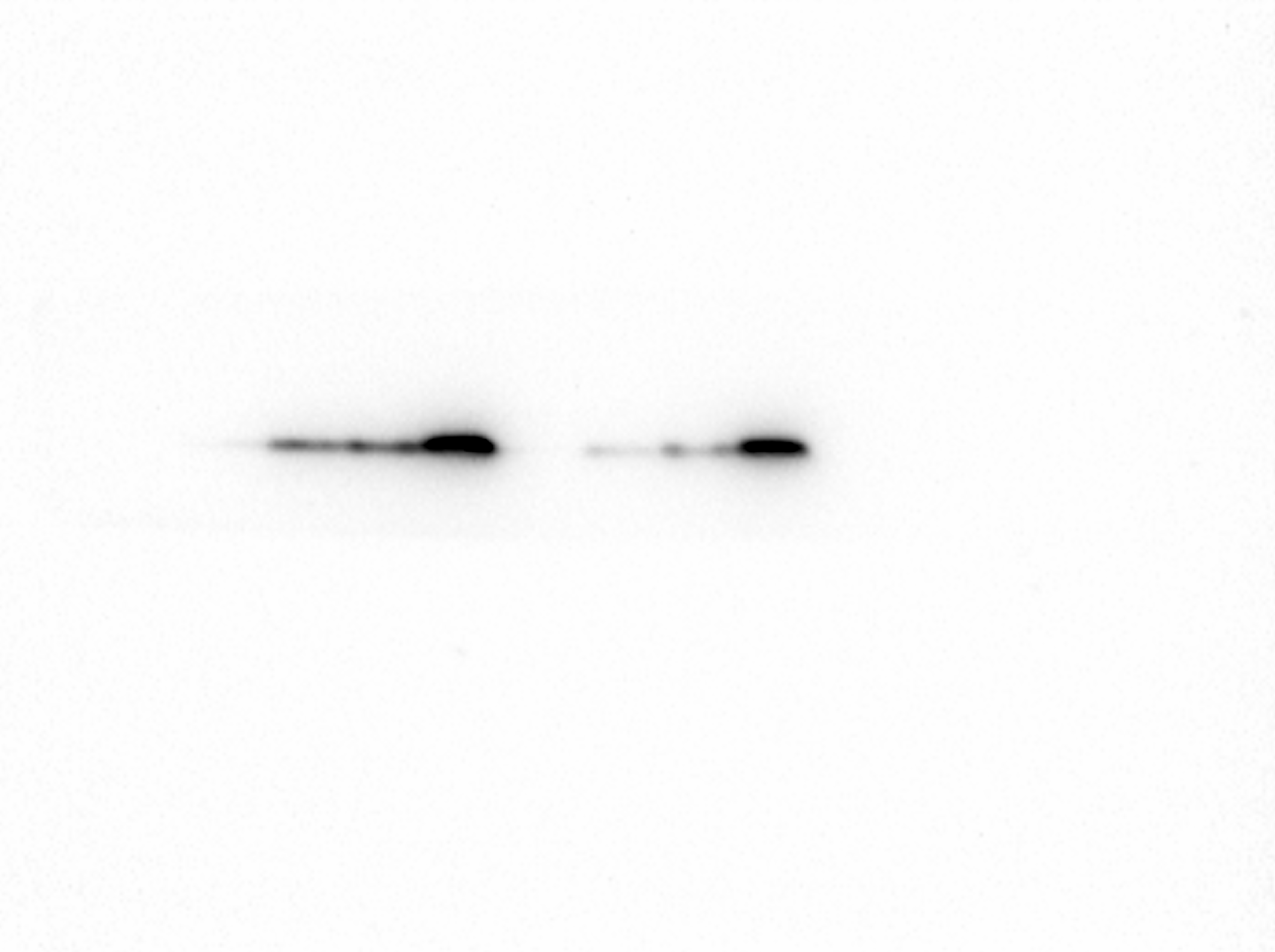

Supplement: Figure 3—source data 2. [file elife-99225-fig3-data2.zip › Figure 3-source data 2/C/MDA-MB-468/h2a-468-combine.tif]

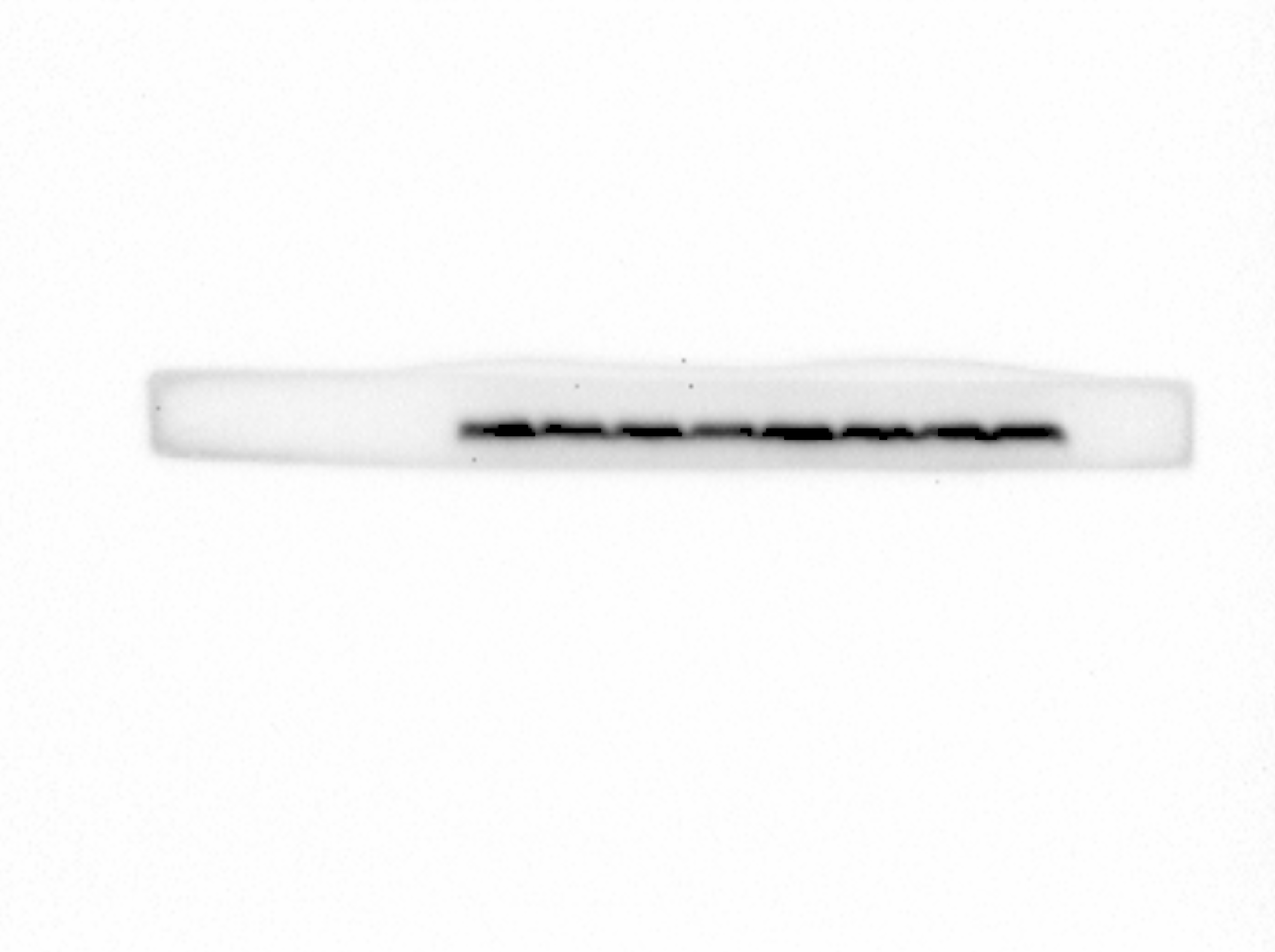

Supplement: Figure 3—source data 2. [file elife-99225-fig3-data2.zip › Figure 3-source data 2/C/OVCAR3/tubulin-ov3-combine.tif]

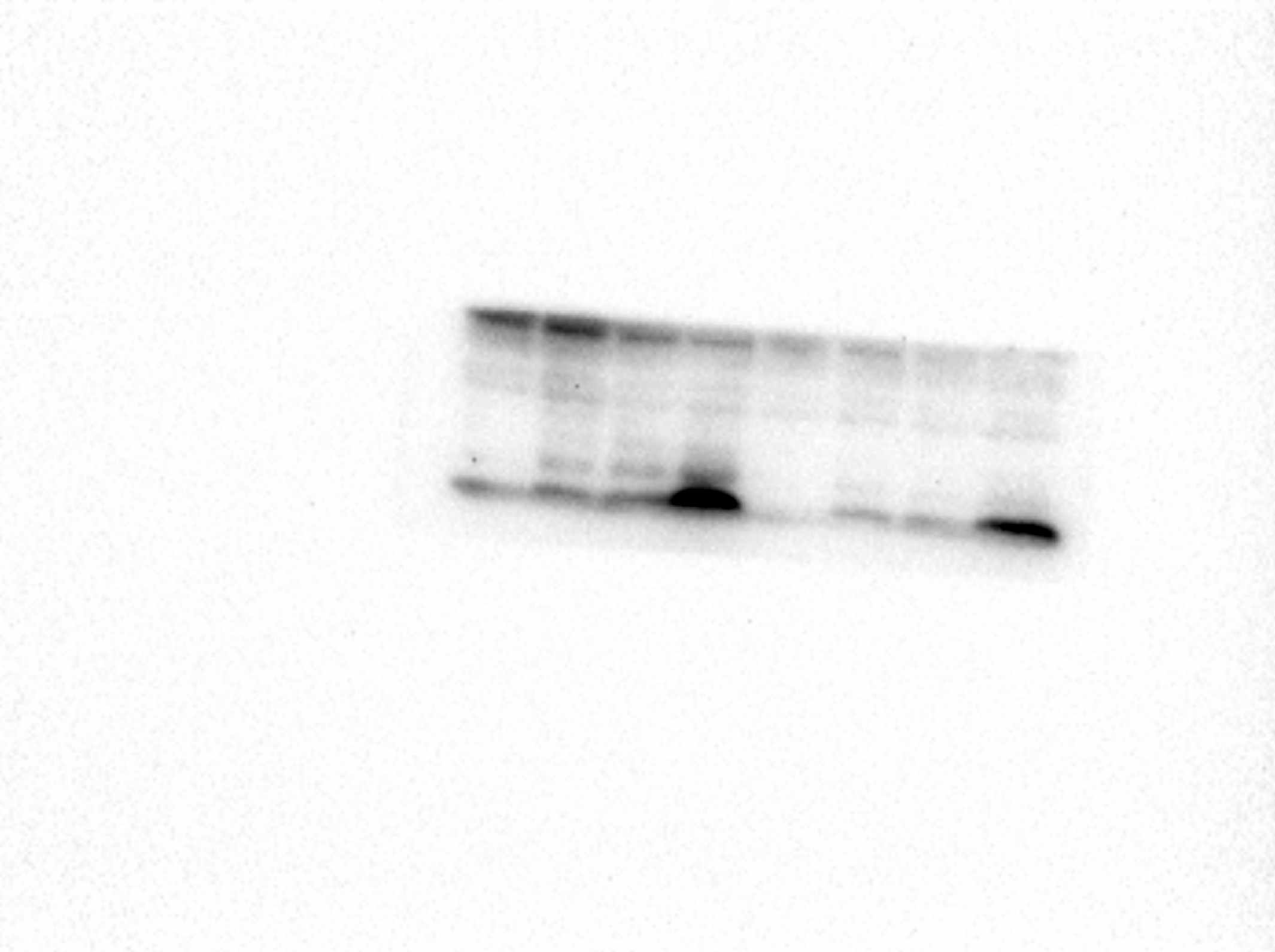

Supplement: Figure 3—source data 2. [file elife-99225-fig3-data2.zip › Figure 3-source data 2/C/OVCAR3/h2a-ov3-combine.tif]

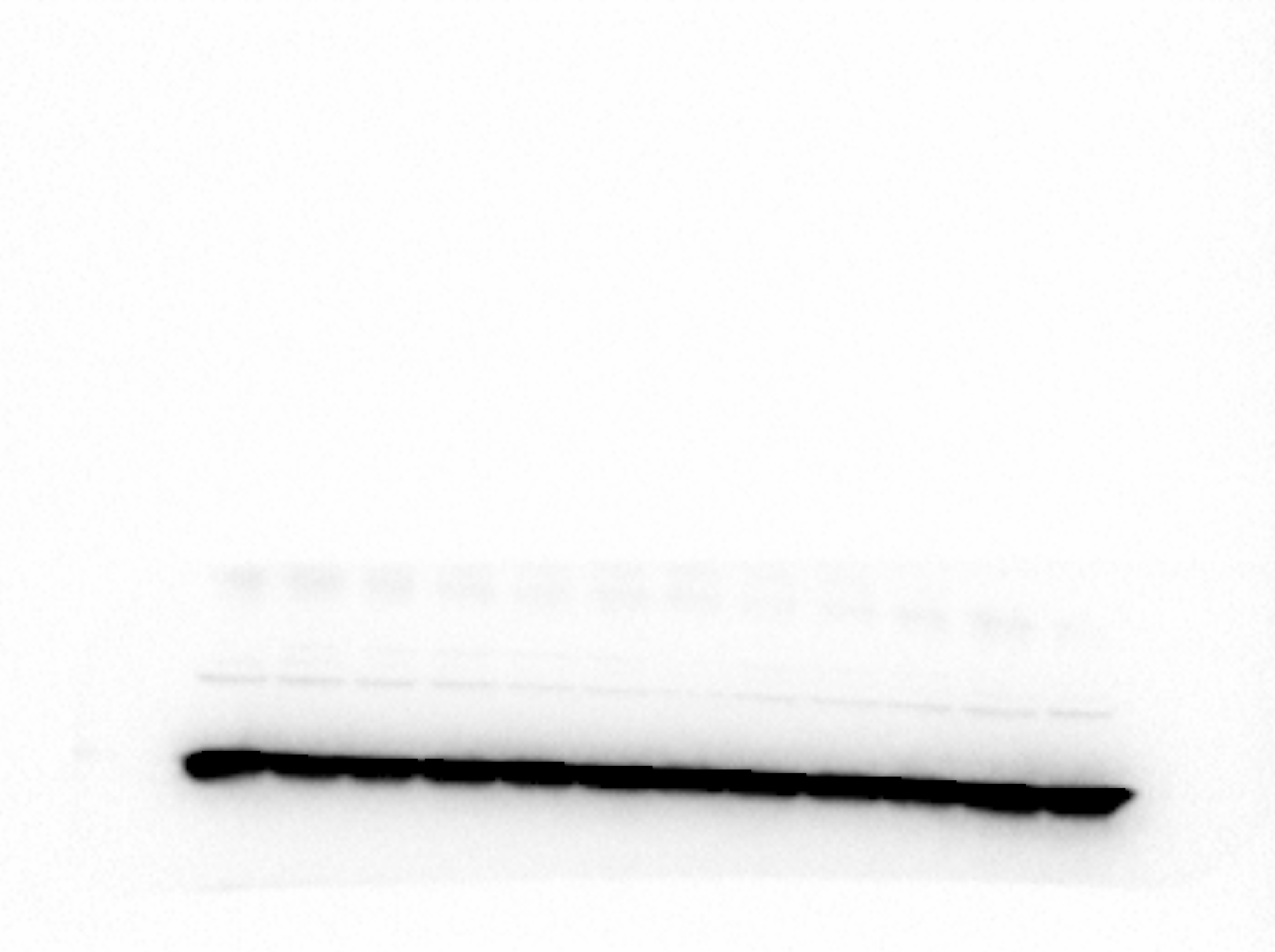

Supplement: Figure 3—source data 2. [file elife-99225-fig3-data2.zip › Figure 3-source data 2/E/OVCAR8/tubulin-ov8-olasg.tif]

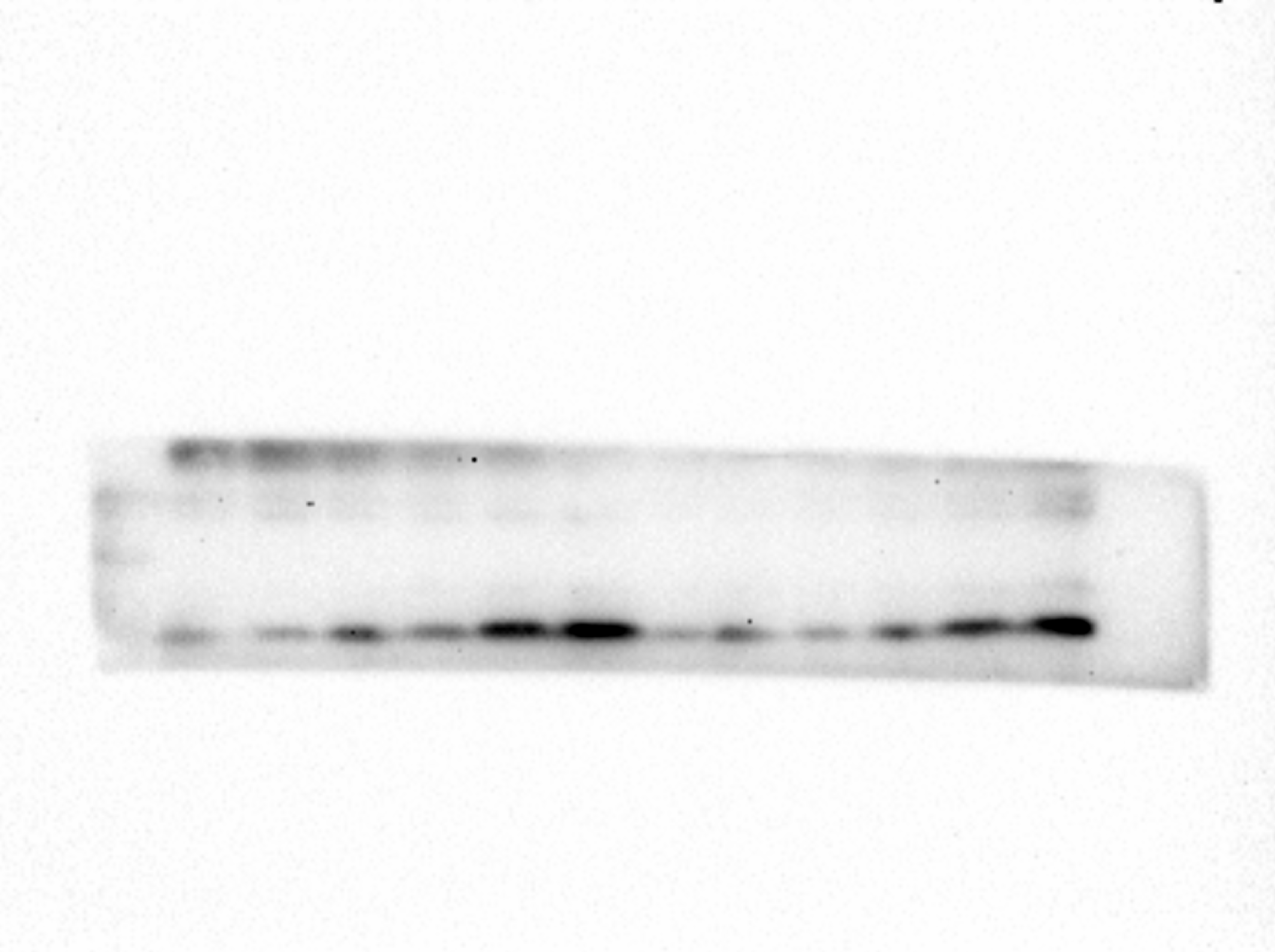

Supplement: Figure 3—source data 2. [file elife-99225-fig3-data2.zip › Figure 3-source data 2/E/OVCAR8/h2a-ov8-olasg.tif]

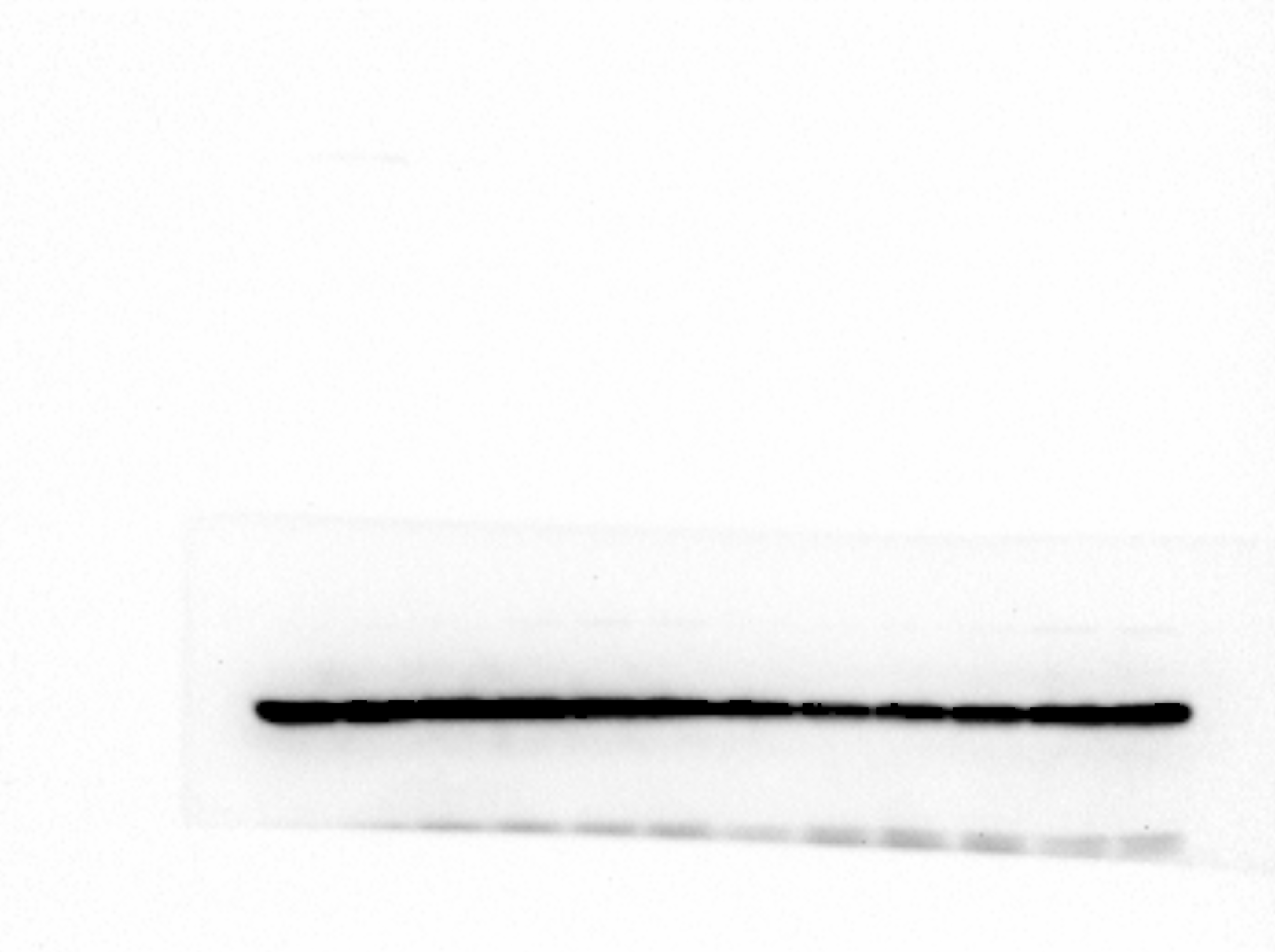

Supplement: Figure 3—source data 2. [file elife-99225-fig3-data2.zip › Figure 3-source data 2/E/MDA-MB-231/tubulin-231-olasg.tif]

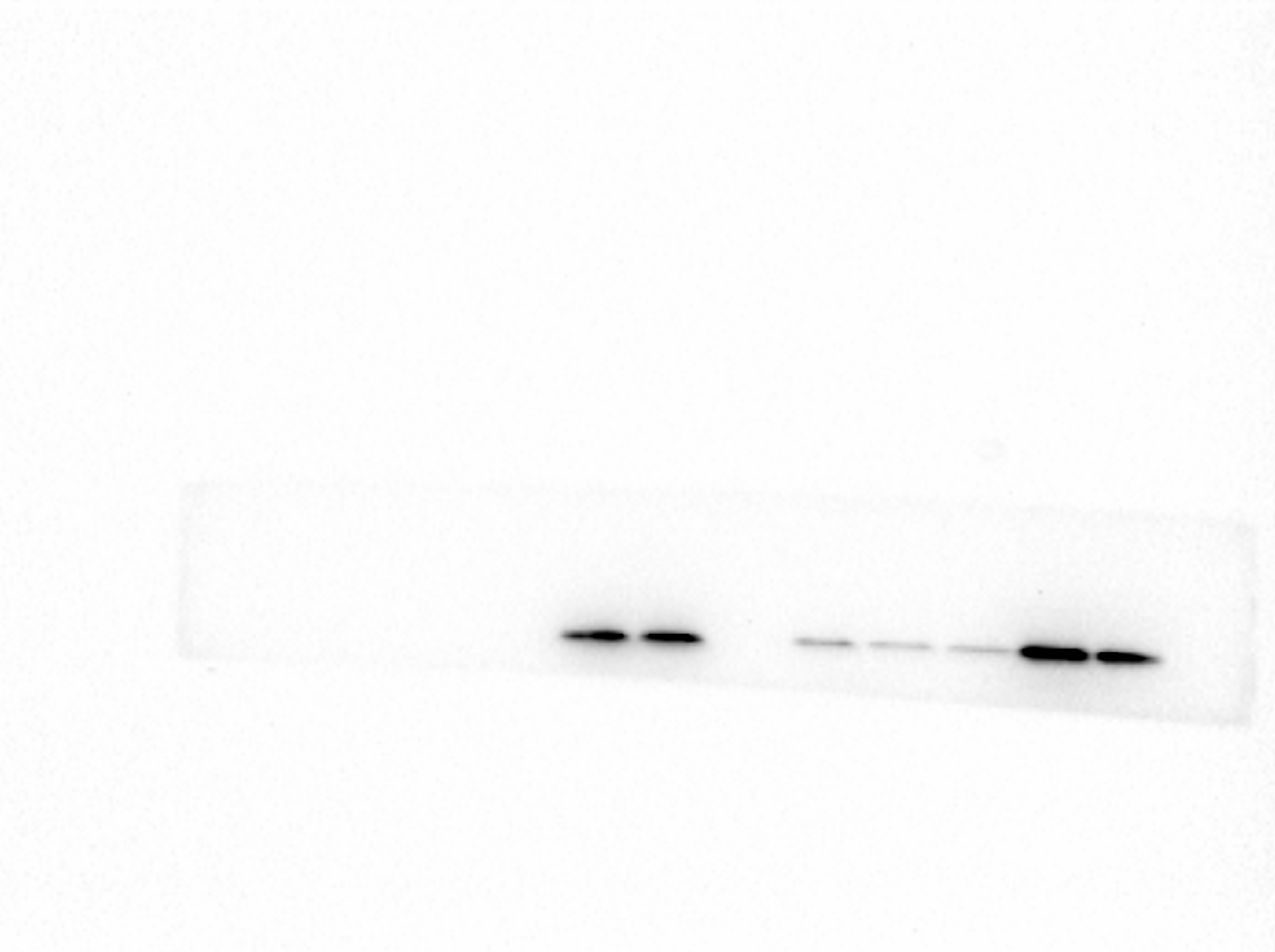

Supplement: Figure 3—source data 2. [file elife-99225-fig3-data2.zip › Figure 3-source data 2/E/MDA-MB-231/h2a-231-olasg.tif]

Figure 5

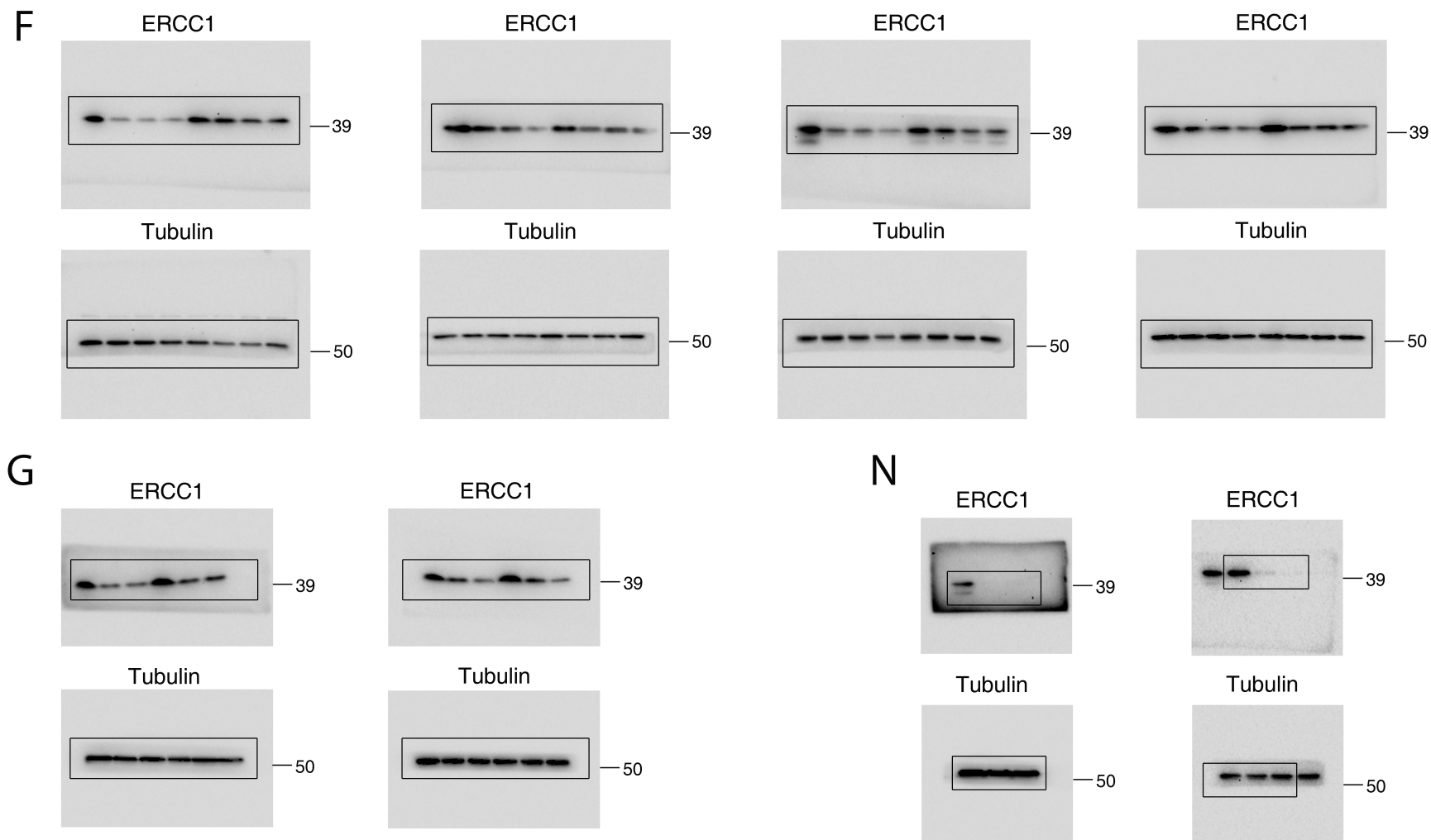

Supplement: Figure 5—source data 1. [file elife-99225-fig5-data1.pdf]

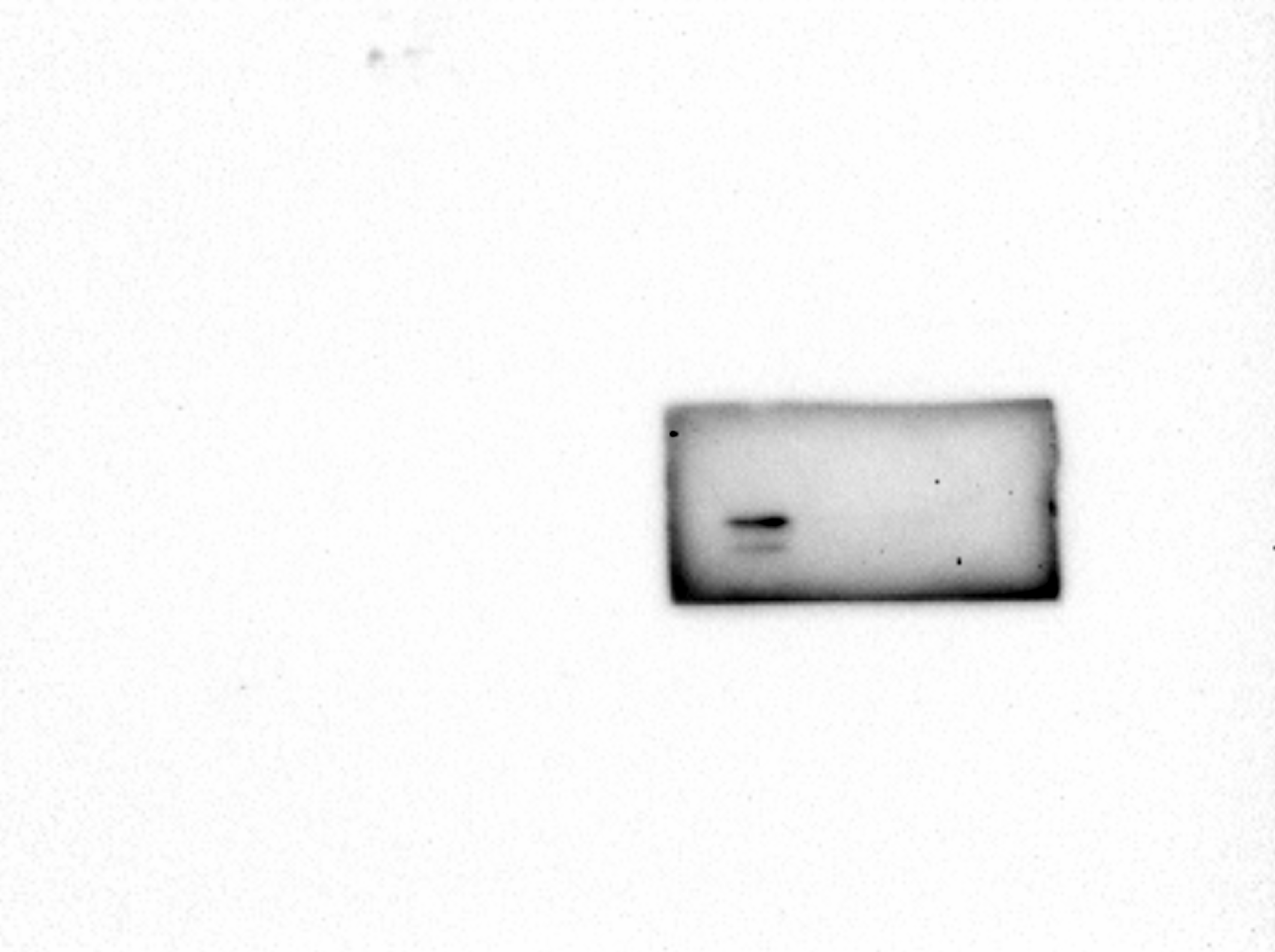

Supplement: Figure 5—source data 2. [file elife-99225-fig5-data2.zip › Figure 5-source data 2/N/OVCAR8/ercc1-ov8-ercc1sg.tif]

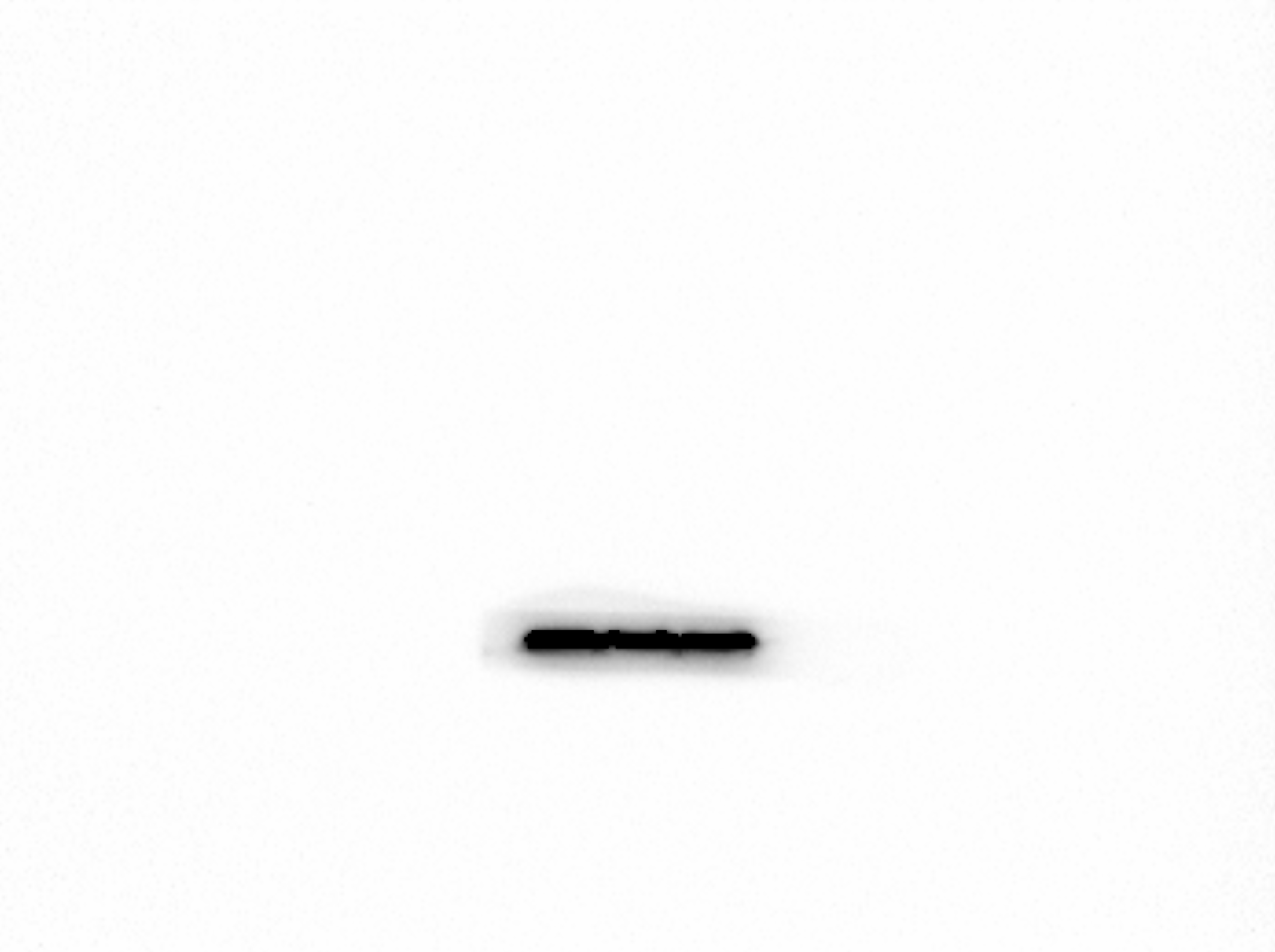

Supplement: Figure 5—source data 2. [file elife-99225-fig5-data2.zip › Figure 5-source data 2/N/OVCAR8/tubulin-ov8-ercc1sg.tif]

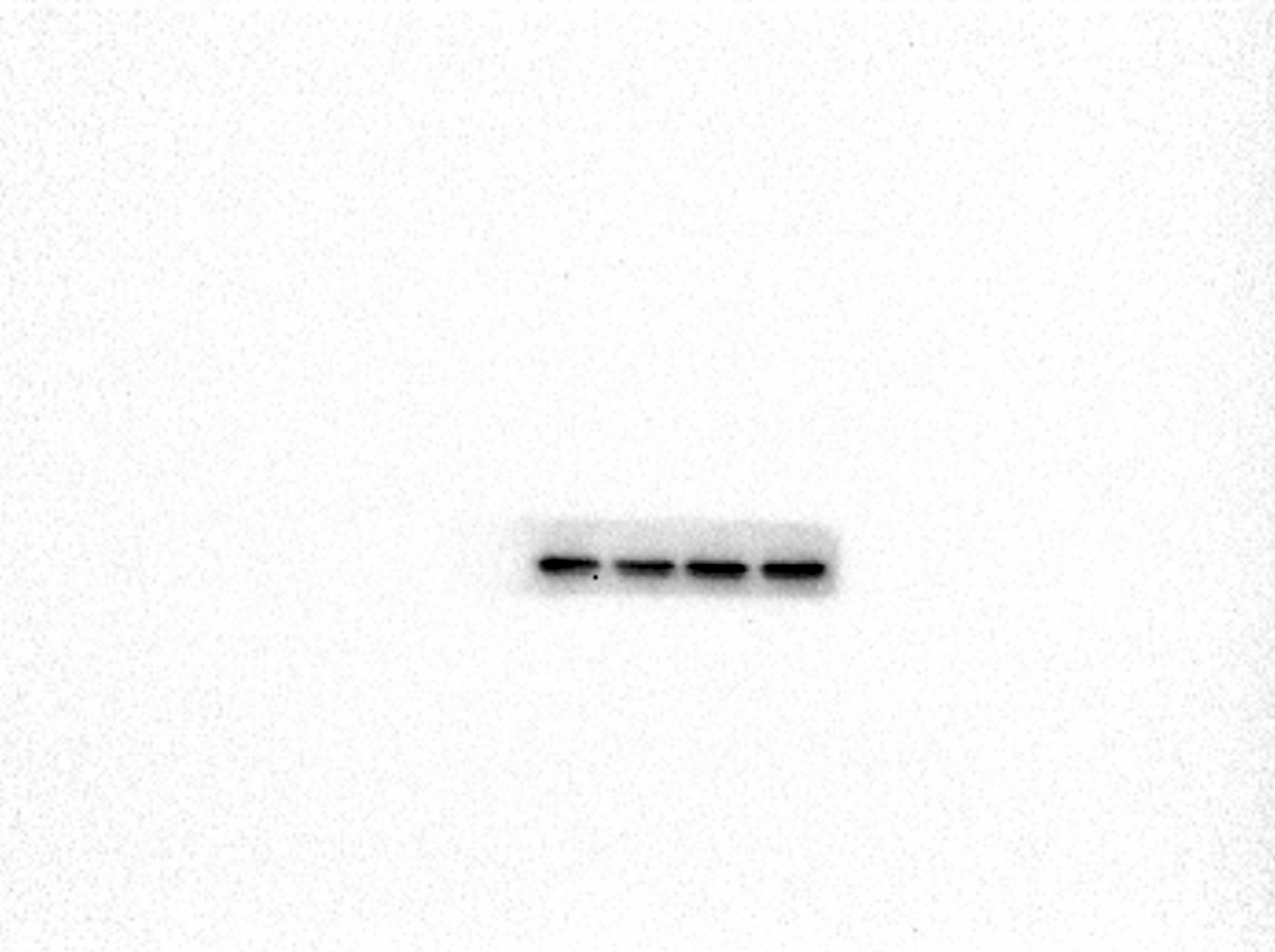

Supplement: Figure 5—source data 2. [file elife-99225-fig5-data2.zip › Figure 5-source data 2/N/MDA-MB-231/tubulin-231-ercc1sg.tif]

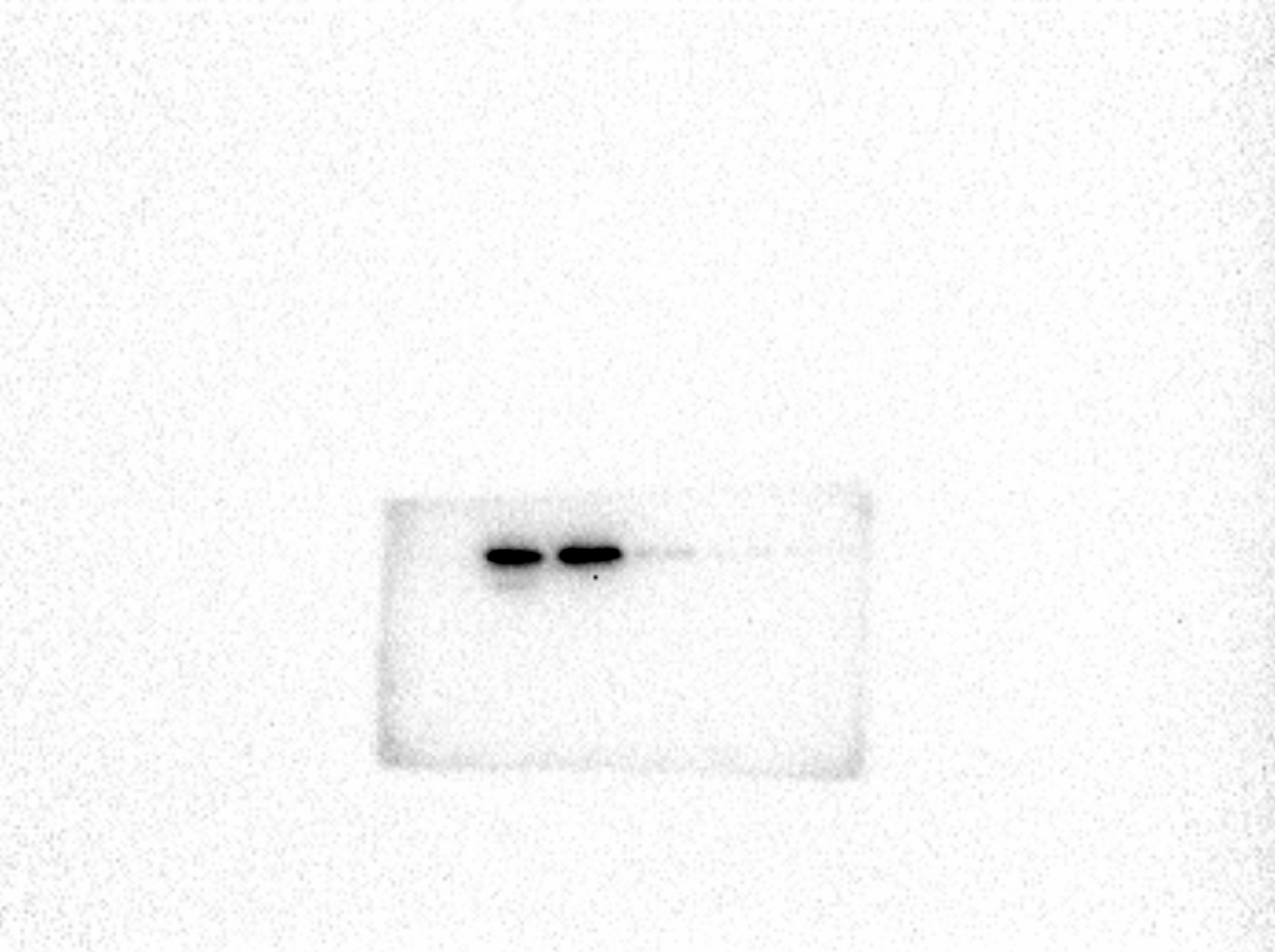

Supplement: Figure 5—source data 2. [file elife-99225-fig5-data2.zip › Figure 5-source data 2/N/MDA-MB-231/ercc1-231-ercc1sg.tif]

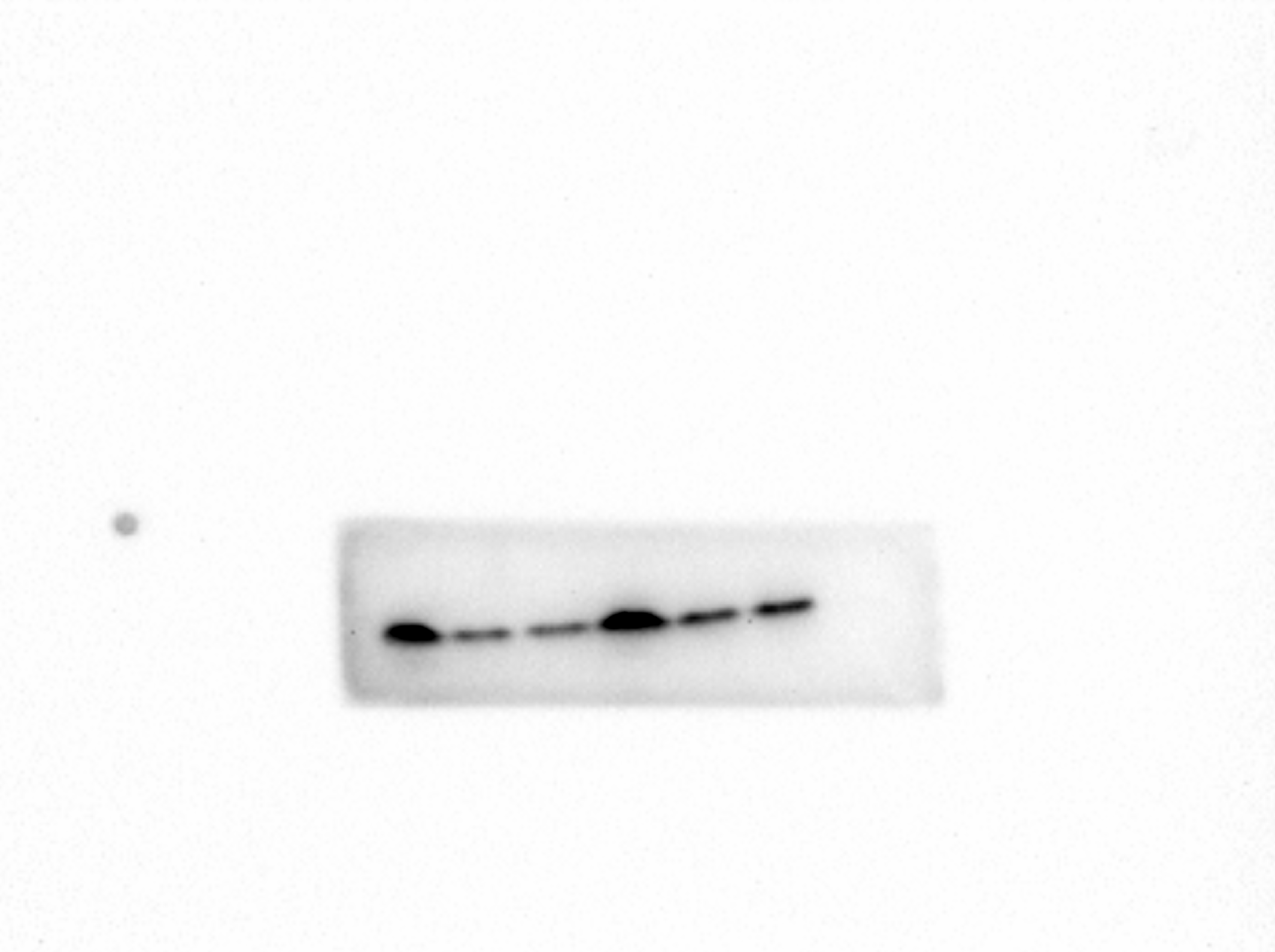

Supplement: Figure 5—source data 2. [file elife-99225-fig5-data2.zip › Figure 5-source data 2/G/OVCAR8/ercc1-ov8-prmtsg.tif]

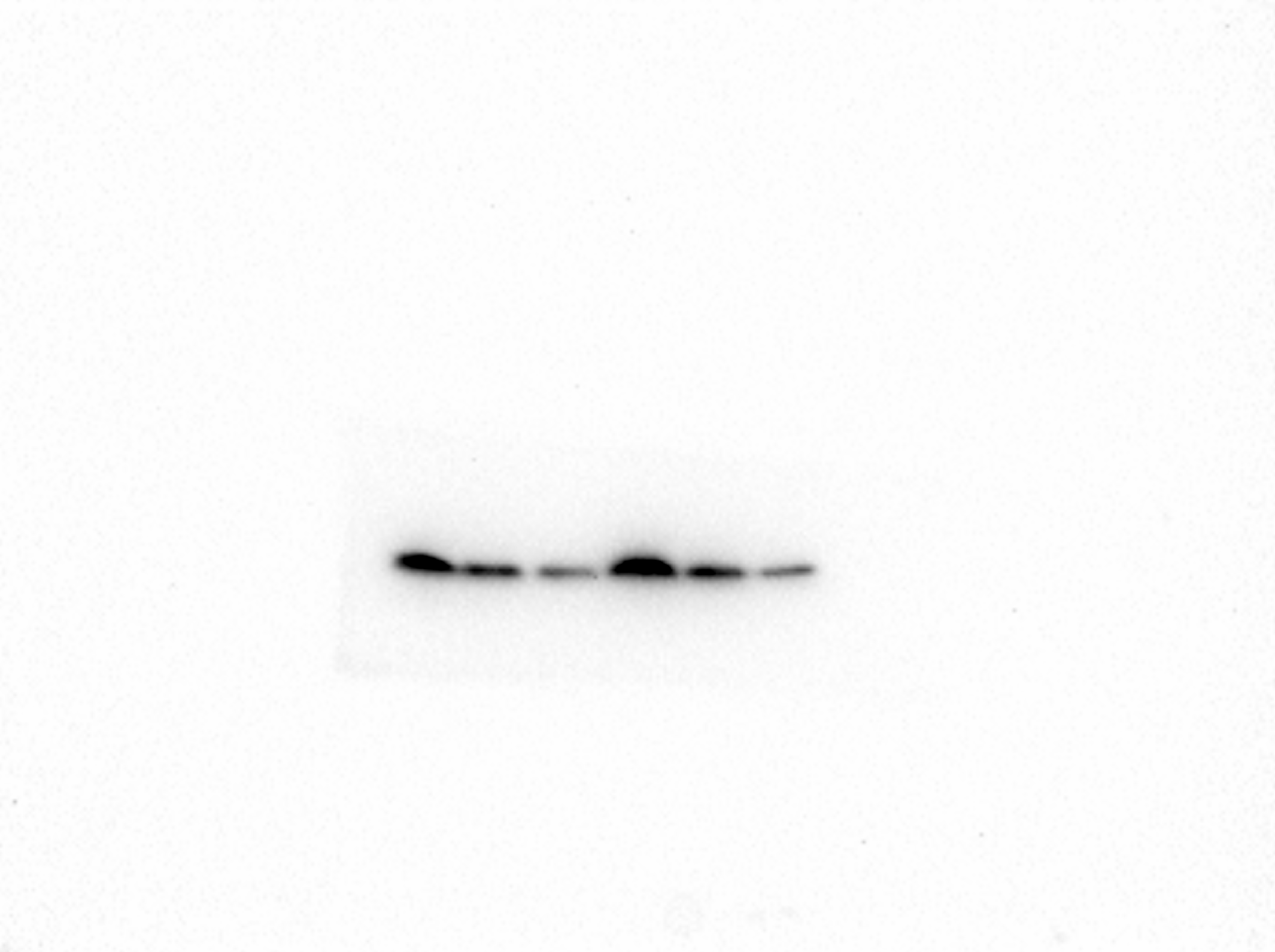

Supplement: Figure 5—source data 2. [file elife-99225-fig5-data2.zip › Figure 5-source data 2/G/MDA-MB-231/ercc1-231-prmtsg.tif]

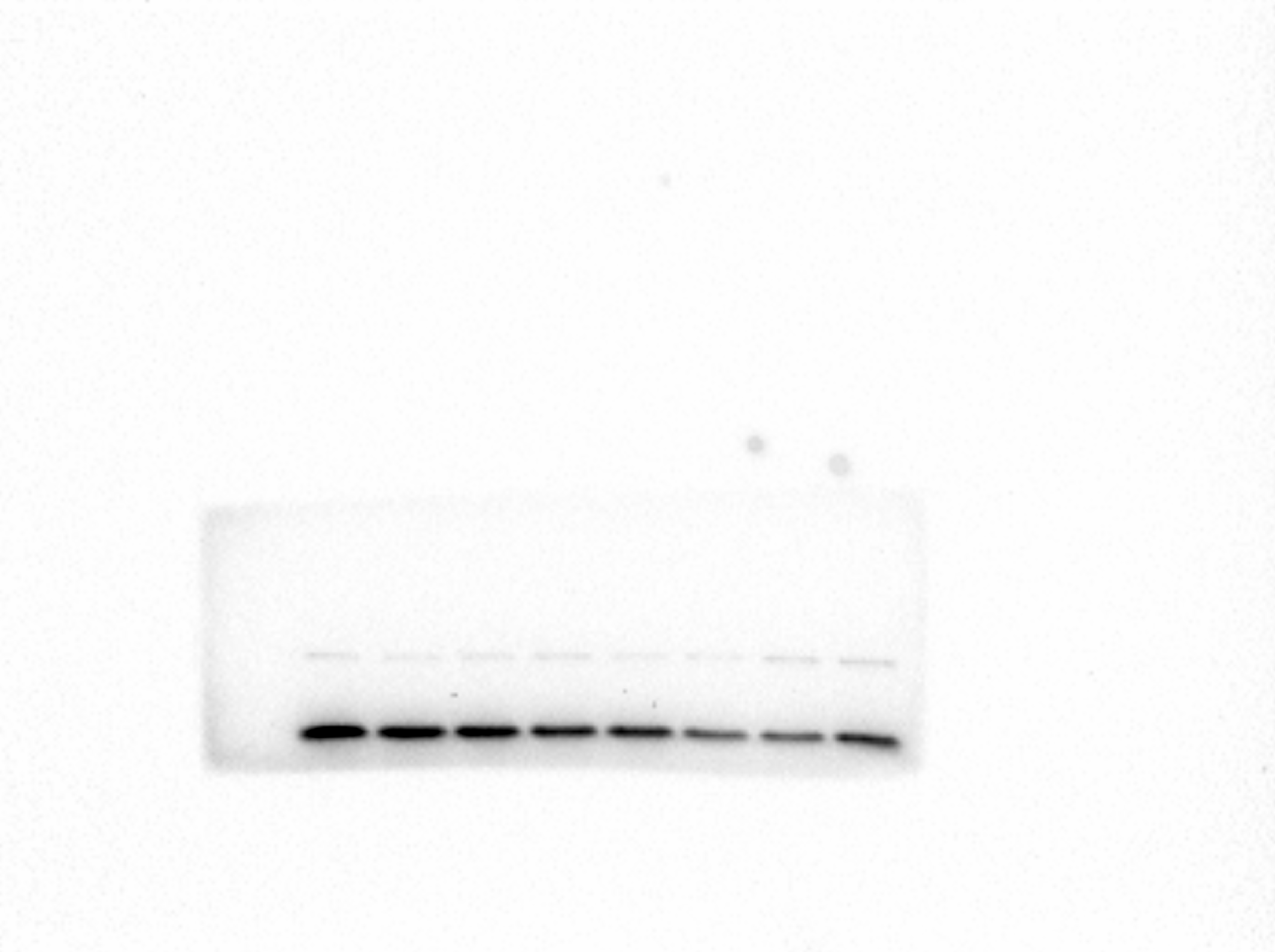

Supplement: Figure 5—source data 2. [file elife-99225-fig5-data2.zip › Figure 5-source data 2/F/OVCAR8/tubulin-ov8-dose.tif]

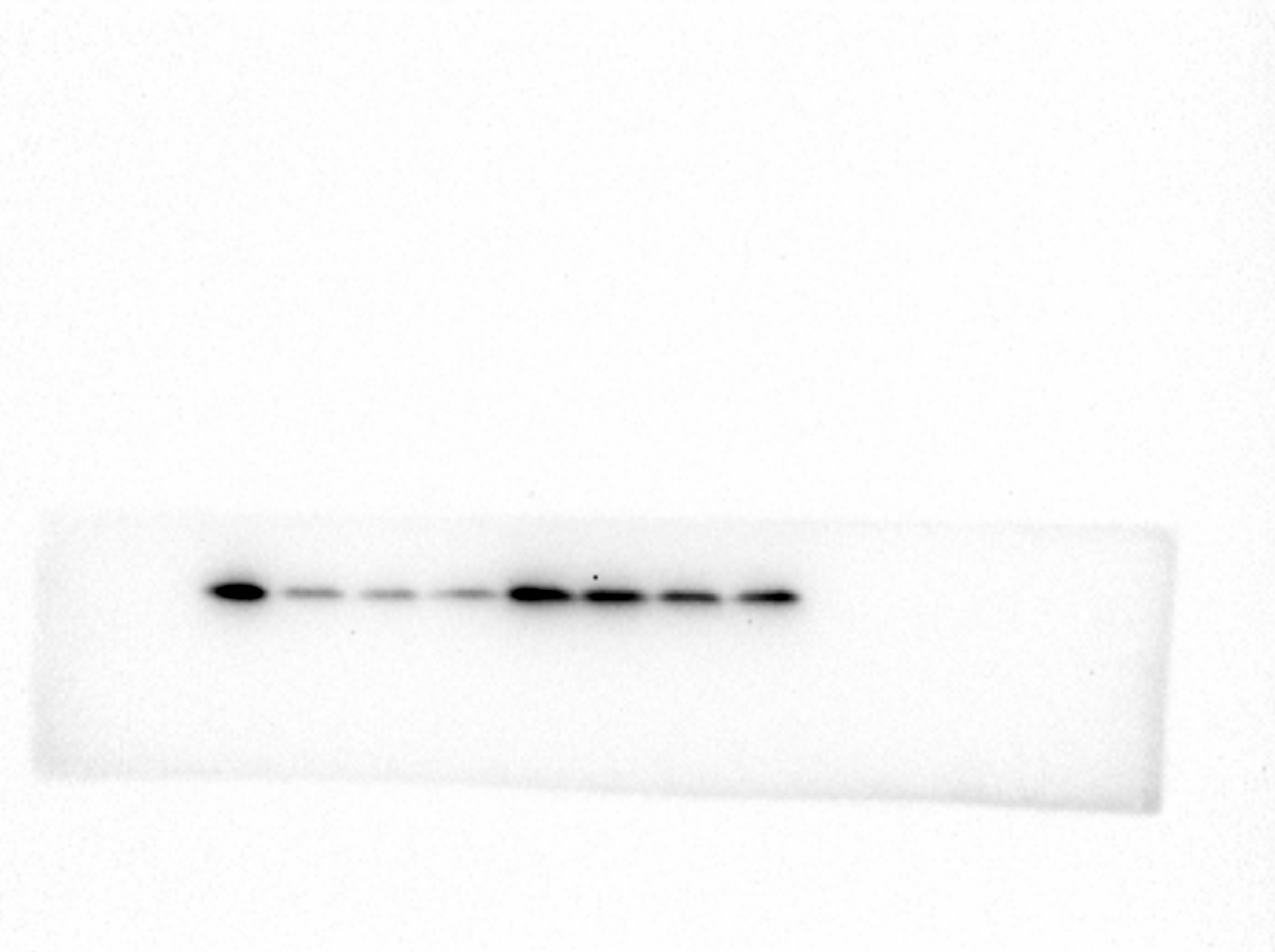

Supplement: Figure 5—source data 2. [file elife-99225-fig5-data2.zip › Figure 5-source data 2/F/OVCAR8/ercc1-ov8-dose.tif]

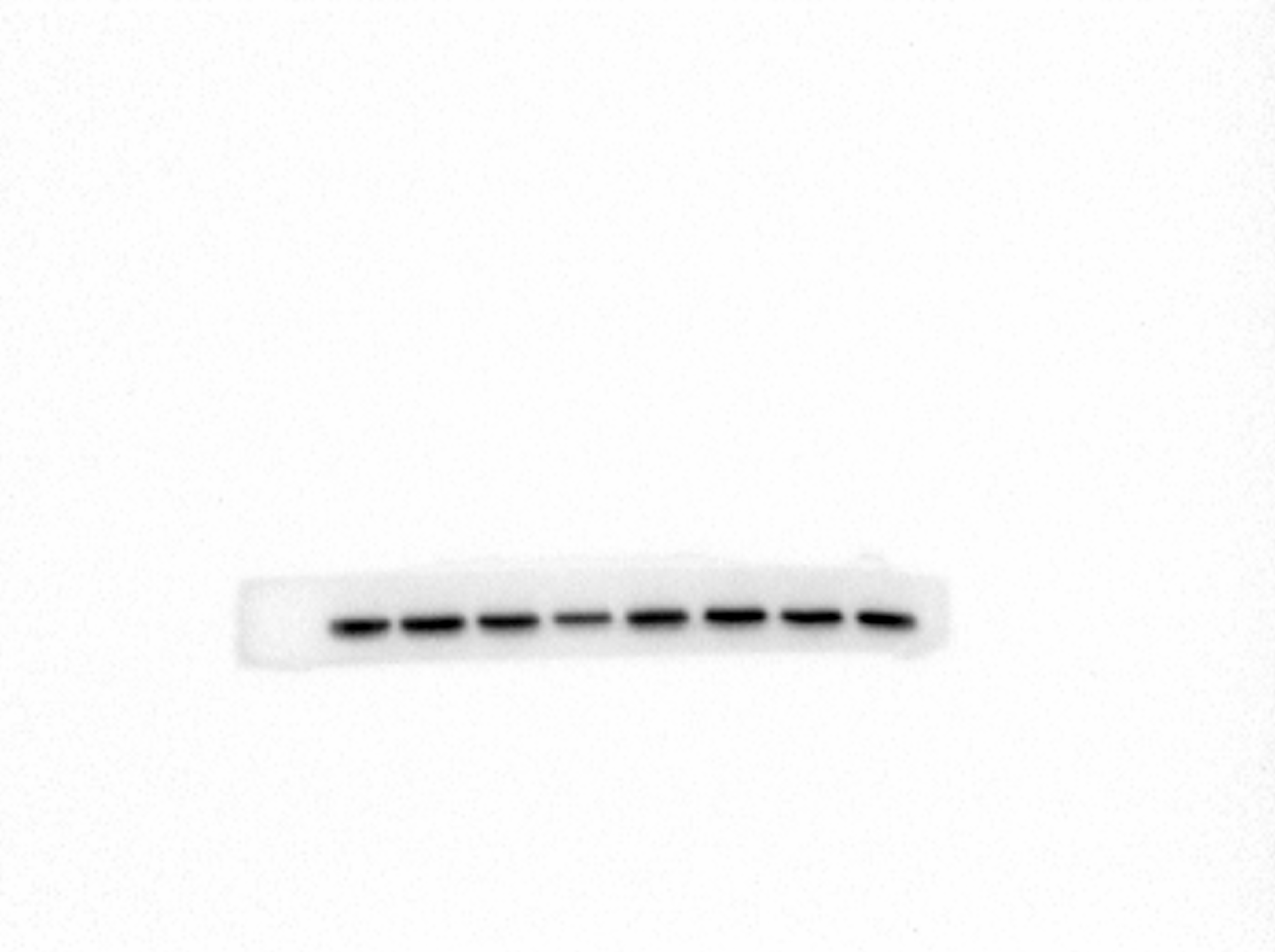

Supplement: Figure 5—source data 2. [file elife-99225-fig5-data2.zip › Figure 5-source data 2/F/MDA-MB-231/tubulin-231-dose.tif]

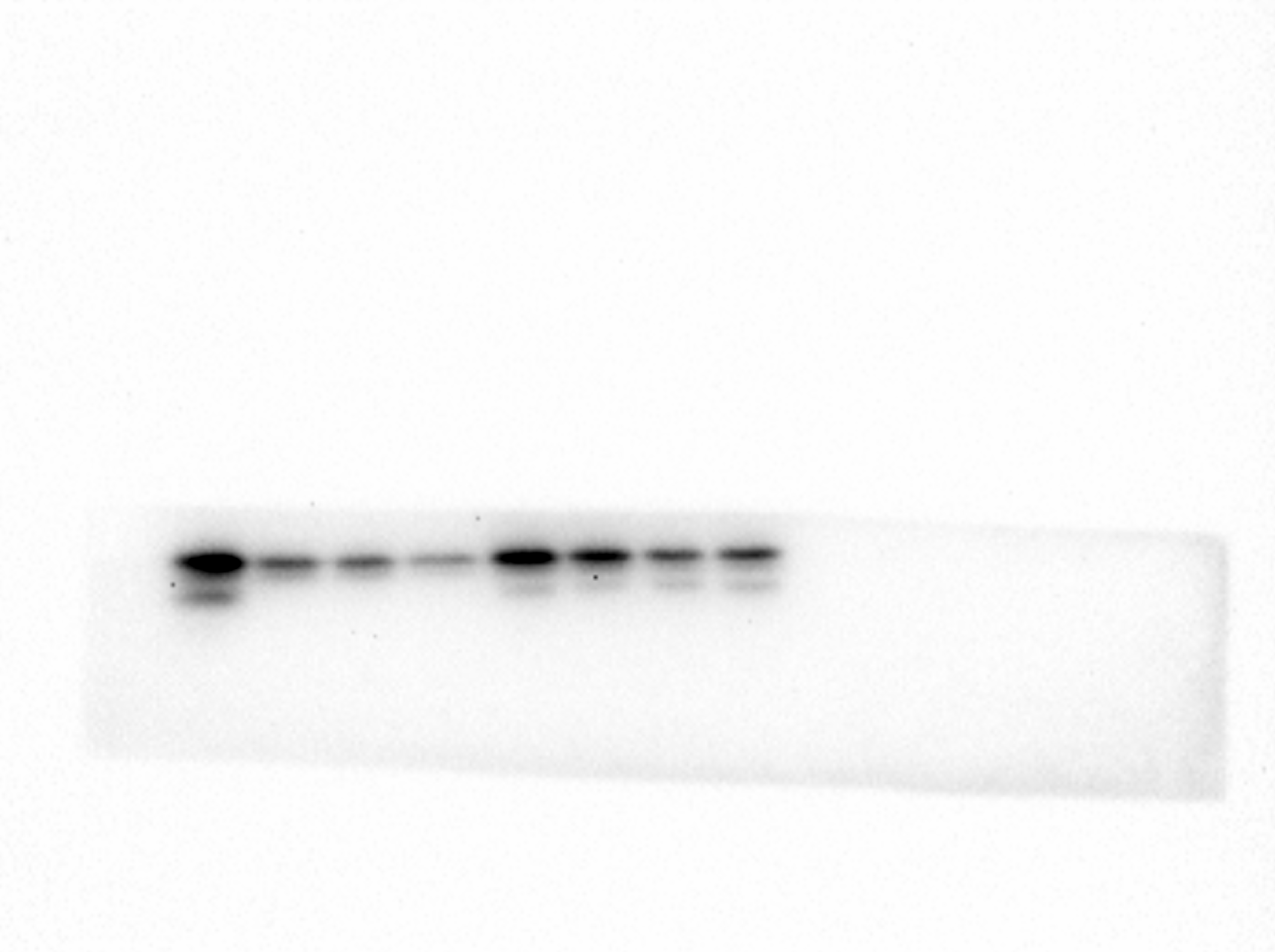

Supplement: Figure 5—source data 2. [file elife-99225-fig5-data2.zip › Figure 5-source data 2/F/MDA-MB-231/ercc1-231-dose.tif]

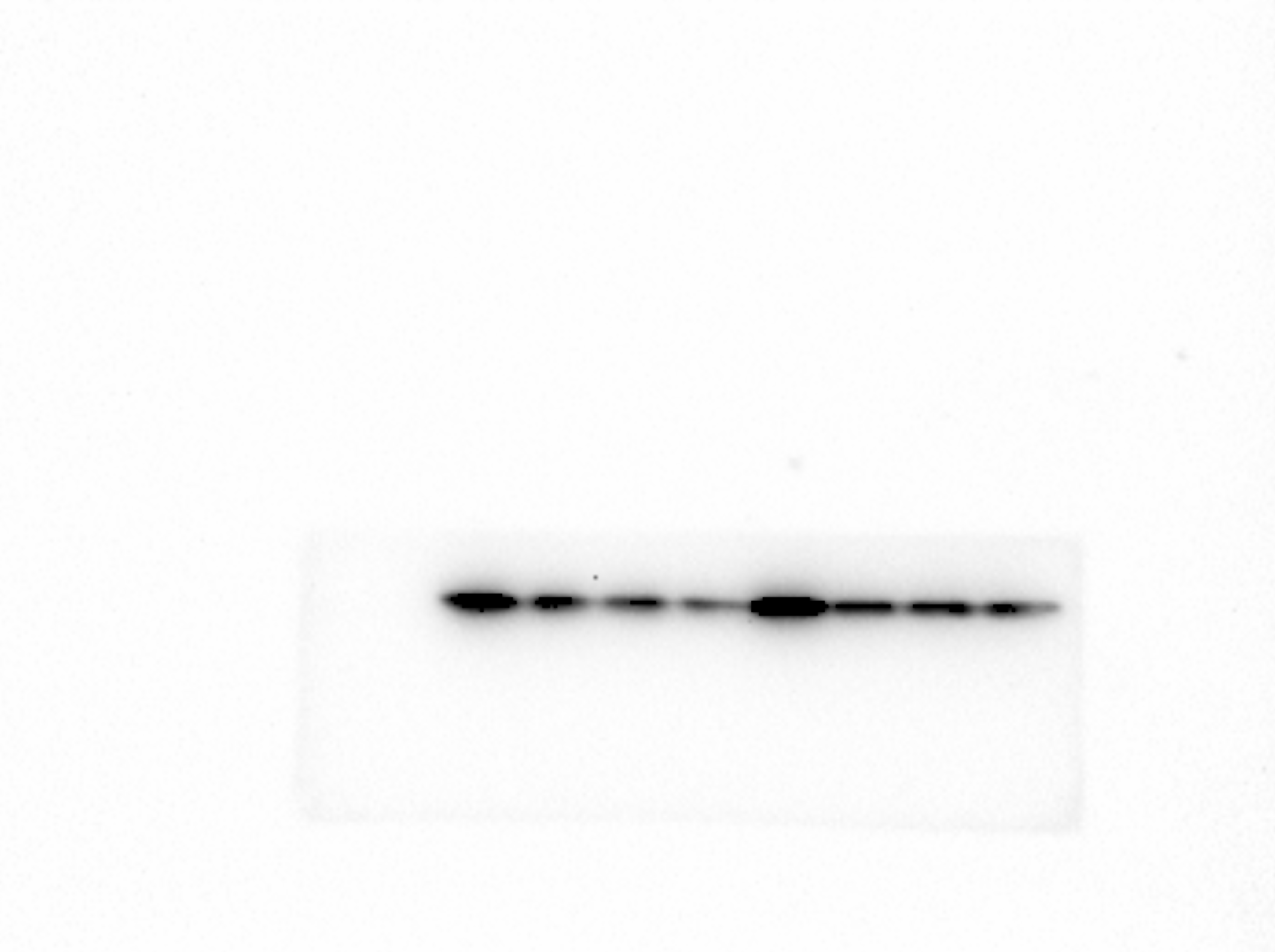

Supplement: Figure 5—source data 2. [file elife-99225-fig5-data2.zip › Figure 5-source data 2/F/MDA-MB-468/ercc1-468-dose.tif]

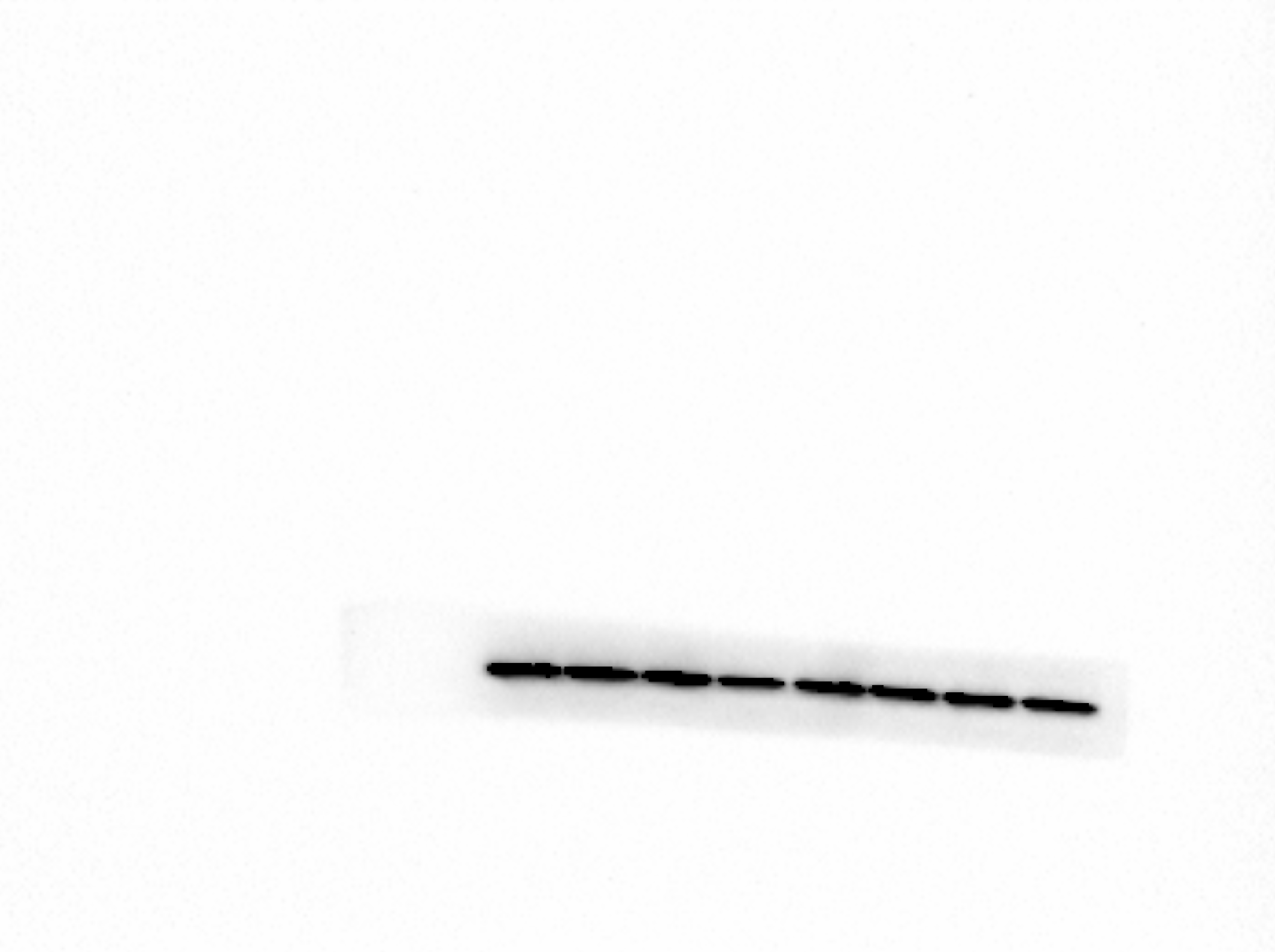

Supplement: Figure 5—source data 2. [file elife-99225-fig5-data2.zip › Figure 5-source data 2/F/MDA-MB-468/tubulin-468-dose.tif]

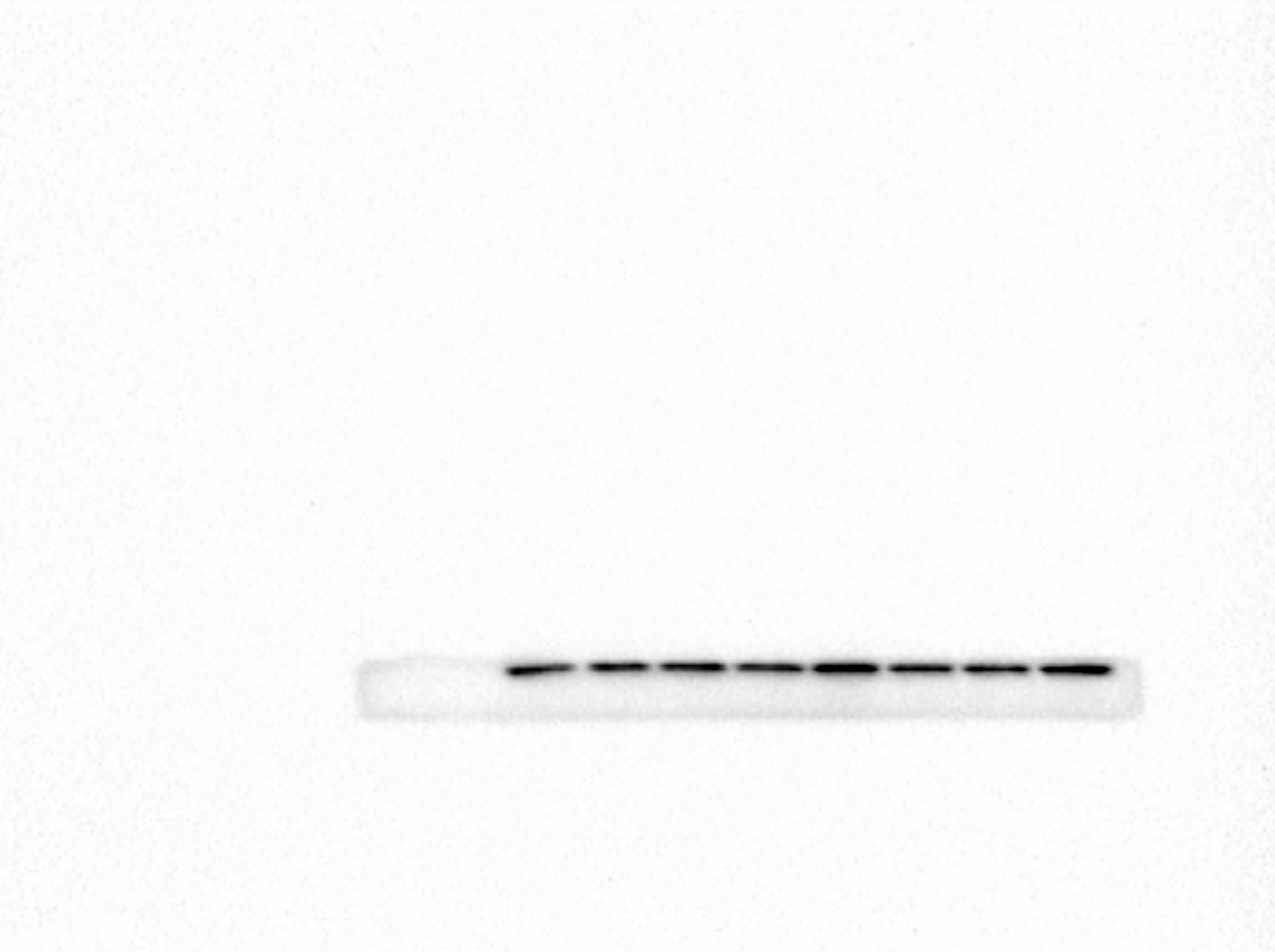

Supplement: Figure 5—source data 2. [file elife-99225-fig5-data2.zip › Figure 5-source data 2/F/OVCAR3/tubulin-ov3-dose.tif]

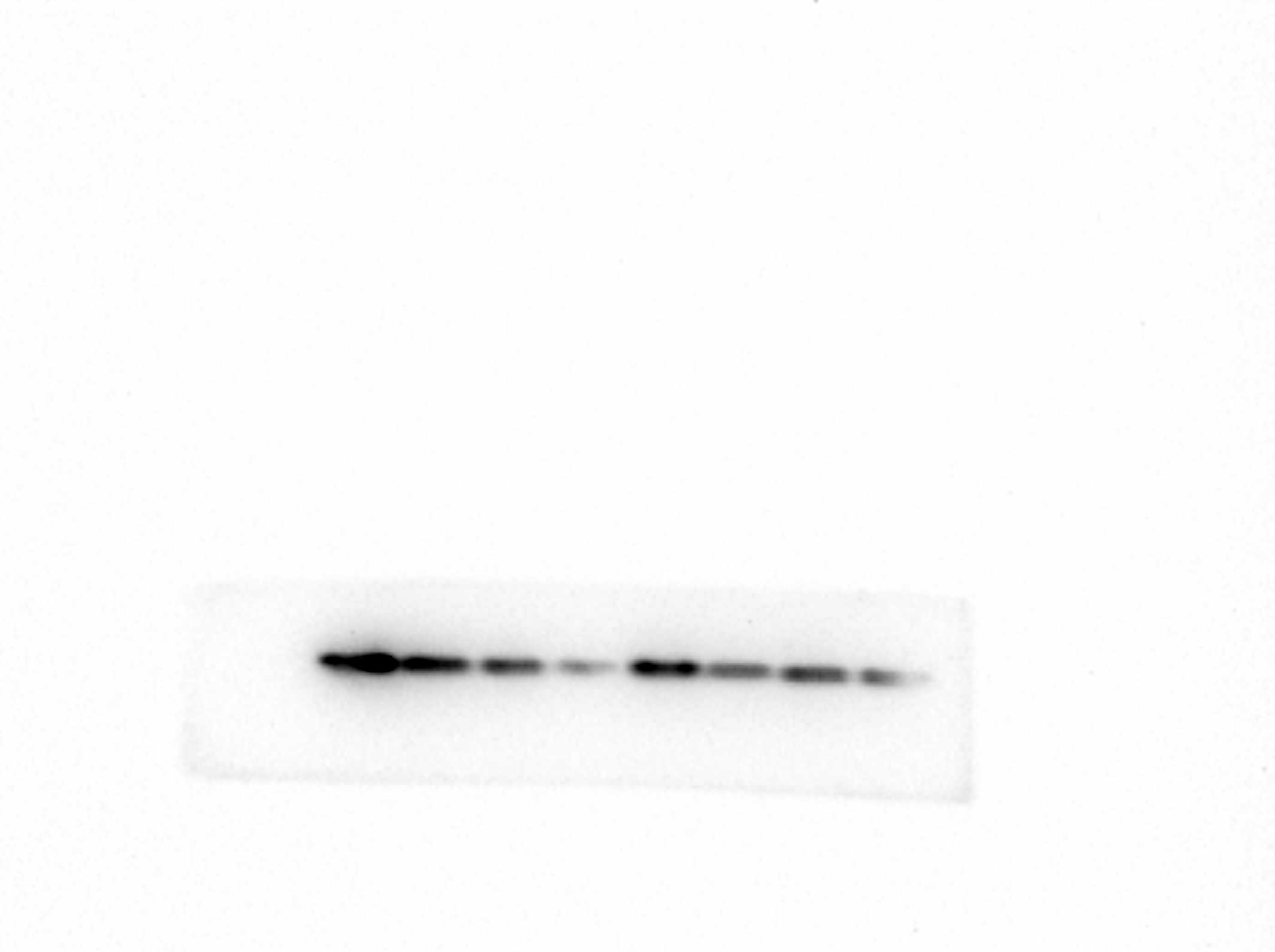

Supplement: Figure 5—source data 2. [file elife-99225-fig5-data2.zip › Figure 5-source data 2/F/OVCAR3/ercc1-ov3-dose.tif]

Figure 5-figure supplement 1

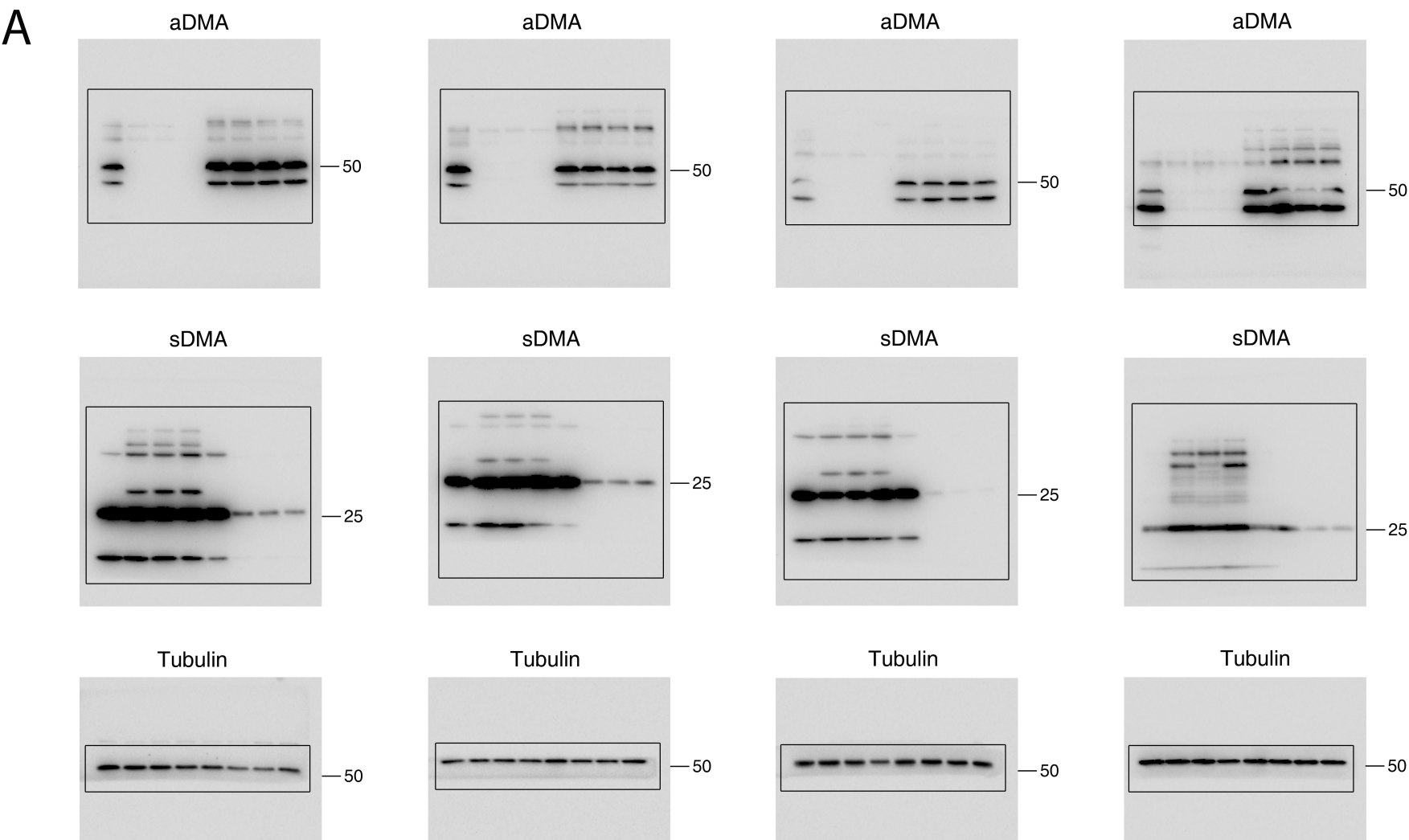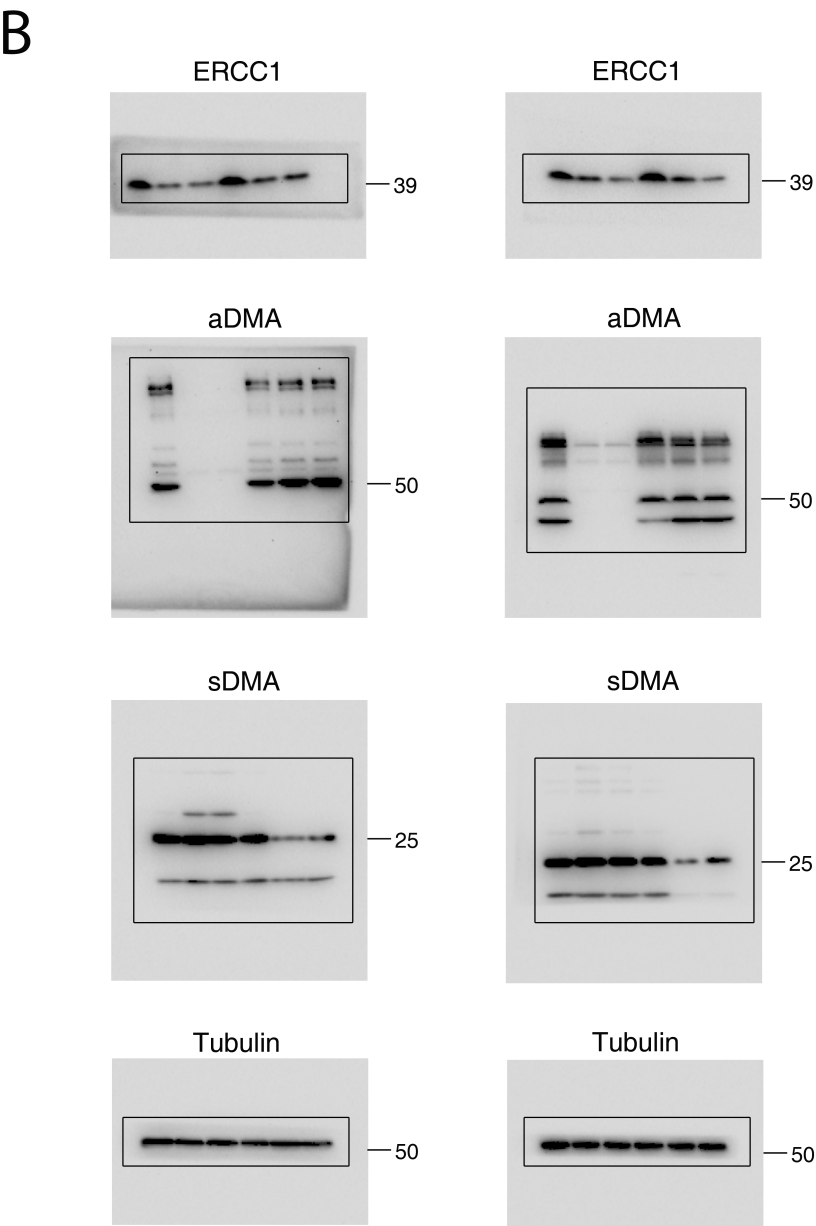

Supplement: Figure 5—figure supplement 1—source data 1. [file elife-99225-fig5-figsupp1-data1.pdf]

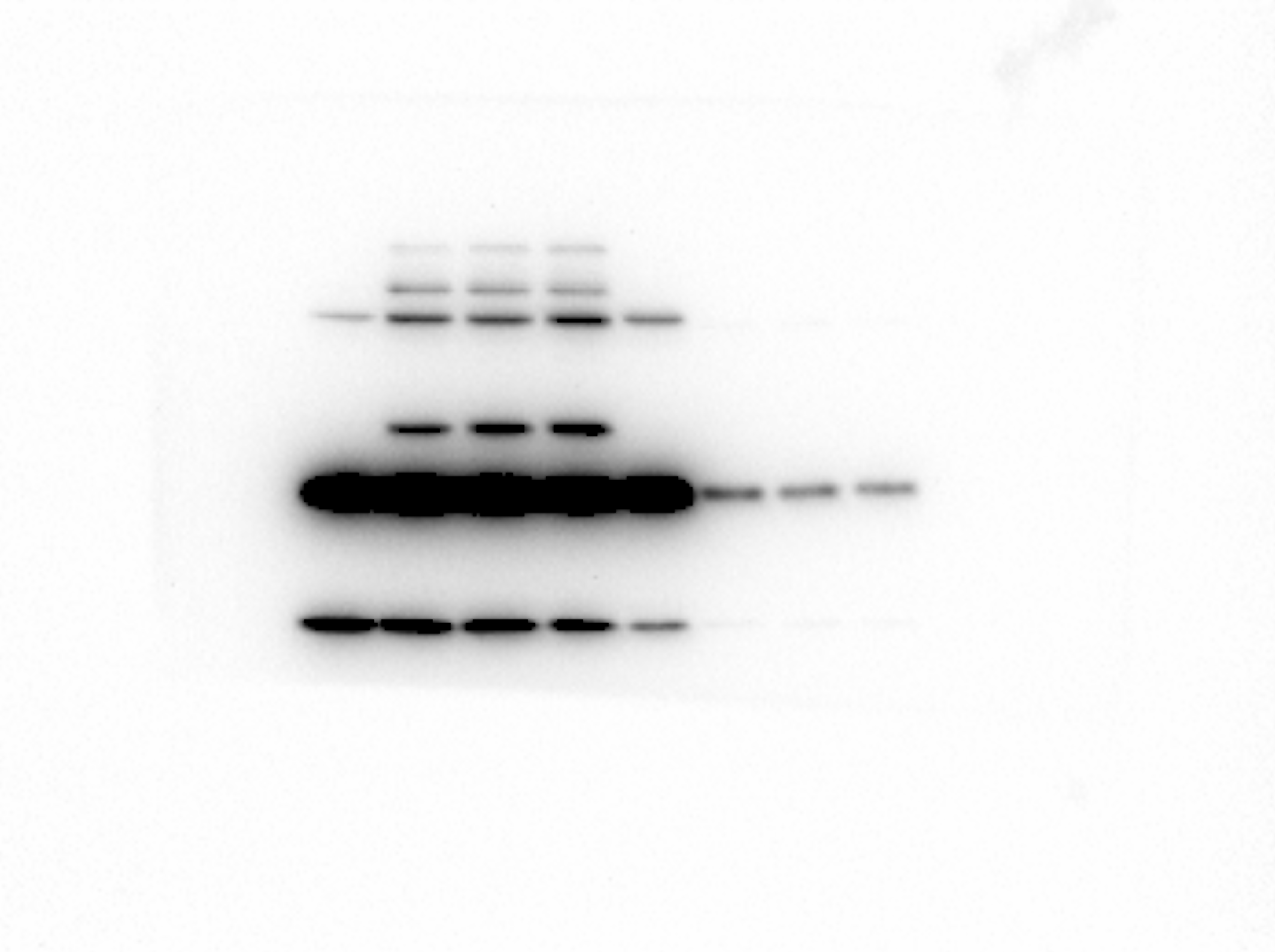

Supplement: Figure 5—figure supplement 1—source data 2. [file elife-99225-fig5-figsupp1-data2.zip › Figure 5-figure supplement 1-source data 2/A/OVCAR8/sdma-ov8-dose.tif]

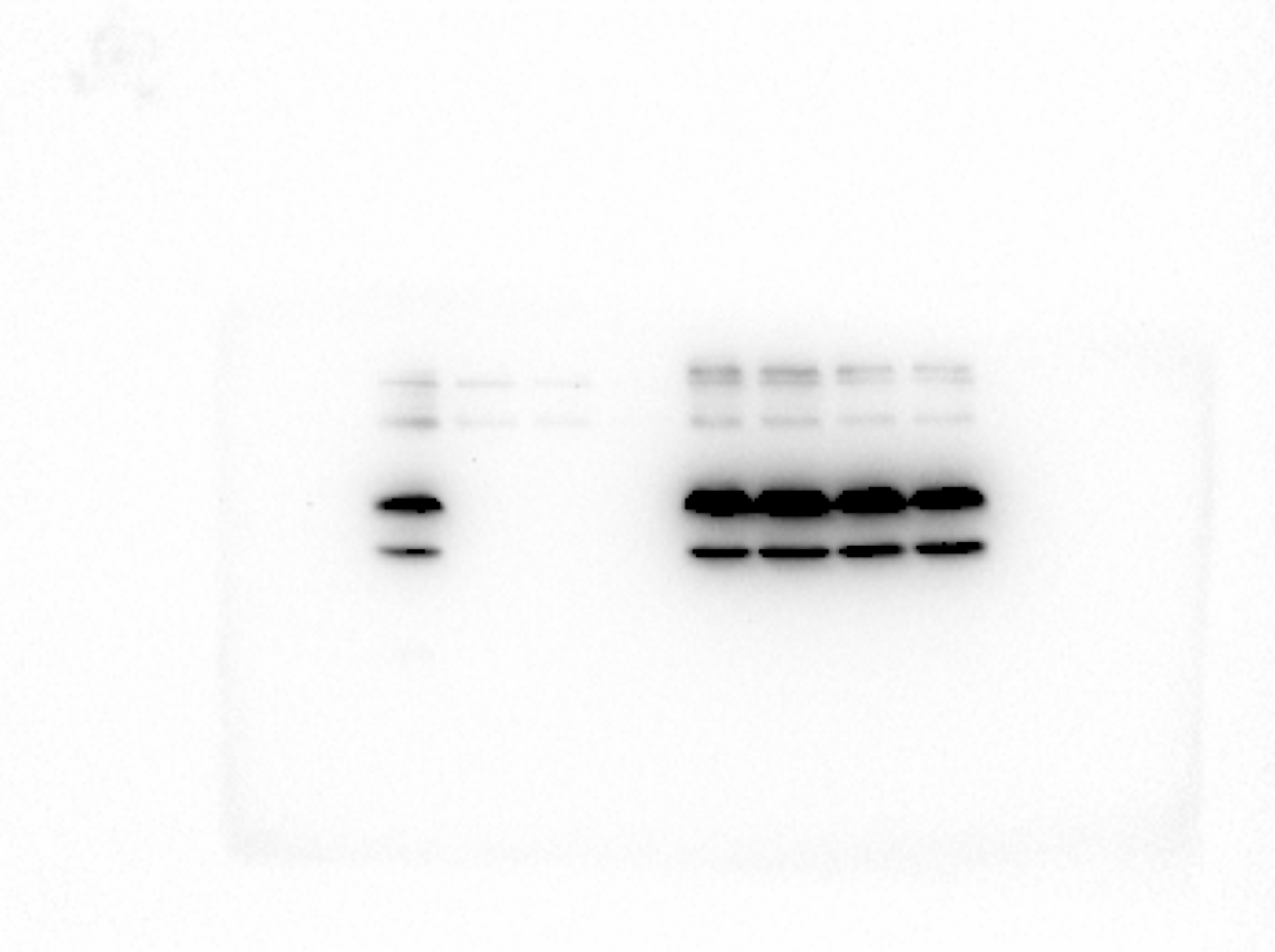

Supplement: Figure 5—figure supplement 1—source data 2. [file elife-99225-fig5-figsupp1-data2.zip › Figure 5-figure supplement 1-source data 2/A/OVCAR8/adma-ov8-dose.tif]

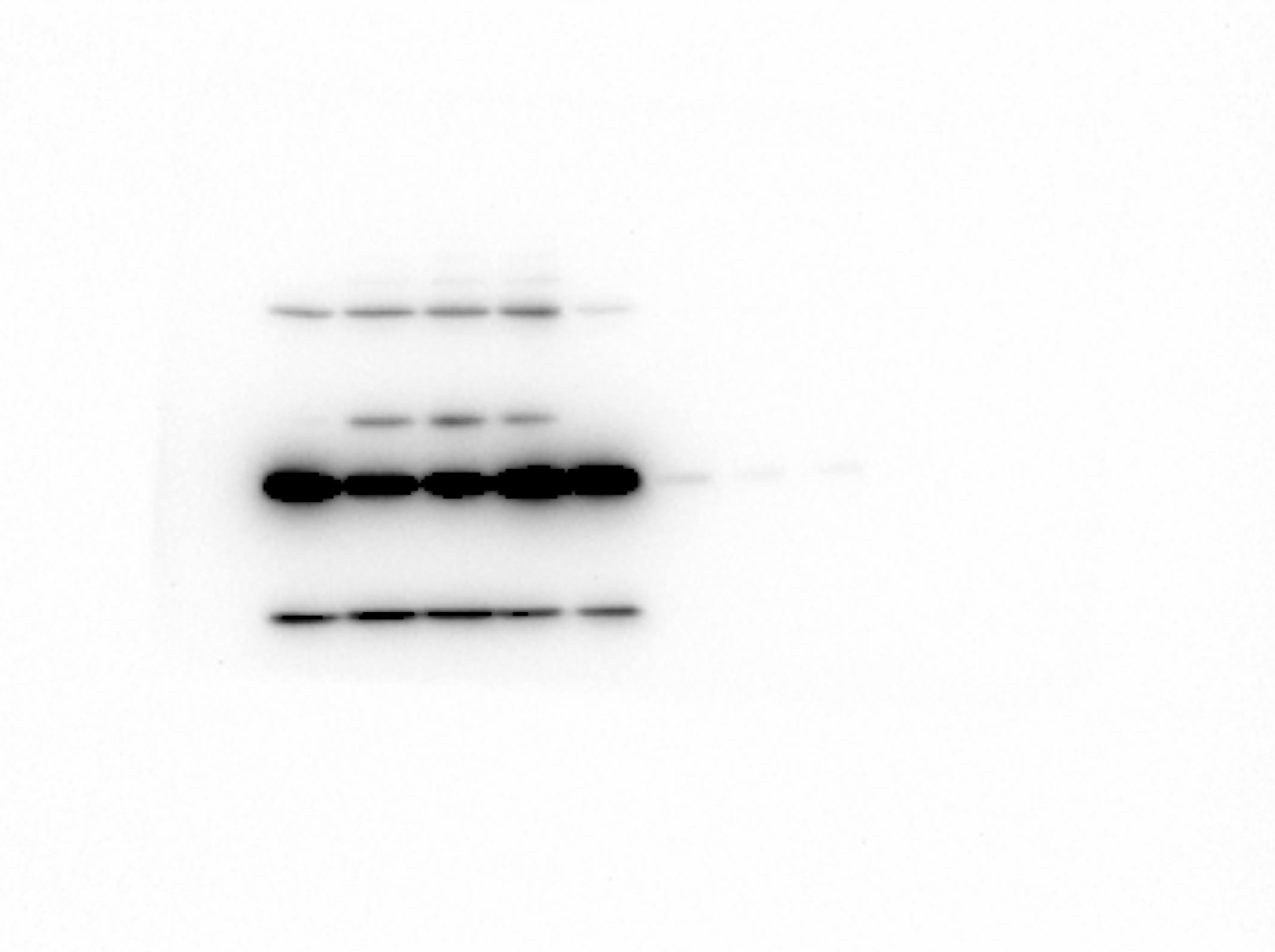

Supplement: Figure 5—figure supplement 1—source data 2. [file elife-99225-fig5-figsupp1-data2.zip › Figure 5-figure supplement 1-source data 2/A/MDA-MB-231/sdma-231-dose.tif]

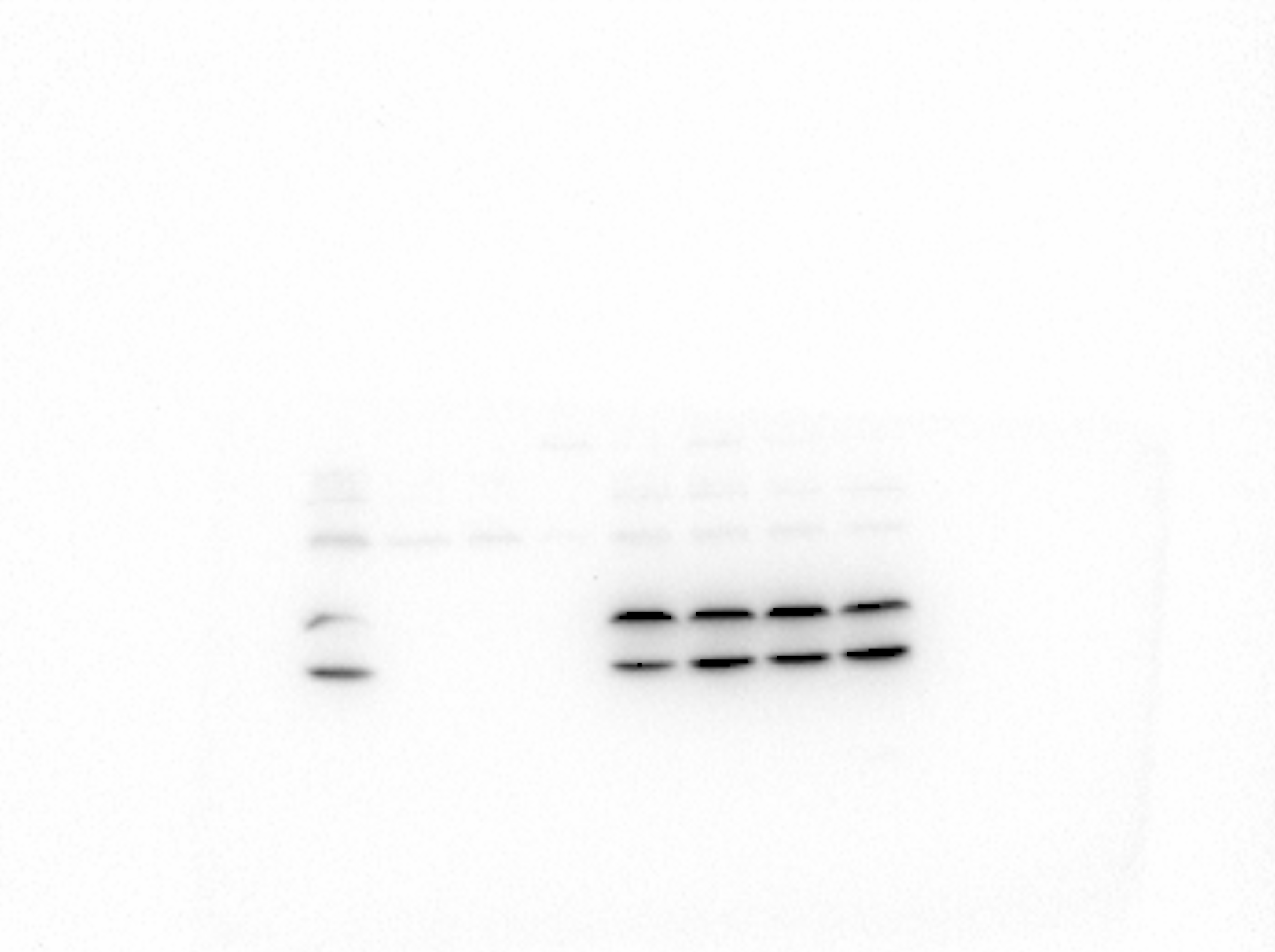

Supplement: Figure 5—figure supplement 1—source data 2. [file elife-99225-fig5-figsupp1-data2.zip › Figure 5-figure supplement 1-source data 2/A/MDA-MB-231/231-adma-dose.tif]

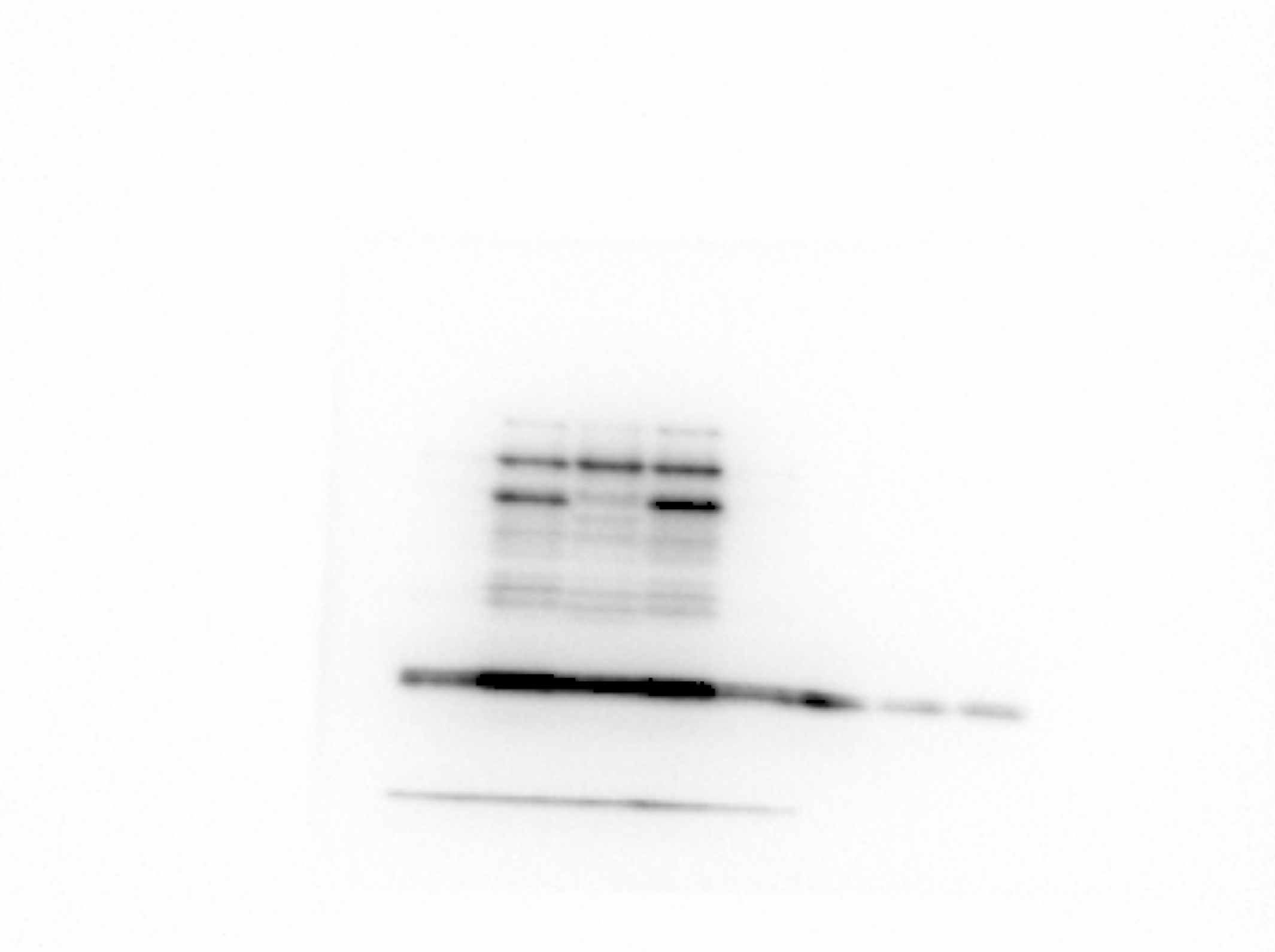

Supplement: Figure 5—figure supplement 1—source data 2. [file elife-99225-fig5-figsupp1-data2.zip › Figure 5-figure supplement 1-source data 2/A/MDA-MB-468/sdma-468-dose.tif]

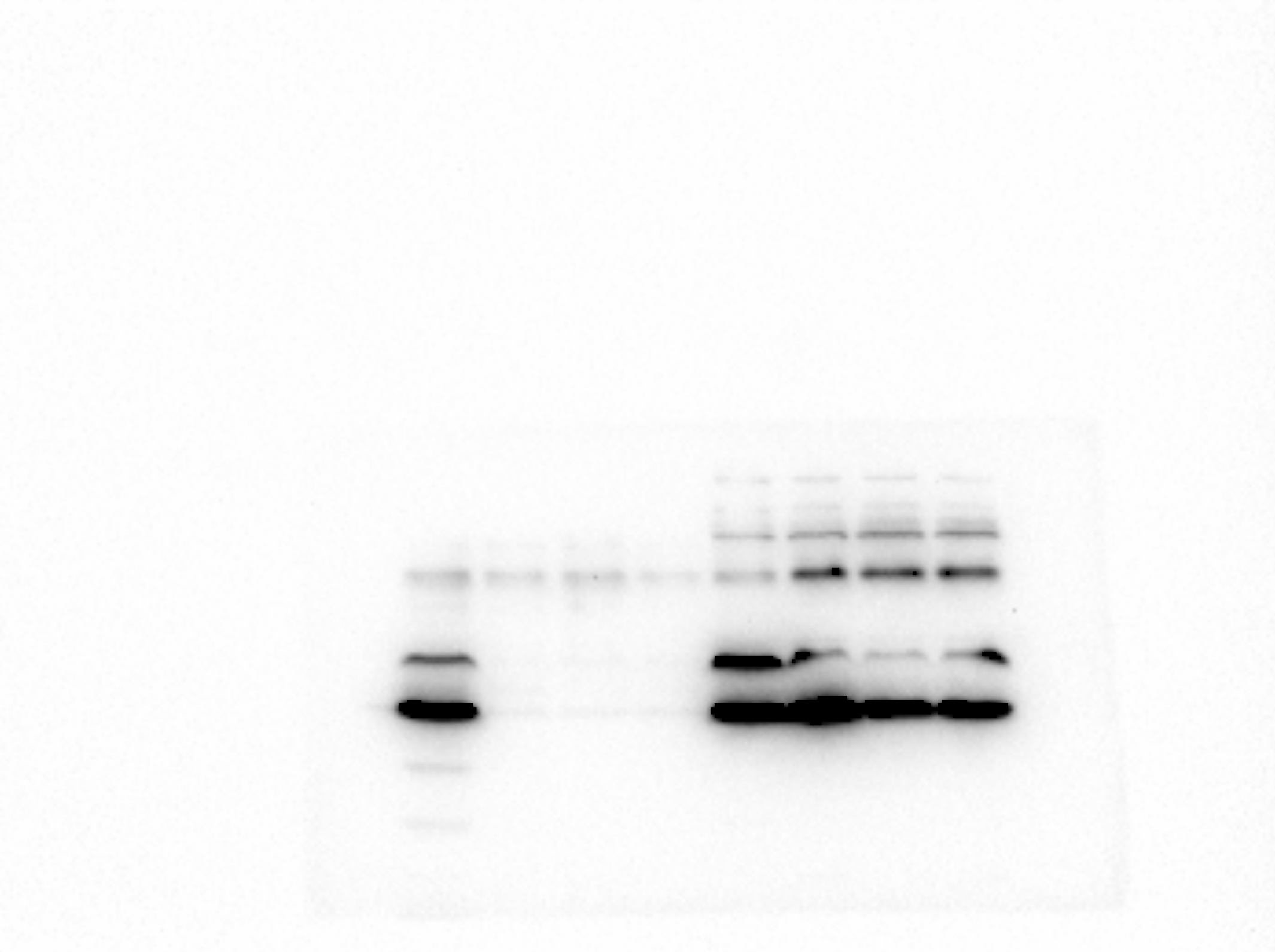

Supplement: Figure 5—figure supplement 1—source data 2. [file elife-99225-fig5-figsupp1-data2.zip › Figure 5-figure supplement 1-source data 2/A/MDA-MB-468/adma-468-dose.tif]

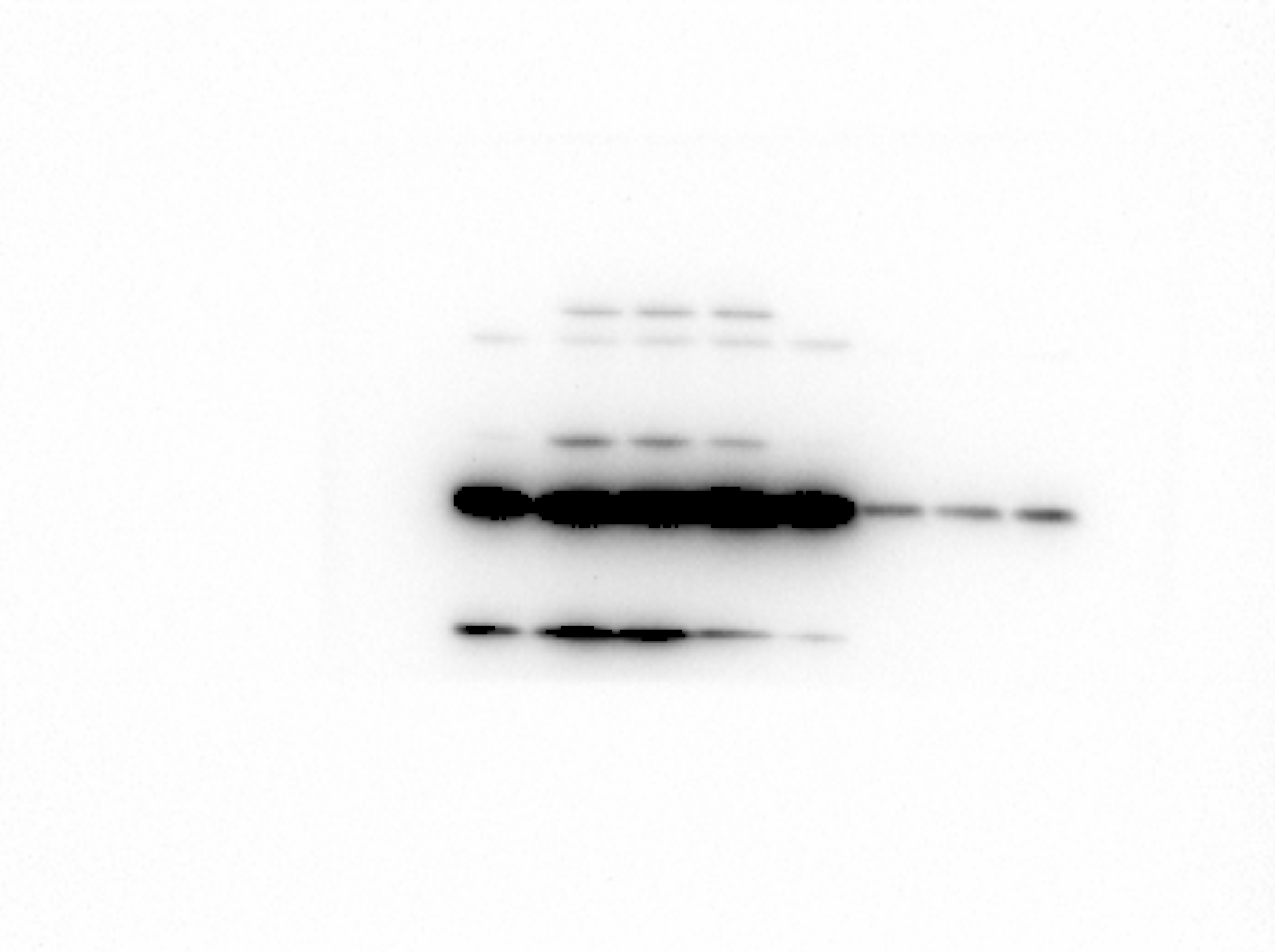

Supplement: Figure 5—figure supplement 1—source data 2. [file elife-99225-fig5-figsupp1-data2.zip › Figure 5-figure supplement 1-source data 2/A/OVCAR3/sdma-ov3-dose.tif]

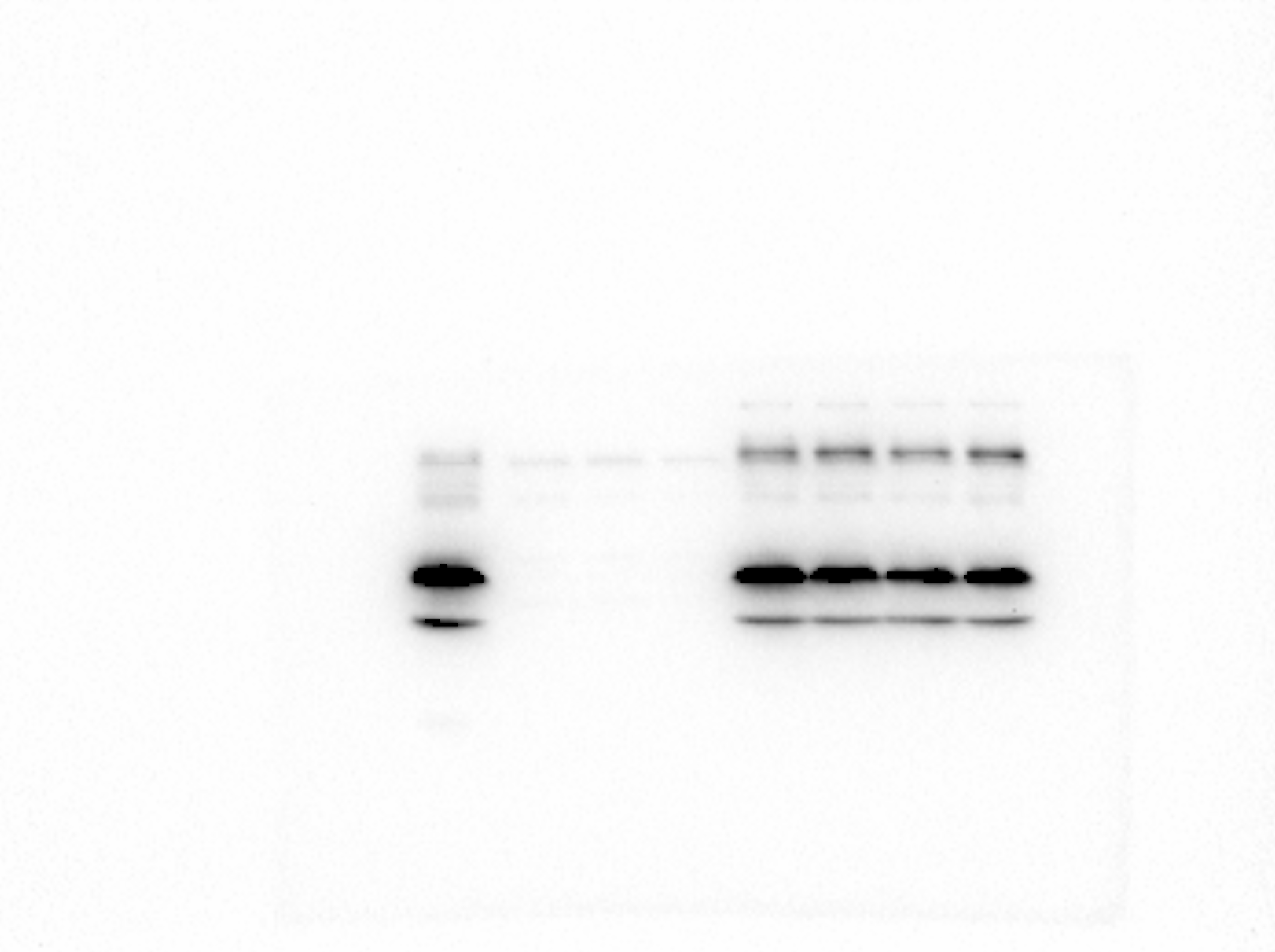

Supplement: Figure 5—figure supplement 1—source data 2. [file elife-99225-fig5-figsupp1-data2.zip › Figure 5-figure supplement 1-source data 2/A/OVCAR3/adma-ov3-dose.tif]

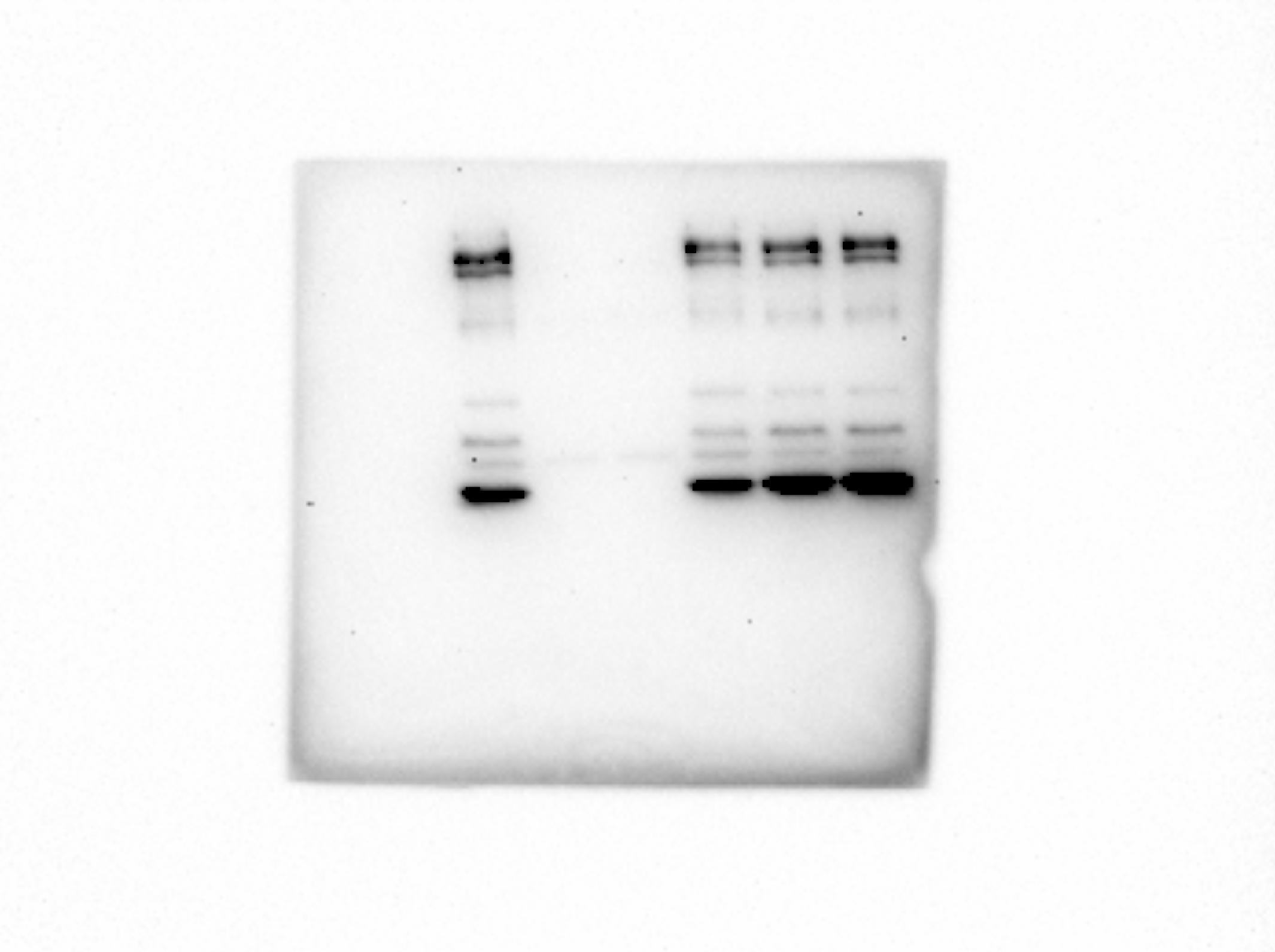

Supplement: Figure 5—figure supplement 1—source data 2. [file elife-99225-fig5-figsupp1-data2.zip › Figure 5-figure supplement 1-source data 2/B/OVCAR8/adma-ov8-prmtsg.tif]

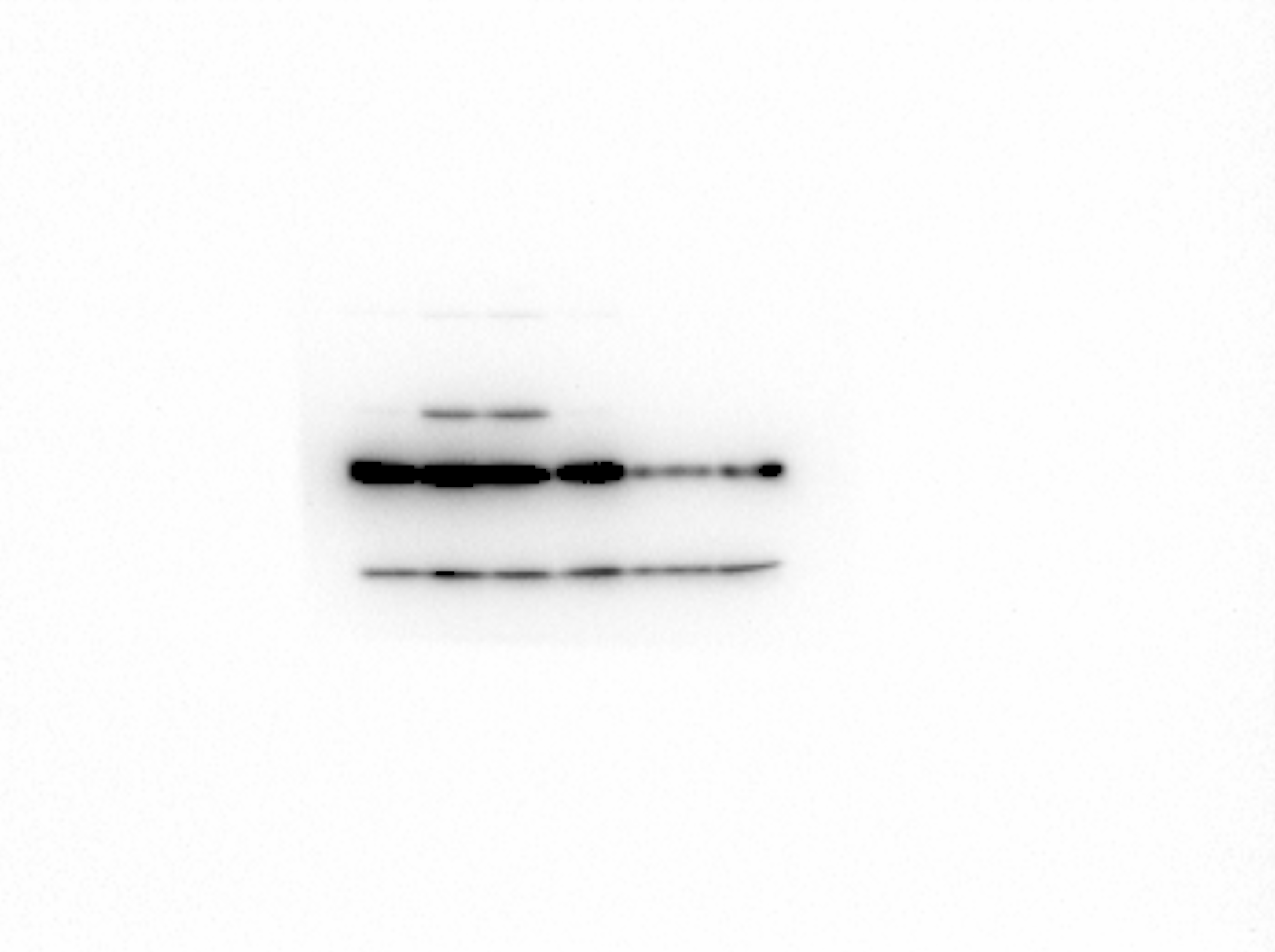

Supplement: Figure 5—figure supplement 1—source data 2. [file elife-99225-fig5-figsupp1-data2.zip › Figure 5-figure supplement 1-source data 2/B/OVCAR8/sdma-ov8-prmtsg.tif]

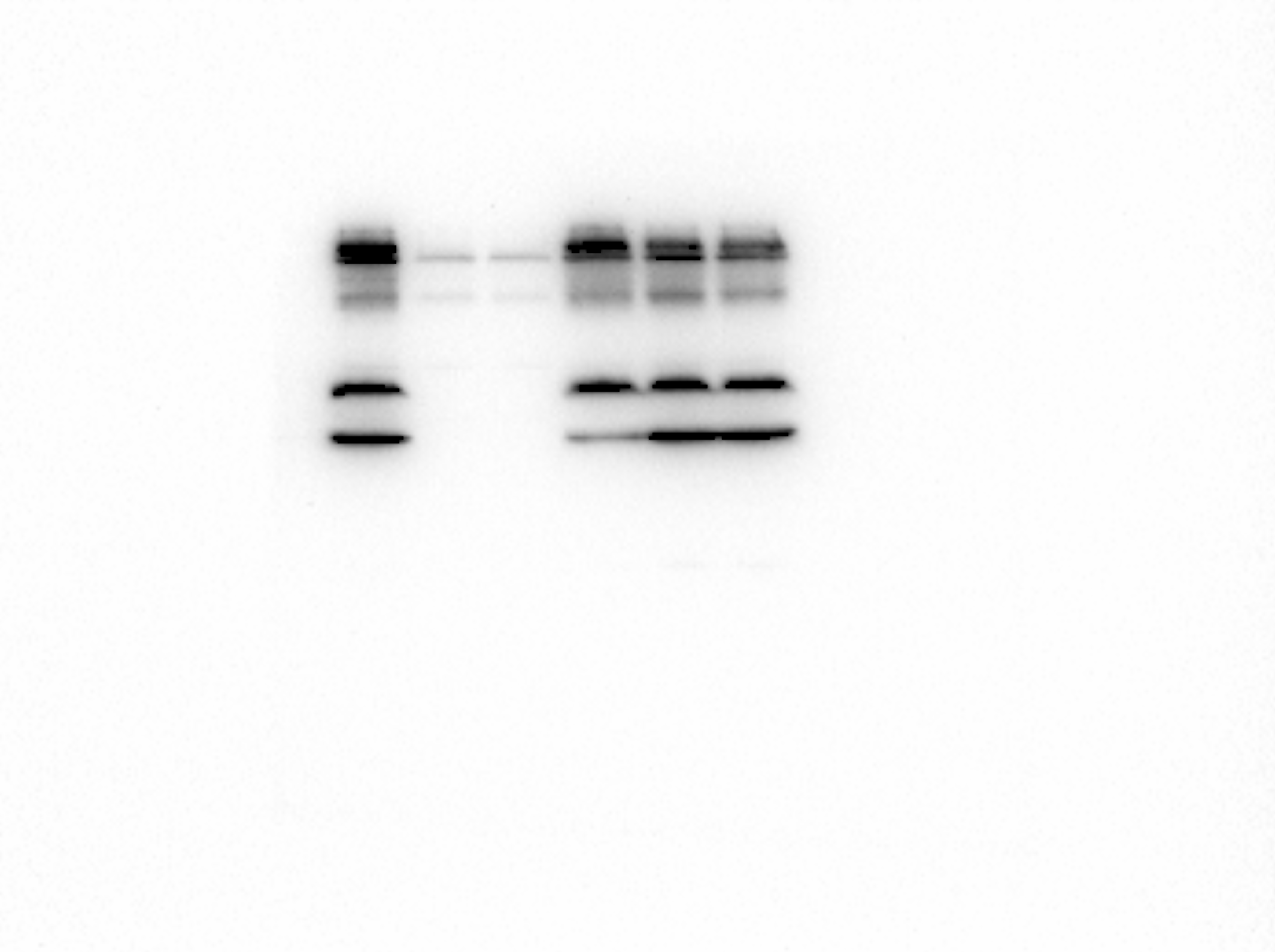

Supplement: Figure 5—figure supplement 1—source data 2. [file elife-99225-fig5-figsupp1-data2.zip › Figure 5-figure supplement 1-source data 2/B/MDA-MB-231/adma-231-prmtsg.tif]

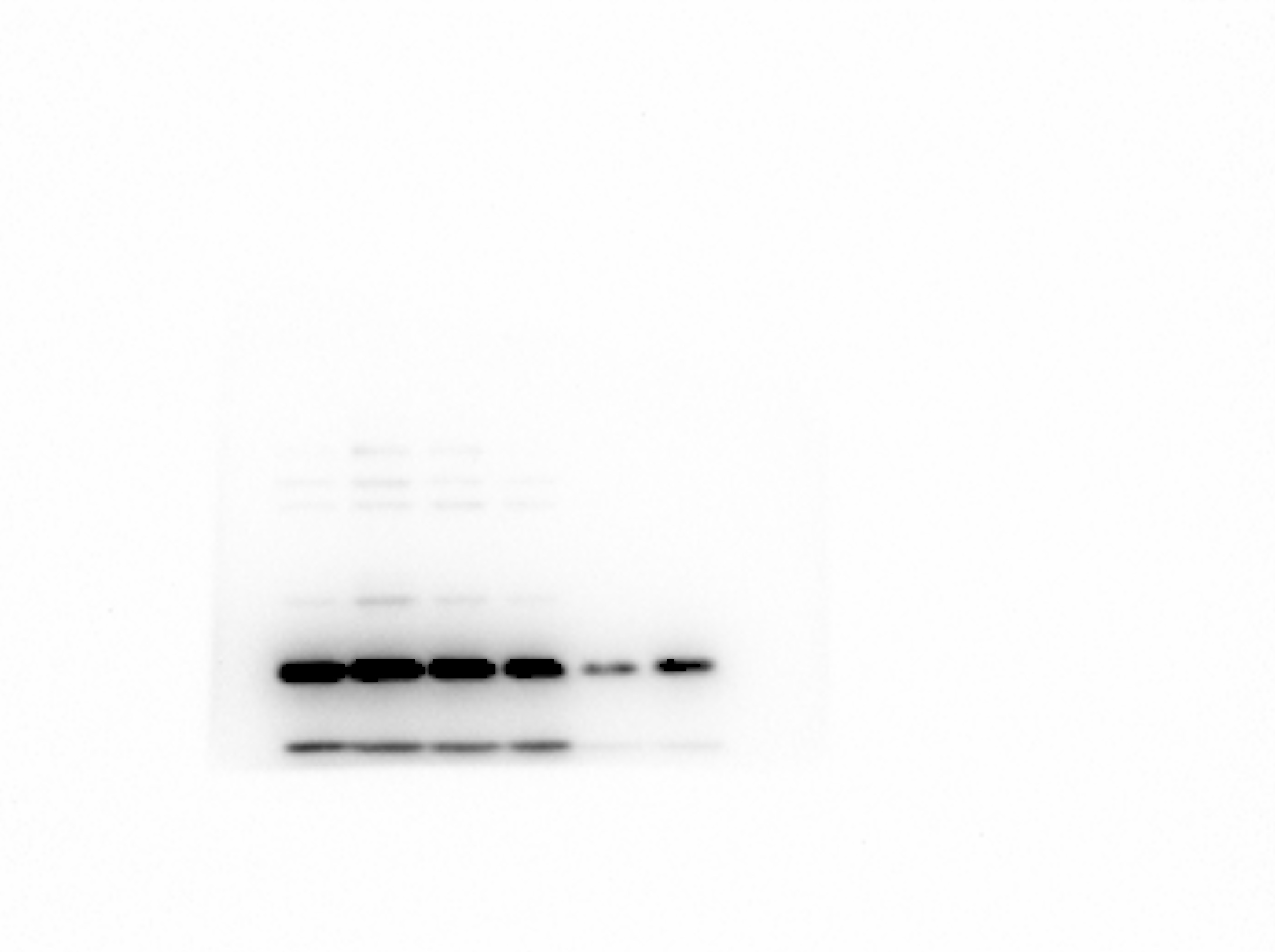

Supplement: Figure 5—figure supplement 1—source data 2. [file elife-99225-fig5-figsupp1-data2.zip › Figure 5-figure supplement 1-source data 2/B/MDA-MB-231/sdma-231-prmtsg.tif]

Figure 5-figure supplement 2

E

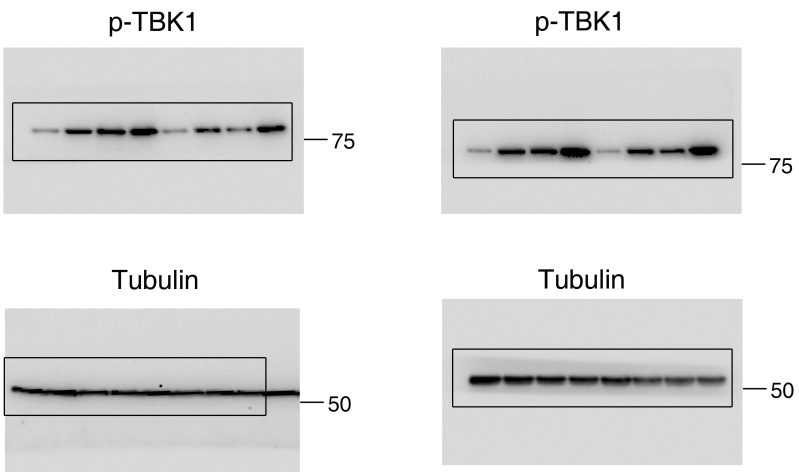

F

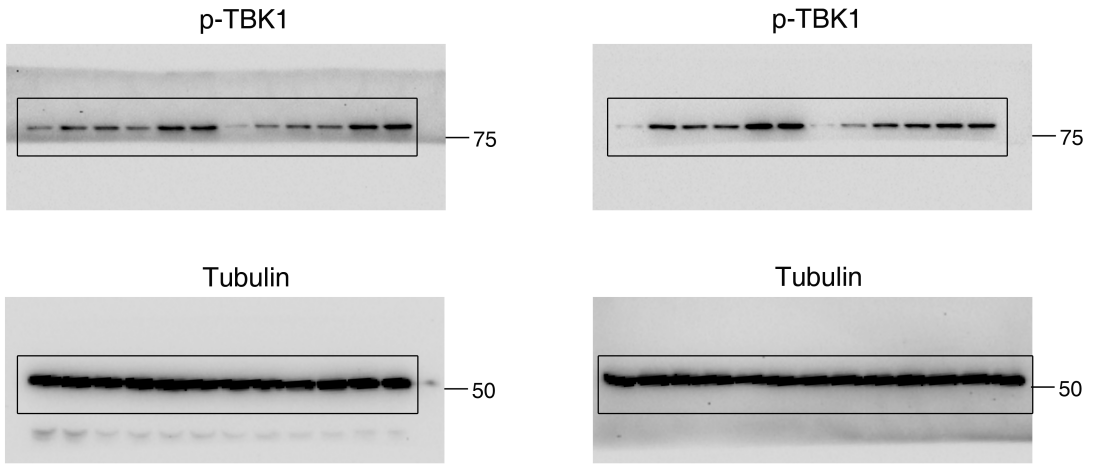

Supplement: Figure 5—figure supplement 2—source data 1. [file elife-99225-fig5-figsupp2-data1.pdf]

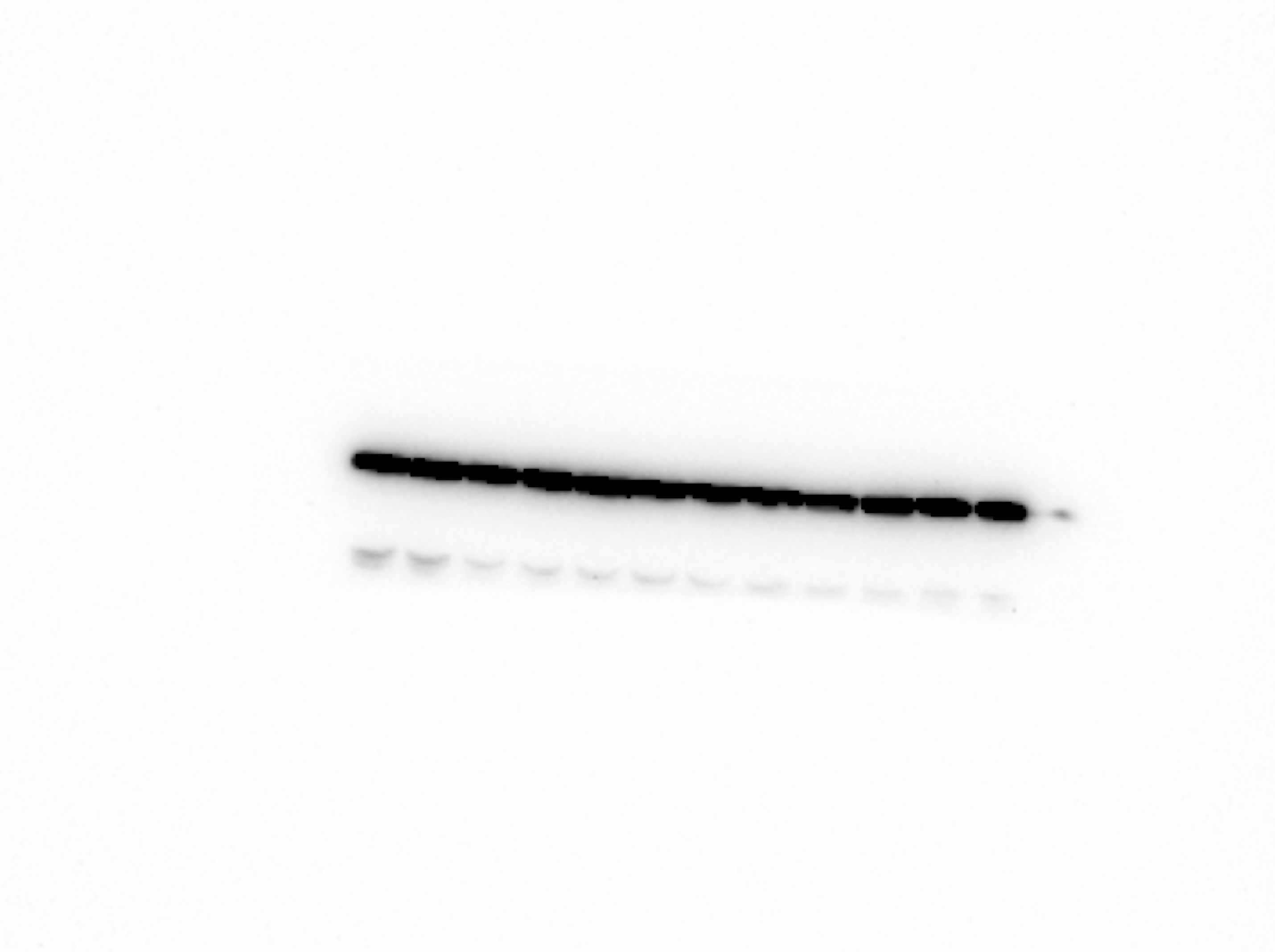

Supplement: Figure 5—figure supplement 2—source data 2. [file elife-99225-fig5-figsupp2-data2.zip › Figure 5-figure supplement 2-source data 2/F/OVCAR8/tubulin-ov8-sgola.tif]

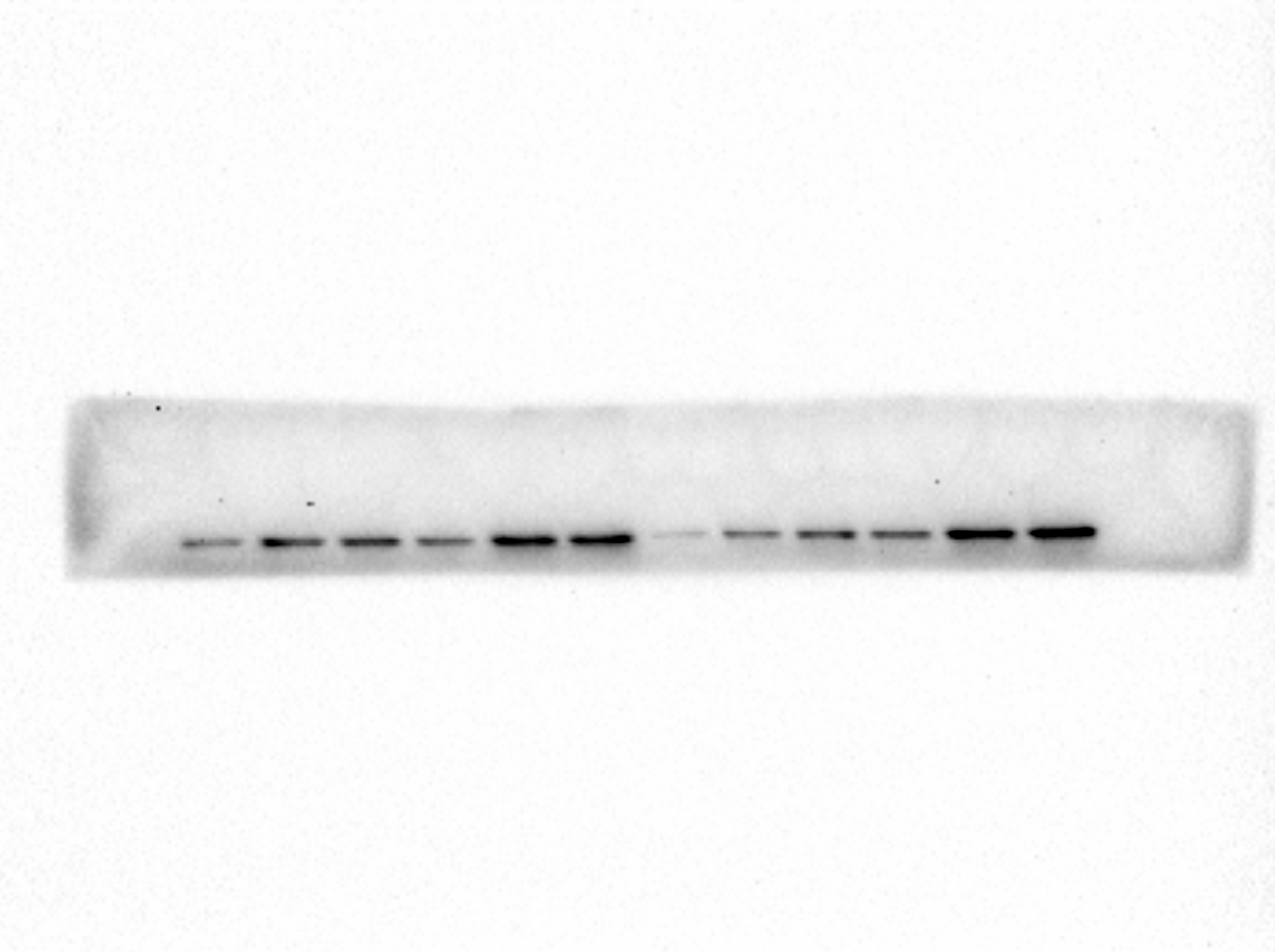

Supplement: Figure 5—figure supplement 2—source data 2. [file elife-99225-fig5-figsupp2-data2.zip › Figure 5-figure supplement 2-source data 2/F/OVCAR8/ptbk1-ov8-sgola.tif]

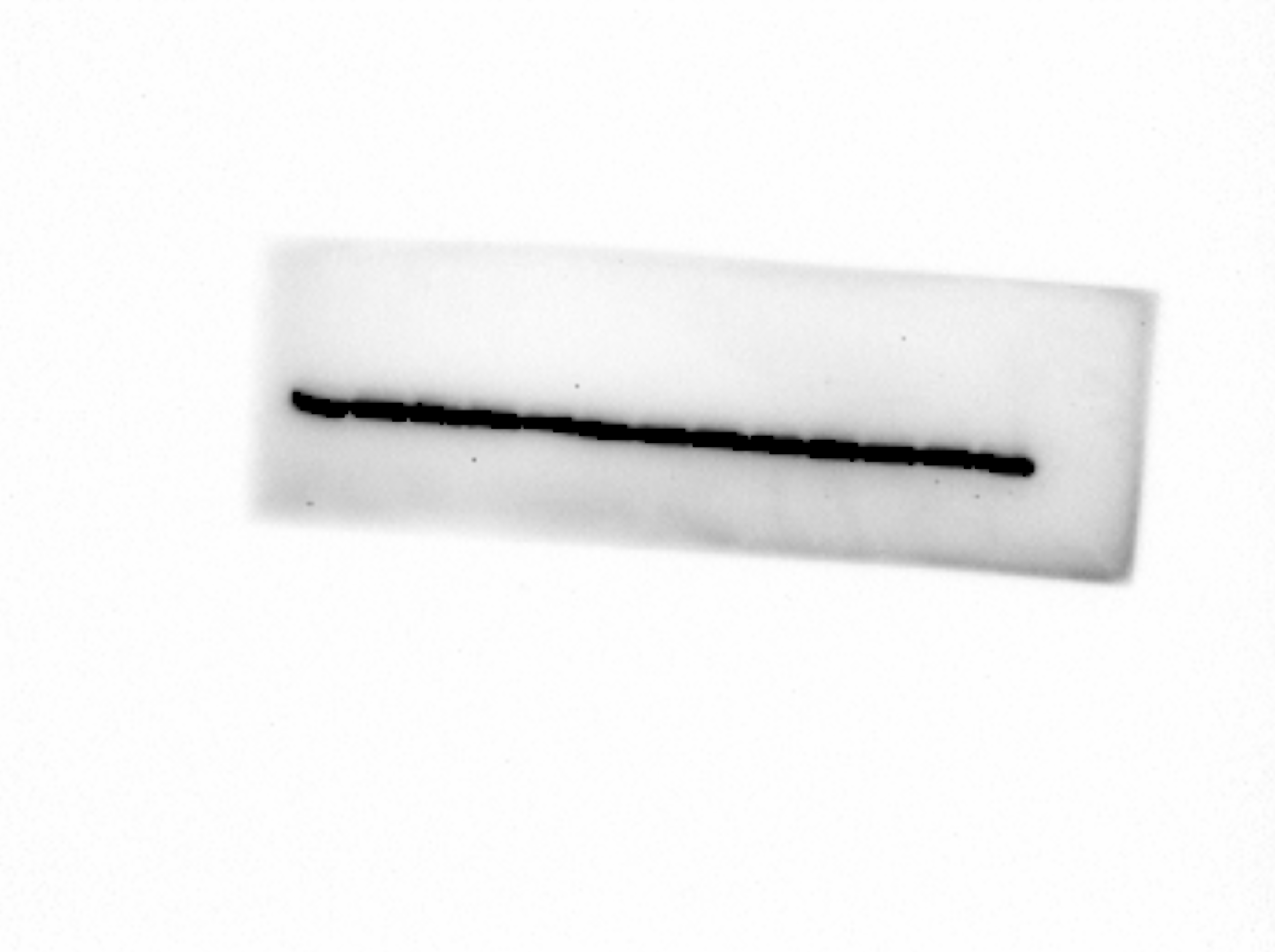

Supplement: Figure 5—figure supplement 2—source data 2. [file elife-99225-fig5-figsupp2-data2.zip › Figure 5-figure supplement 2-source data 2/F/MDA-MB-231/tubulin-231-sgola.tif]

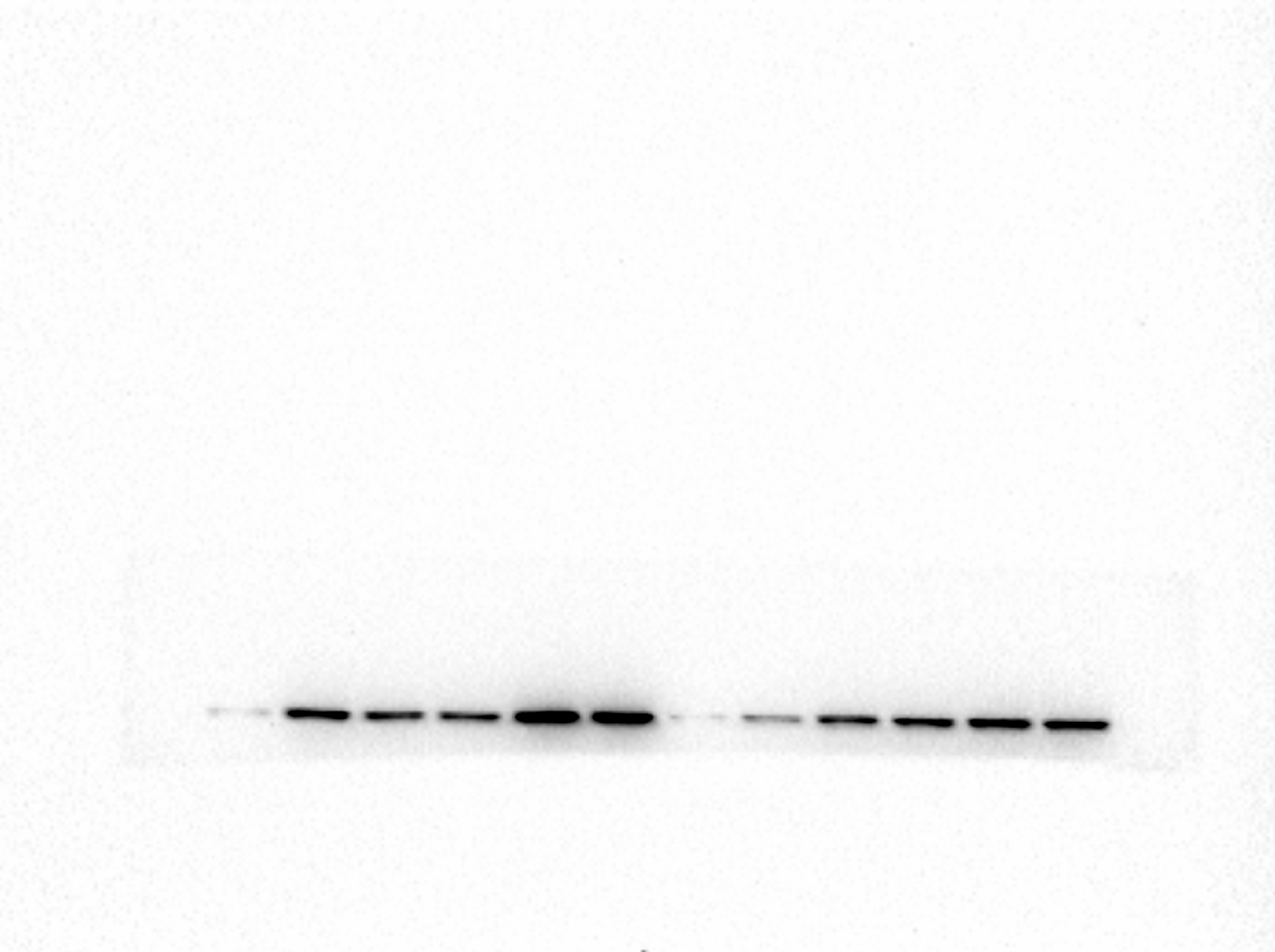

Supplement: Figure 5—figure supplement 2—source data 2. [file elife-99225-fig5-figsupp2-data2.zip › Figure 5-figure supplement 2-source data 2/F/MDA-MB-231/ptbk1-231-sgola.tif]

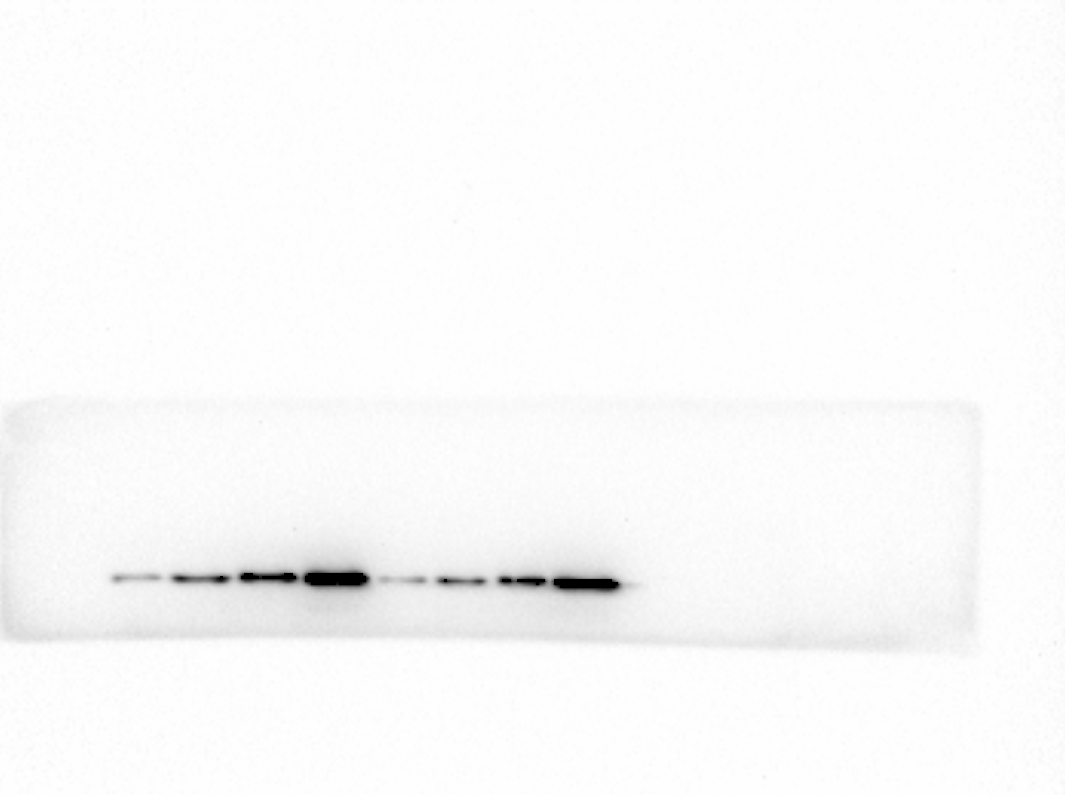

Supplement: Figure 5—figure supplement 2—source data 2. [file elife-99225-fig5-figsupp2-data2.zip › Figure 5-figure supplement 2-source data 2/E/OVCAR8/ptbk1-ov8-inmune.tif]

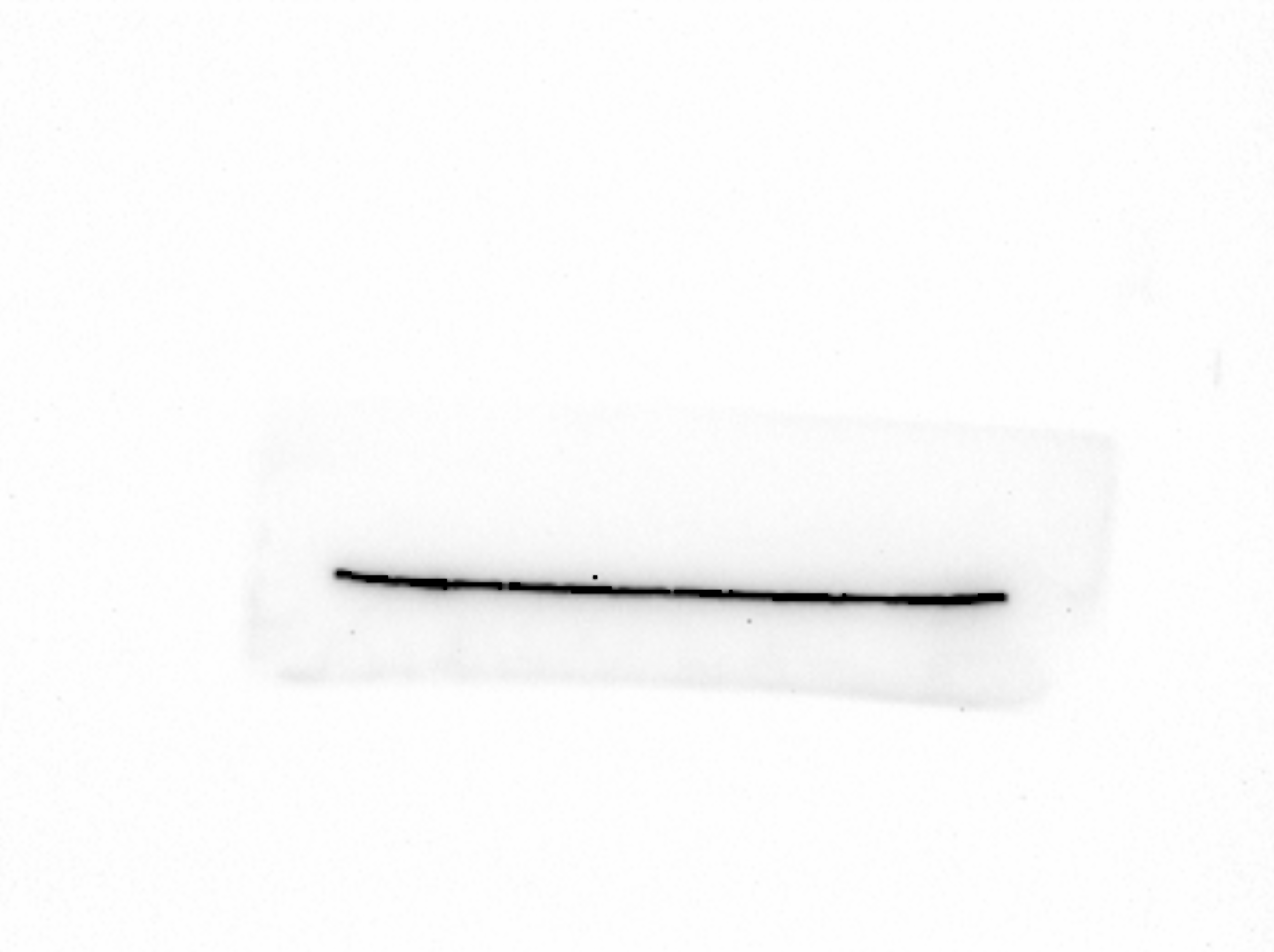

Supplement: Figure 5—figure supplement 2—source data 2. [file elife-99225-fig5-figsupp2-data2.zip › Figure 5-figure supplement 2-source data 2/E/OVCAR8/tubulin-ov8-inmune.tif]

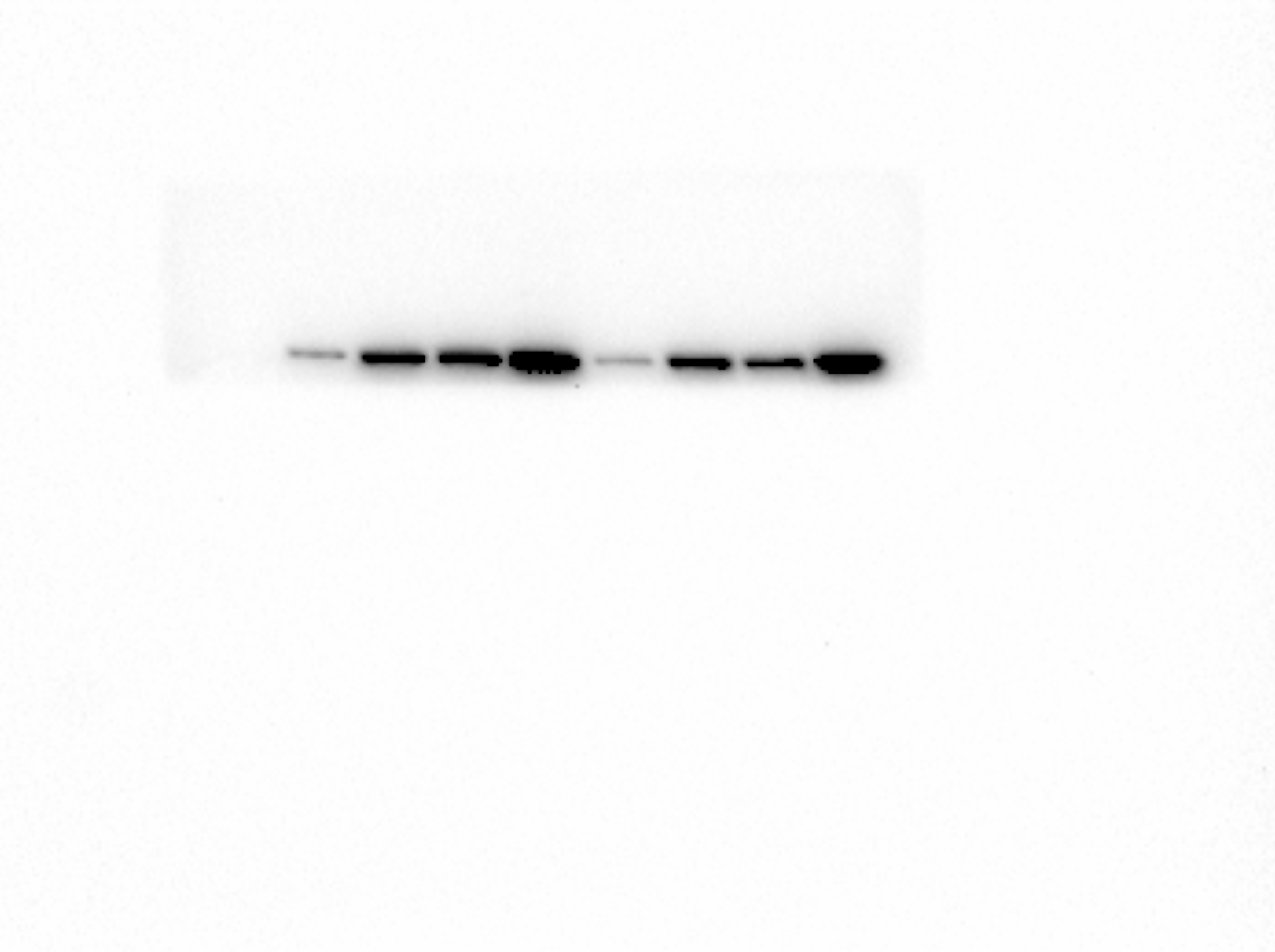

Supplement: Figure 5—figure supplement 2—source data 2. [file elife-99225-fig5-figsupp2-data2.zip › Figure 5-figure supplement 2-source data 2/E/MDA-MB-231/ptbk1-231-inmune.tif]

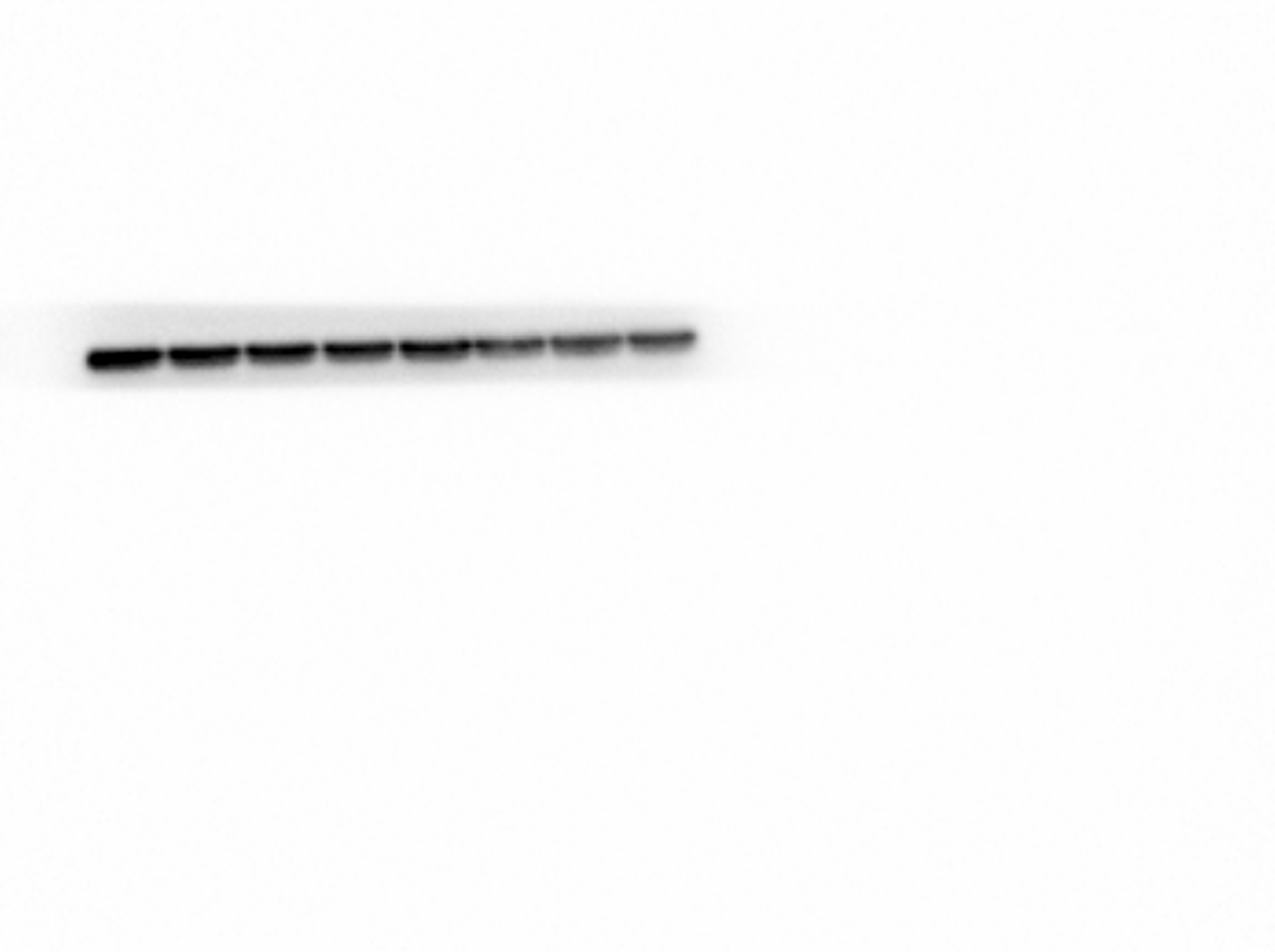

Supplement: Figure 5—figure supplement 2—source data 2. [file elife-99225-fig5-figsupp2-data2.zip › Figure 5-figure supplement 2-source data 2/E/MDA-MB-231/tubulin-231-inmune.tif]

Figure 6

D

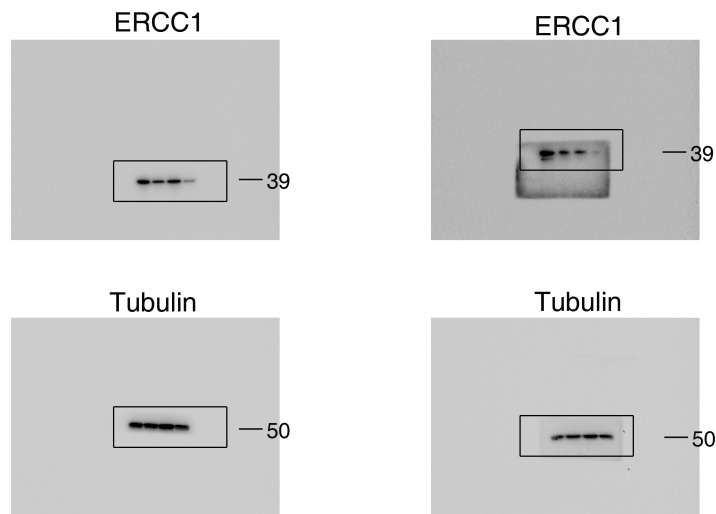

Supplement: Figure 6—source data 1. [file elife-99225-fig6-data1.pdf]

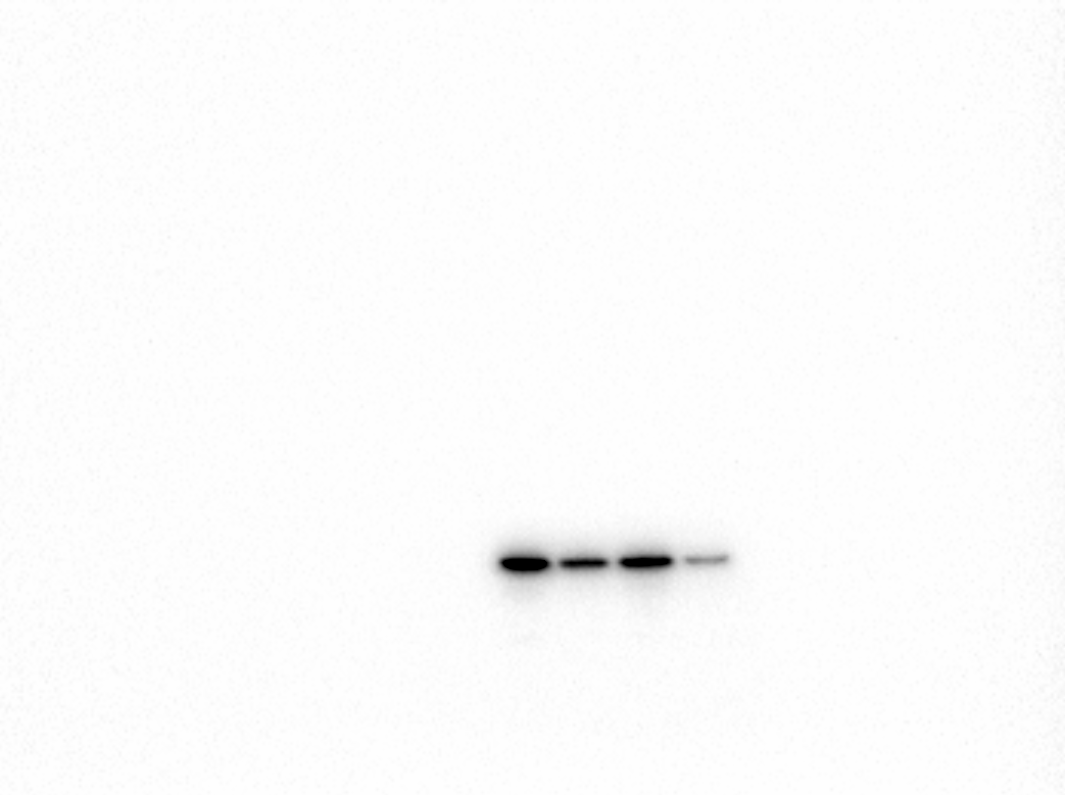

Supplement: Figure 6—source data 2. [file elife-99225-fig6-data2.zip › Figure 6-source data 2/D/OVCAR8/OVCAR8 ERCC1.tif]

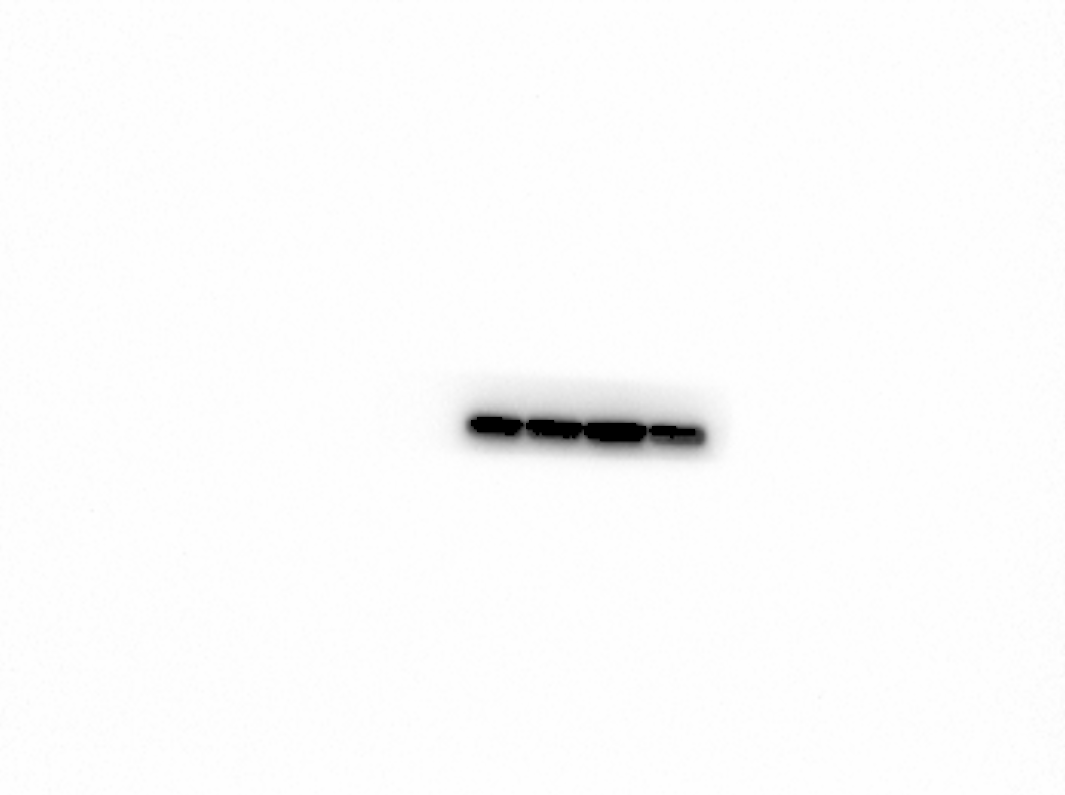

Supplement: Figure 6—source data 2. [file elife-99225-fig6-data2.zip › Figure 6-source data 2/D/OVCAR8/OVCAR8 Tubulin.tif]

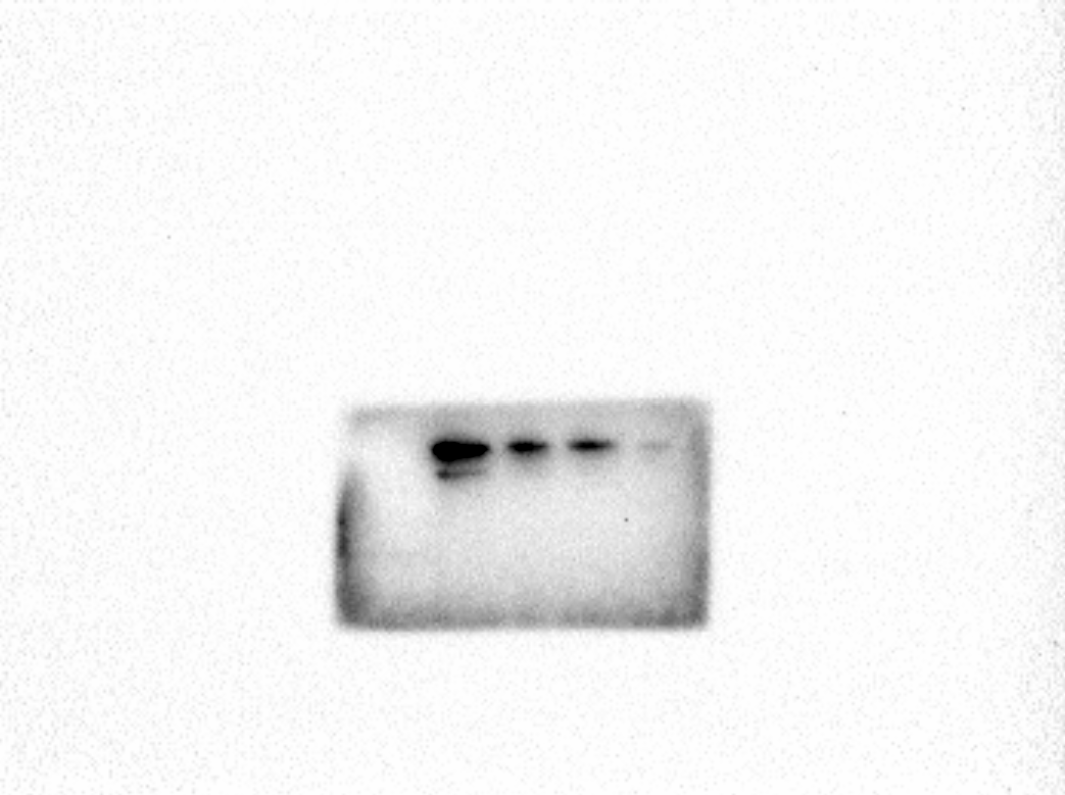

Supplement: Figure 6—source data 2. [file elife-99225-fig6-data2.zip › Figure 6-source data 2/D/MDA-MB-231/MDA-MB-231 ERCC1.tif]

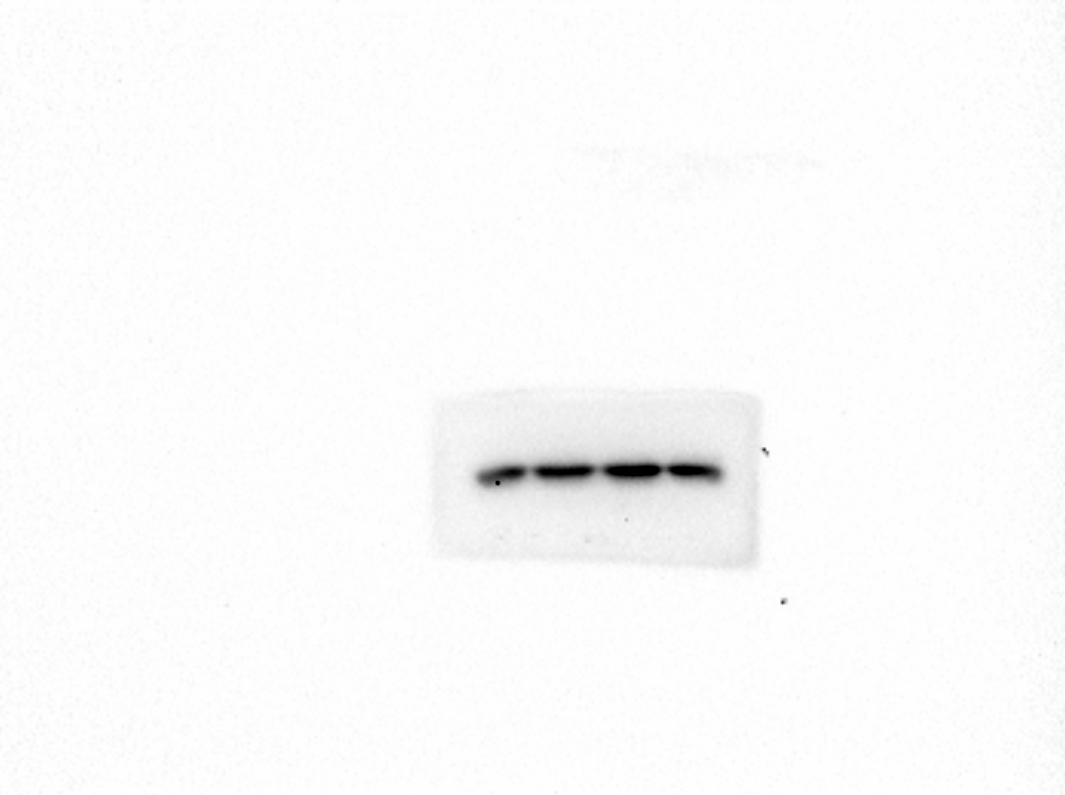

Supplement: Figure 6—source data 2. [file elife-99225-fig6-data2.zip › Figure 6-source data 2/D/MDA-MB-231/MDA-MB-231 Tubulin.tif]
